# Oxidative physiology is weakly associated with pigmentation in birds

**DOI:** 10.1002/ece3.9177

**Published:** 2022-08-11

**Authors:** Attila Marton, Csongor I. Vágási, Orsolya Vincze, Veronika Bókony, Péter L. Pap, Laura Pătraș, Janka Pénzes, Lőrinc Bărbos, Attila Fülöp, Gergely Osváth, Simon Ducatez, Mathieu Giraudeau

**Affiliations:** ^1^ Evolutionary Ecology Group, Hungarian Department of Biology and Ecology Babeș‐Bolyai University Cluj‐Napoca Romania; ^2^ Department of Evolutionary Zoology and Human Biology University of Debrecen Debrecen Hungary; ^3^ Institute of Aquatic Ecology Centre for Ecological Research Debrecen Hungary; ^4^ Lendület Evolutionary Ecology Research Group Plant Protection Institute, Centre for Agricultural Research, Eötvös Loránd Research Network Budapest Hungary; ^5^ Department of Molecular Biology and Biotechnology Babeş‐Bolyai University Cluj‐Napoca Romania; ^6^ Milvus Group Bird and Nature Protection Association Târgu Mureș Romania; ^7^ MTA‐DE Behavioural Ecology Research Group, Department of Evolutionary Zoology and Human Biology University of Debrecen Debrecen Hungary; ^8^ Museum of Zoology Babeş‐Bolyai University Cluj‐Napoca Romania; ^9^ Institut de Recherche pour le Développement (IRD) – UMR 241 EIO (UPF, IRD, Ifremer, ILM) Tahiti French Polynesia; ^10^ Littoral Environnement et Sociétés (LIENSs), UMR 7266 CNRS – La Rochelle Université La Rochelle France

**Keywords:** antioxidant, carotenoid, eumelanin, glutathione, oxidative stress, pheomelanin

## Abstract

The mechanistic link between avian oxidative physiology and plumage coloration has attracted considerable attention in past decades. Hence, multiple proximal hypotheses were proposed to explain how oxidative state might covary with the production of melanin and carotenoid pigments. Some hypotheses underscore that these pigments (or their precursors, e.g., glutathione) have antioxidant capacities or function as molecules storing the toxic excess of intracellular compounds, while others highlight that these pigments can act as pro‐oxidants under specific conditions. Most studies addressing these associations are at the intraspecific level, while phylogenetic comparative studies are still scarce, though needed to assess the generality of these associations. Here, we tested whether plumage and bare part coloration were related to oxidative physiology at an interspecific level by measuring five oxidative physiology markers (three nonenzymatic antioxidants and two markers of lipid peroxidative damage) in 1387 individuals of 104 European bird species sampled during the breeding season, and by scoring plumage eumelanin, pheomelanin, and carotenoid content for each sex and species. Only the plasma level of reactive oxygen metabolites was related to melanin coloration, being positively associated with eumelanin score and negatively with pheomelanin score. Thus, our results do not support the role of antioxidant glutathione in driving variation in melanin synthesis across species. Furthermore, the carotenoid scores of feathers and bare parts were unrelated to the measured oxidative physiology parameters, further suggesting that the marked differences in pigmentation across birds does not influence their oxidative state.

## Abstract 2

Both oxidative physiology and plumage coloration are important foci of avian research in the past decade, and intraspecific studies suggest multiple mechanistic links between the antioxidant system and pigmentation. We performed a large‐scale comparative analysis on 104 European bird species, and found that species‐specific oxidative physiology and plumage coloration evolved largely independently of each other. Out of five oxidative physiological markers measured only the plasma level of reactive oxygen metabolites was related to melanin coloration, being positively associated with eumelanin score, and negatively with pheomelanin score. Thus, our results do not support the role of antioxidant glutathione in driving variation in melanin synthesis across species. Furthermore, the carotenoid scores of feathers and bare parts were unrelated to the measured oxidative physiology parameters, further suggesting that the marked differences in pigmentation across birds does not influence their oxidative state.

## 1 INTRODUCTION

The large variation in bird plumage coloration among individuals, populations, and species has attracted considerable attention from evolutionary biologists (Hill & McGraw, 2006). However, to better understand how this great variety of plumage colors and patterning evolved, it is essential to determine how these colors are produced and how they relate to the individuals' inner state (Hill & Johnson, 2012; McGraw et al., 2010; Weaver et al., 2017). In recent years, there has been an upsurge of interest in the relationship between oxidative physiology and plumage coloration (e.g., Arai et al., 2017; Castiglione et al., 2020; Fernández‐Eslava et al., 2021; Galván, Jorge, et al., 2017; Henschen et al., 2016; Rodríguez‐Martínez & Galván, 2020; Tomášek et al., 2016). Empirical and theoretical studies suggest that the balance between the production of pro‐oxidant molecules (reactive oxygen species, ROS) and antioxidant defenses can modulate the expression of pigment‐based phenotypes, providing a mechanistic link between coloration, individual condition, and health (Garratt & Brooks, 2012; Von Schantz et al., 1999). The two most widespread types of coloration in birds are based on melanin and carotenoid pigments. The synthesis of both pigment types is mechanistically connected with the antioxidant machinery; therefore, melanin‐ and carotenoid‐based plumage colors are expected to be associated to the organism's oxidative status (Henschen et al., 2016; Metcalfe & Alonso‐Alvarez, 2010).

Melanin pigments come in two types: eumelanin (conferring black and gray colors) and pheomelanin (conferring rufous, chestnut, and brown colors; Galván et al., 2011). Different mechanisms were suggested to explain the tight connection between the synthesis of both melanin pigment types and oxidative physiology. First, whether the production of melanins from their precursor dopaquinone takes the route toward eumelanin or pheomelanin depends on the amount of the amino acid cysteine or the cysteine‐containing antioxidant glutathione (GSH), which act therefore as a master switch (Galván & Solano, 2015). High levels of GSH stimulate pheomelanogenesis, while low levels of GSH stimulate eumelanogenesis (Ducrest et al., 2008; Galván et al., 2012; Henschen et al., 2016; Meyskens et al., 1999). As the antioxidant system neutralizes ROS, the level of GSH decreases; hence, a more pheomelanic coloration might covary with increased oxidative stress because pheomelanogenesis might lead to diminished antioxidant capacity by depleting the GSH pool (Galván, Inácio, et al., 2017). A second hypothesis is that both plumage pheomelanin pigments and certain antioxidants such as uric acid may serve as deposits to eliminate the excess dietary amino acids such as cysteine, which can be harmful at high levels (Galván, 2017; Klasing, 1998). Third, ROS are produced during the synthesis of eumelanin, while no or only low amounts of ROS are produced during pheomelanin synthesis (Galván et al., 2014; Galván & Solano, 2015). Finally, melanogenesis is governed by pleiotropic genes (melanocortin system), which also regulate the cellular antioxidant responses (Ducrest et al., 2008; Galván & Alonso‐Alvarez, 2009; Galván & Solano, 2015; San‐Jose & Roulin, 2018).

Carotenoids produce yellow, orange, and red colors (McGraw, 2006a), and unlike melanins, these pigments cannot be synthesized de novo by birds, but must be acquired directly from food (Costantini & Møller, 2008). Besides the coloration function, carotenoids are also hypothesized to act as antioxidants and/or immunostimulants (Lozano, 1994; Olson & Owens, 1998; Pérez‐Rodríguez, 2009; Svensson & Wong, 2011; Vinkler & Albrecht, 2010; Von Schantz et al., 1999, but see Koch & Hill, 2018). Previous studies suggest that carotenoids are subject to a resource allocation trade‐off as their investment into plumage coloration conflicts with their use in physiological processes (the “allocation trade‐off hypothesis,” Lozano, 1994; Olson & Owens, 1998; Pérez‐Rodríguez, 2009; Von Schantz et al., 1999). Consequently, carotenoid coloration might be dependent on nutritional or health condition, while individuals having a balanced redox status are expected to be able to devote substantially more carotenoids to colorize their plumage or integument (Hill & Johnson, 2012; McGraw, 2006a; Møller et al., 2000; Peters et al., 2004). However, this hypothesis has been challenged over the last decade (Hõrak et al., 2010; Koch et al., 2018; Koch & Hill, 2018). First, the importance of carotenoids in the antioxidant machinery has been questioned (Costantini & Møller, 2008; Simons et al., 2012). Second, at high concentrations, the antioxidant activity of carotenoids might shift to pro‐oxidant activity (Hartley & Kennedy, 2004; Huggins et al., 2010; Martin et al., 1999; Palozza, 1998; Palozza et al., 1995; Simons et al., 2014), complicating their potential antioxidant function.

All the above mechanisms suggest that melanin‐ and carotenoid‐based coloration are functionally linked to oxidative physiology: melanin production depends on antioxidants and entails the production of harmful oxidative byproducts, and carotenoids can function both as antioxidants and pro‐oxidants. The mechanistic link between pigmentation and oxidative physiology predicts that the evolution of species‐specific pigmentation cannot be independent of the evolution of species‐specific oxidative physiology. Therefore, as case studies do not allow for generalization, we provide a comparative study based on a large number of species which markedly differ in their melanin and carotenoid coloration, and in concentration of oxidative physiology markers. Oxidative physiology coevolved with ecological and life‐history attributes as part of a pace‐of‐life syndrome (e.g., Vágási et al., 2019), resulting in species‐specific oxidative physiology (Vágási et al., 2016). As pigmentation‐based traits are also subjected to a broad range of selective pressures, we expect that, on an interspecific level, the species‐specific oxidative state may either facilitate or constrain the evolution of the overall plumage coloration or vice versa (Badyaev & Hill, 2000; Emaresi et al., 2014; Saino et al., 2013). Accordingly, we expect certain species‐specific pigmentation phenotypes to evolve in parallel with evolutionary adjustments to the oxidative physiological system (e.g., up‐ or downregulation of particular antioxidants).

To explore whether variation in plumage coloration is associated with variation in redox physiology across species, we conducted a phylogenetic comparative study by measuring three nonenzymatic antioxidant markers (total antioxidant status, TAS; uric acid, UA; and total glutathione, tGSH) and two markers of lipid peroxidation (malondialdehyde, MDA; and reactive oxygen metabolites, ROM) from samples of 1387 individuals belonging to 104 European bird species. We also quantitatively scored the expression of carotenoid, eumelanic, and pheomelanic plumage coloration (Galván et al., 2011; Galván & Møller, 2011), and the carotenoid coloration of bare parts (beak and legs) for each sex of each species. We expected species with a higher proportion of eumelanic coloration and associated higher eumelanin‐mediated ROS production either to evolve an increased antioxidant capacity to preemptively avoid oxidative stress (Ducrest et al., 2008), or to deplete the antioxidant pool while neutralizing ROS. Similarly, we expected species with a higher pheomelanin production either to show elevated levels of GSH, necessary to initiate and sustain pheomelanin production, coupled with an increased protection against oxidative stress as a by‐product of elevated GSH levels, or to face higher levels of oxidative stress due to the depletion of the GSH pool caused by the trade‐off of this antioxidant between plumage pigmentation and antioxidant defense (Galván et al., 2011). Likewise, carotenoids can act both as anti‐ and pro‐oxidants, resulting in either lower or higher species‐specific levels of oxidative stress. Given the above‐mentioned predictions, we aimed to explore whether a relationship between pigmentation type and oxidative status exists at all across the sampled species, as any direction of relationship would provide information on how these traits evolved.

## 2 METHODS

### 2.1 Oxidative physiology data collection

We combined the dataset including 544 individuals from 85 species published in Vágási et al. (2016) with data on an additional 843 individuals from 96 species collected by the same team between 2016 and 2019. Thus, the final oxidative physiology dataset included 1387 individuals belonging to 104 bird species sampled in Romania between 2011 and 2019. Details on the sampling protocol and biochemical assays are provided in Vágási et al. (2016). Briefly, adult individuals were captured and ringed during their breeding season, usually between late April and early July. Most species were captured at multiple locations and/or during multiple years and sampling occasions. We assessed the sex of each individual belonging to sexually dimorphic species based on morphological characters, while most individuals of (apparently) monomorphic species were sexed using molecular methods (for a detailed description of the molecular sexing method see Vincze et al., 2022). We collected blood samples (range 30–300 μl, depending on body size) by brachial venipuncture into heparinized capillaries as fast as possible after the bird was captured (always within 15 min; mean = 9.87 min, SD = 5.37). Blood samples drawn from small‐sized species usually only allowed creating a single aliquot, leading to the measurement of different redox state markers from different samples. Therefore, sample size varies across redox markers within species (see Vágási et al., 2016). All birds were released in good condition after sampling. The samples were kept in dark cooling boxes at around 4 °C for <10 h until centrifuged (for 5 min at 6200*g*) to separate the plasma and erythrocyte fractions. Plasma was partitioned into aliquots for each marker, and all aliquots and the erythrocytes were stored at −50 °C. All laboratory assays were carried out following the same protocol (by LP, JP, and CIV). Detailed protocols for measuring the three antioxidant markers (TAS, UA, and tGSH) and the two lipid peroxidation markers (ROM and MDA) can be found in the Appendix S1. Briefly, we measured TAS as described by Erel (2004), with modifications described in Sepp et al. (2010): nonenzymatic antioxidants decolorize ABTS+ (2,2′‐azino‐bis[3‐ethylbenzothiazoline‐6‐sulfonate]) proportionally with their concentrations in the samples, which can be measured spectrophotometrically at 660 nm, and compared to antioxidants of known concentrations (Trolox, Sigma 2881‐3). We measured UA and tGSH concentrations spectrophotometrically with two commercial assay kits (uricase/peroxidase kit: Uric Acid liquicolor kit, Human, Wiesbaden, Germany; tGSH kit: Sigma‐Aldrich, St. Louis, MO). MDA concentrations were measured by High Performance Liquid Chromatography (HPLC) on a HPLC SUPELCOSIL™ LC‐18 column, while ROM levels were assessed spectrophotometrically by measuring the absorbance of peroxy radicals in reaction with N,N‐diethyl‐para‐phenylenediamine. The five markers of oxidative state showed low, but significant repeatability at the level of species (Table S1), which indicates that variances in these five markers are smaller within species than among species. Trapping by mist nets and blood sampling was performed as licensed by the Romanian Academy of Sciences (permit no. 2257) and in accordance with current animal welfare laws of Romania.

### 2.2 Coloration scoring protocol

We measured the proportion of the entire plumage colored by carotenoid, pheomelanin, and eumelanin pigments for males and females separately for each species by applying the method developed by Galván and Møller (2011) and Galván et al. (2011). For each of the three main pigments, we assigned scores ranging from 0 to 5 to estimate the proportion of plumage color they generated (0 = 0%, 1 = 1–20%, 2 = 21–40%, 3 = 41–60%, 4 = 61–80%, 5 = 81–100%), based on the color plates available from the digital resource *Birds of the World* (Billerman et al., 2020; formerly *Handbook of the Birds of the World Alive*, del Hoyo et al., 2016). Pigmentation leads to distinctive plumage coloration with eumelanin responsible for the black and gray, pheomelanin for the chestnut and brown and carotenoids for the yellow, orange, and red colors (Galván et al., 2011; Galván & Møller, 2011; Hill & McGraw, 2006). Both pheomelanin and eumelanin pigments are often simultaneously deposited in feathers (Galván et al., 2011). However, many bird feathers have an overwhelming proportion of either pheomelanin or eumelanin (i.e., one of the two pigments consisting of >90%; Galván et al., 2011). Interpreting black and gray colors as mostly generated by eumelanin, and brown or chestnut colors as mostly generated by pheomelanin, has therefore been considered adequate to roughly score plumage pigmentation for large‐scale comparative purposes (Galván et al., 2011; Galván & Møller, 2011). The deposition of several pigments in the same feathers (e.g., carotenoids and pheomelanin or carotenoids and eumelanin) can result in green coloration, therefore we excluded nine species from our initial dataset. Besides carotenoid and melanin pigments, other rare pigments (e.g., porphyrins, psittacofulvins, pterins, flavins, and other, undescribed pigments) can also contribute to plumage coloration (McGraw, 2006b). Given that our database contains only two species of Strigiformes (tawny owl *Strix aluco* and little owl *Athene noctua*) where porphyrins have been reported, these rare pigments are unlikely to distort our results. Although this coloration scoring method only provides a rough estimation of the plumage pigmentation, it is considered appropriate for comparative analyses due to the large across‐species variation that ensures repeatability at the level of species (Galván et al., 2011; Galván & Møller, 2011; Owens & Hartley, 1998; Seddon et al., 2010).

All species were scored within a large‐scale plumage pigmentation scoring endeavor comprising more than 7000 species, by one of two observers (SD or MG). To evaluate the consistency of the scoring, 216 randomly chosen species were scored by both observers. All three pigmentation scores were highly repeatable between the two observers (weighted Cohen's Kappa for ordinal variables, eumelanin: Kappa = 0.824; pheomelanin: Kappa = 0.833; carotenoid: Kappa = 0.914; all *p* < .001). The presence of carotenoids in the beak and legs was also assessed using the same plates by the same observers. Species received a score of 0 if carotenoids were absent, and a score of 1 if carotenoids were present in the beak (repeatability = 0.983 ± 0.002) or legs (repeatability = 0.797 ± 0.056; repeatability estimates and likelihood ratio test results obtained using the generalized linear mixed model and logit link‐scale approximation with the *rpt* function for binary data in the R package “rptR,” Stoffel et al., 2017). Pigmentation scores are not independent of each other, as each of the three scores expresses the proportion of the plumage colored by a given pigment (e.g., if 80–100% of a bird plumage is colored by pheomelanin, then carotenoid/eumelanin score can only reach 0–20%). We thus conducted a principal components analysis (PCA) to extract independent variables describing plumage pigmentation. The PCA was based on the proportion of the three pigments and simultaneously included data from the two sexes (i.e., including one datum per sex per species). Two components with eigenvalues >1 were extracted from the PCA on the pigmentation scores. The first component (PC1) explained 62.39% and the second (PC2) 32.38% of the total variance in pigmentation. The two melanin pigments loaded on PC1, with eumelanin having a positive (0.908) and pheomelanin a negative loading (−0.958). Carotenoid coloration only weakly loaded on the PC1 (0.358), but it was the only variable with a strong positive loading on the PC2 (0.930). Therefore, PC1 is interpreted as the melanin‐based coloration axis, and PC2 as the carotenoid‐based coloration axis.

### 2.3 Confounding variables

We included body mass and diet as potentially confounding variables in the analyses (see below), as these were previously shown to affect the antioxidant status of wild animals (Cohen et al., 2009; Costantini, 2008; Olson & Owens, 2005; Tella et al., 2004; Vágási et al., 2019). Body mass data for each sex of each species was extracted from Storchová and Hořák (2018), with minor modifications due to differences in mass between subspecies (changes are highlighted in the data frame indicated in the Data Accessibility Statement). Diet during the breeding season was also collected from Storchová and Hořák (2018) and categorized into a three‐level factor coded as animal‐based, plant‐based, or omnivorous (i.e., both animal‐ and plant‐based food present in the breeding diet).

### 2.4 Phylogenetic comparative analyses

To test for associations between oxidative physiology and pigmentation, we built phylogenetic linear mixed models (PLMMs) based on Markov chain Monte Carlo (MCMC) estimations, as implemented in the “MCMCglmm” package (Hadfield, 2010) in the R statistical and computing environment (v. 4.1.1; R Core Team, 2020). Individual levels of TAS, UA, MDA, and ROM values were square‐root transformed, while tGSH was log10‐transformed to provide the best model fit for each, and were used as response variables in separate models, while coloration variables were used as explanatory variables. We chose this approach for several reasons. First, since we have individual‐level data for oxidative markers, using individuals as the unit of analysis with the oxidative markers as response variables yields the most powerful way of maximizing information gain and not ignoring within‐species variation (Garamszegi, 2014). Second, using oxidative markers as explanatory variables could lead to multicollinearity and thereby unreliable results (Graham, 2003). To assess the degree of multicollinearity, we constructed linear models with oxidative parameters used as response variables, and with PC1 and PC2 describing plumage pigmentation, presence of carotenoids in the beak and in the legs, diet, body mass (log_10_‐transformed), and sex (as a three‐level factor: female, male, or unknown) as explanatory variables (see R Markdown file in the Appendix S1). However, variation inflation factor (VIF) values for our models were <2, which suggests that multicollinearity could not distort our results. Third, the phylogenetic signal of all oxidative markers was low (Table S2), suggesting that the correlation between each marker and coloration can be tested equally well by taking either variable as response (as described in Appendix S3 of Liker et al., 2021). Note that the direction of cause–effect relationships may go both ways, that is, while oxidative physiology may constrain the evolution of coloration, selection on coloration may also drive the evolution of oxidative physiology, and PLMMs can only test for evolutionary correlations.

Each PLMM included the species' PC1 and PC2 describing plumage pigmentation, presence of carotenoids in the beak and in the legs, diet, body mass (log_10_‐transformed), and sex as explanatory variables. In the model of TAS, UA was additionally included as covariate because UA has a large contribution to TAS (Cohen et al., 2008). Since for most species several individuals were sampled from several years, species identity and year were included in the models as random intercepts. In addition, we controlled for phylogenetic relationships among species by including phylogeny in the random structure of the models. For this phylogenetic control we used a consensus tree, created by applying the *consensus.edges* function of the “phytools” R package (Revell, 2012) on 1000 phylogenies sampled from the pseudo‐posterior distribution available on www.birdtree.org (Jetz et al., 2012). MCMC chains were run for 2,500,001 iterations with a burn‐in interval of 50,000. A total of 10,000 iterations were sampled (i.e., each 245th iteration) to estimate parameters for each model. Autocorrelation levels among sampled iterations were lower than the more conservative threshold level of 0.1, and we used graphic visualization of all posterior distributions to assess model convergence. We used Gaussian distributions and priors that equally partitioned the variance in each oxidative state marker (i.e., response variable) between the four random terms: phylogeny, species, year, and residual variance.

We tested the robustness of our results by re‐analysis in three ways: (1) using a subset of data excluding species with iridescent plumage (eight species), (2) using a subset of data containing only species with a sample size of *N* ≥ 3, or (3) using an alternative phylogenetic tree (Cooney et al., 2017). Moreover, we performed further sensitivity analyses for the two response variables for which we had directional predictions (tGSH and ROM), by replacing PC1 and PC2 with either raw eumelanin or raw pheomelanin scores and re‐running all four models described above (i.e., two models on the full dataset with two different phylogenetic trees, and two models with subsets of data containing species without iridescent plumage and with a sample size of *N* ≥ 3).

## 3 RESULTS

Plumage and bare part pigmentation were not significantly associated with any of the tested markers of oxidative status, except for ROM levels (Table 1). Plasma ROM concentrations were positively associated with a more eumelanic and less pheomelanic coloration (i.e., higher PC1 values), but were not related to carotenoid coloration (i.e., PC2 values). In models where PC1 was substituted with raw eumelanin and pheomelanin scores, species with proportionally higher eumelanic coloration exhibited marginally significantly higher ROM levels and species with more pheomelanic plumage exhibited marginally significantly lower ROM levels (Table 2). The relationship between ROM and PC1 or melanin scores was significantly strong and marginally nonsignificant, respectively, with an alternative phylogeny. This relationship weakened when species with iridescent plumage were excluded (marginally significant between ROM levels and PC1, and nonsignificant between ROM and raw eumelanin or pheomelanin scores), and stronger for all three melanin variables when only species with *N* ≥ 3 individuals were included in the models (Tables S3–S9). All other relationships between coloration variables and oxidative markers remained nonsignificant in the sensitivity analyses (see Tables S3–S9).

**TABLE 1:** Results of Markov chain Monte Carlo (MCMC) phylogenetic linear mixed models showing the relationship of three antioxidant markers (total antioxidant status, TAS; uric acid, UA; and total glutathione, tGSH) and two markers of lipid peroxidation (malondialdehyde, MDA; and reactive oxygen metabolites, ROM) with plumage melanin (PC1) and carotenoid (PC2) pigment scores, diet, and body mass. Model parameters (posterior mean with 95% credibility intervals, CrI) were estimated based on 10,000 iterations of each model. Fixed effects with *p*_MCMC_ <.05 are highlighted in bold.

	TAS				UA				tGSH			
	Post. Mean	Lower CrI	Upper CrI	*p*_MCMC_	Post. Mean	Lower CrI	Upper CrI	*p*_MCMC_	Post. Mean	Lower CrI	Upper CrI	*p*_MCMC_
Fixed effects												
Intercept	0.900	0.585	1.180	<0.001	4.924	3.927	5.960	<0.001	0.607	0.332	0.882	<0.001
UA	0.073	0.057	0.091	**<0.001**								
PC1	0.001	−0.018	0.020	0.903	−0.044	−0.125	0.038	0.291	0.004	−0.016	0.024	0.690
PC2	0.003	−0.024	0.035	0.840	−0.042	−0.167	0.075	0.502	0.002	−0.027	0.032	0.914
Beak carotenoid	0.055	−0.040	0.141	0.242	−0.123	−0.525	0.260	0.542	0.004	−0.091	0.101	0.928
Leg carotenoid	−0.067	−0.177	0.047	0.244	0.008	−0.482	0.477	0.985	−0.021	−0.130	0.104	0.723
Body mass	−0.007	−0.105	0.091	0.894	−0.369	−0.786	0.077	0.095	0.125	0.027	0.230	**0.018**
Diet (omnivore)	−0.058	−0.155	0.041	0.248	−0.180	−0.598	0.266	0.407	0.077	−0.021	0.182	0.128
Diet (herbivore)	0.028	−0.086	0.140	0.630	−0.511	−1.039	0.030	0.063	0.158	0.033	0.277	**0.013**
Sex (male)	0.009	−0.035	0.056	0.701	0.078	−0.087	0.226	0.331	−0.001	−0.043	0.043	0.968
Sex (unknown)	0.007	−0.078	0.093	0.861	−0.242	−0.530	0.060	0.109	−0.097	−0.179	−0.023	**0.014**
Random effects												
Phylogeny	0.007	0.002	0.014		0.186	0.030	0.403		0.012	0.003	0.023	
Species	0.005	0.002	0.009		0.175	0.065	0.287		0.006	0.002	0.011	
Year	0.067	0.013	0.160		0.197	0.029	0.496		0.044	0.009	0.104	

	MDA				ROM			
	Post. mean	Lower CrI	Upper CrI	*p*_MCMC_	Post. mean	Lower CrI	Upper CrI	*p*_MCMC_
Fixed effects								
Intercept	2.230	1.791	2.659	<0.001	1.651	1.194	2.110	<0.001
PC1	−0.010	−0.037	0.016	0.457	0.040	0.004	0.075	**0.024**
PC2	−0.015	−0.054	0.023	0.450	0.032	−0.020	0.083	0.226
Beak carotenoid	0.002	−0.122	0.133	0.973	0.002	−0.173	0.166	0.984
Leg carotenoid	0.088	−0.073	0.253	0.292	0.080	−0.130	0.283	0.444
Body mass	−0.183	−0.320	−0.041	**0.010**	−0.193	−0.365	−0.013	**0.029**
Diet (omnivore)	−0.107	−0.250	0.030	0.134	0.152	−0.016	0.326	0.085
Diet (herbivore)	−0.215	−0.389	−0.037	**0.022**	0.079	−0.116	0.273	0.434
Sex (male)	−0.015	−0.068	0.035	0.565	−0.005	−0.100	0.091	0.916
Sex (unknown)	0.005	−0.089	0.107	0.920	0.108	−0.025	0.238	0.109
Random effects								
Phylogeny	0.027	0.007	0.052		0.017	0.004	0.037	
Species	0.014	0.005	0.024		0.010	0.003	0.018	
Year	0.156	0.030	0.360		0.073	0.004	0.215	

**TABLE 2:** Results of Markov chain Monte Carlo (MCMC) phylogenetic linear mixed models showing a positive association between plumage eumelanin score and plasma reactive oxygen metabolites (ROM), and negative association between plumage pheomelanin score and plasma ROM. Model parameters (posterior mean with 95% credibility intervals, CrI) were estimated based on 10,000 iterations of each model. Fixed effects with *p*_MCMC_ <.05 are highlighted in bold.

	ROM in relation to eumelanin				ROM in relation to pheomelanin			
	Post. Mean	Lower CrI	Upper CrI	*p*_MCMC_	Post. Mean	Lower CrI	Upper CrI	*p*_MCMC_
Fixed terms								
Intercept	1.656	1.194	2.098	<.001	1.735	1.266	2.193	<.001
Eumelanin	0.035	0.000	0.069	.051				
Pheomelanin					−0.028	−0.056	0.000	.052
Beak carotenoid	−0.011	−0.183	0.160	.892	−0.007	−0.180	0.162	.935
Leg carotenoid	0.094	−0.116	0.304	.388	0.082	−0.127	0.292	.445
Body mass	−0.238	−0.411	−0.064	**.008**	−0.203	−0.369	−0.030	**.020**
Diet (omnivore)	0.192	0.022	0.358	**.026**	0.176	0.011	0.345	**.039**
Diet (herbivore)	0.109	−0.089	0.296	.264	0.108	−0.094	0.291	.266
Sex (male)	0.001	−0.100	0.095	.993	0.007	−0.086	0.107	.882
Sex (unknown)	0.105	−0.035	0.236	.128	0.109	−0.025	0.247	.119
Random terms								
Phylogeny	0.019	0.004	0.040		0.019	0.004	0.040	
Species	0.010	0.003	0.019		0.010	0.003	0.019	
Year	0.077	0.003	0.225		0.076	0.003	0.223	

Predictors other than coloration were significantly related to oxidative state markers as shown in Table 1. Species with a plant‐based diet had lower MDA but higher tGSH levels than carnivorous species. MDA and ROM decreased significantly with body mass, while tGSH levels were positively associated with body mass, similarly to previous results described in Vágási et al. (2016, 2019). TAS levels were related only to UA levels. These results remained qualitatively unchanged in the sensitivity analyses (see Tables S3–S9).

## 4 DISCUSSION

Our study suggests that avian coloration and oxidative physiology are only weakly associated across species and thus evolved largely independently of each other. Melanin‐based coloration was associated with a marker of oxidative stress as species with a higher proportion of eumelanic plumage (and lower proportion of pheomelanic plumage) had higher ROM levels. However, this relationship was marginally significant when the principal component axis of melanin‐based coloration was substituted with raw eumelanin and pheomelanin scores, and these relationships were relatively weak, particularly when the species with iridescent plumage (7.7% of the initial dataset, which also have a more eumelanic plumage) were excluded from the analyses. The extent of eumelanin coloration was associated neither with an increase in antioxidant defense levels, nor with the depletion of the antioxidant pool. Neither was the extent of pheomelanin coloration associated with reductions in the levels of glutathione across species. The extent of carotenoid coloration of the plumage and of bare parts was unrelated to markers of oxidative physiology, in line with findings of recent intraspecific studies, which together align with the idea that carotenoids are not a key component of the antioxidant machinery (Hõrak et al., 2010; Koch et al., 2018; Koch & Hill, 2018).

The chemical pathway of pheomelanogenesis consumes cysteine that is free or provided by the most powerful intracellular nonenzymatic antioxidant, the cysteine‐containing tripeptide glutathione (Potterf et al., 1999). Intraspecific studies suggest that the production of pheomelanin may involve the depletion of cysteine via glutathione (e.g., Arai et al., 2017; Galván et al., 2015; Leclaire et al., 2019; Rodríguez‐Martínez & Galván, 2019; Schallreuter et al., 2008). However, our interspecific results do not support this hypothesis, as we found no relationship between glutathione levels (tGSH) and pheomelanin coloration expressed either as PC1 or as raw pheomelanin scores. This lack of association is unlikely to be due to small statistical power given the large sample size for glutathione levels (1387 individuals of 104 species). This lack of association might stem from the fact that we measured total glutathione levels, which contains the amount of both reduced and oxidized glutathione (GSH and GSSG, respectively, see Appendix S1 of Vágási et al., 2016). The contribution of GSSG to the tGSH concentration is however negligible, suggesting that measuring tGSH is unlikely to bias our results. Indeed, in physiological conditions, the GSH:GSSG molar ratio is between 100:1 and 1000:1 (discussed in detail by Monostori et al., 2009), while the proportion of GSSG in erythrocytes represents less than 0.5% of the GSH concentration (Reinbold et al., 2014). For future studies, the GSH:GSSG ratio might still bring additional information regarding the availability of glutathione to act as an antioxidant (Galván et al., 2014). In conclusion, our results suggest that the mechanisms linking pheomelanogenesis, glutathione, and cysteine may be more complex than a simple allocation trade‐off of cysteine between pheomelanin synthesis and GSH‐mediated antioxidant defense.

A toxic excess in cysteine occurs when food cysteine content is higher than the amount needed for protein synthesis, exerting a negative influence on physiological pathways and growth, and causing metabolic acidosis and eggshell thinning (Galván, 2017; Klasing, 1998). Thus, species with a diet rich in cysteine might have evolved increased pheomelanin coloration to remove the surplus cysteine from the body and to deposit it into their feathers, which are dead structures (Galván, 2017; Galván et al., 2012). Under this hypothesis, an interspecific relationship between the extent of pheomelanin coloration and glutathione levels is not expected. Importantly, however, excess dietary amino acids are also diverted to the synthesis of uric acid, the main product of protein breakdown and excretion in birds (Klasing, 1998). In accordance with this hypothesis, we found that species with a carnivorous diet exhibited on average higher uric acid levels. Our study also reveals that species with more pheomelanic pigments in their plumage do not circulate significantly higher levels of uric acid, suggesting that species potentially suffering from excess cysteine might not use either the synthesis of pheomelanin or an increased production of uric acid to limit cysteine toxicity. Although we found that species with a plant‐based diet, which is low in both proteins and cysteine, exhibited higher tGSH levels (i.e., a larger amount of bound cysteine), future studies should investigate if species with increased pheomelanin plumage coloration also feed on a diet rich in cysteine.

The finding that species with a higher proportion of eumelanic coloration showed higher concentration of ROM across species supports the hypothesis put forward by García‐Molina et al. (2005) and tested experimentally by Galván and Alonso‐Alvarez (2008, 2009). According to this hypothesis, some ROS (e.g., H_2_O_2_, a principal component of the ROM assay) promote eumelanin production in the early stages of melanogenesis. In these experiments performed on red‐legged partridges *Alectoris rufa* and great tits *Parus major*, the expression of eumelanin‐based plumage signals was enhanced by the α‐melanocyte‐stimulating hormone and by GSH inhibition (Galván & Alonso‐Alvarez, 2008), externally induced oxidative stress, or the additive effect of these two manipulations (Galván & Alonso‐Alvarez, 2009). Plumage melanin pigmentation is at least partly attributed to variation in melanocortin levels, and melanocortins have pleiotropic effects (Ducrest et al., 2008): α‐MSH is known to inhibit GSH‐peroxidase in keratinocyte and melanoma cell lines (Haycock et al., 2000). Therefore, by inhibiting the GSH‐peroxidase, α‐MSH could also contribute to elevated ROS levels. However, further studies are needed to ascertain the generality and mechanisms of the relationships between oxidative stress and melanin‐based coloration, because the interspecific correlations we found between ROM and melanic colorations showed some degree of uncertainty (e.g., sensitivity to excluding species with iridescent coloration), and we detected no correlation between melanization and MDA, another marker of oxidative damage. The reasons for this heterogeneity would be best addressed by a combination of experimental work and cross‐species comparisons, because comparative studies can neither assess causality, nor can they explicitly test for trade‐offs due to the “big house – big car” paradox (Reznick et al., 2000).

It is important to note that the samples we used to measure oxidative physiology were taken during the breeding season for all individuals, while coloration is developed during molt. Given that these two energetically demanding activities rarely overlap, the lack of association could originate from species‐specific differences in the seasonal changes in oxidative physiology. However, if species‐specific life histories entail a certain species‐specific redox state (as shown in Vágási et al., 2016, 2019), and this redox state constrains the evolution of coloration (or vice versa), within species differences in redox state due to seasonal changes should be smaller than the magnitude of differences detected across species. Future studies should explore the seasonal change in oxidative physiology across bird species and test for the potential associations between oxidative stress markers during the molting season and the resulting plumage melanin pigmentation. Note that seasonal color change cannot explain the lack of relationship between oxidative physiology and carotenoid‐based coloration of bare parts, because the latter is not generated during the molting season.

Carotenoid‐based traits are considered to honestly signal individual quality, because carotenoids may be limited in nature and thus birds may face a trade‐off when allocating carotenoids for pigmentation and for building and maintaining immune and antioxidant defenses (Olson & Owens, 2005; Simons et al., 2012, 2014; Svensson & Wong, 2011). However, a growing body of within‐species experimental studies (e.g., Hõrak et al., 2010; Koch et al., 2018) and meta‐analyses (Costantini & Møller, 2008; Simons et al., 2012) challenges the importance of carotenoids as antioxidants (reviewed by Hill, 2011; Koch & Hill, 2018) and suggests instead that carotenoid signal honesty is maintained through the cellular respiratory system (Johnson & Hill, 2013; Hill, 2014). Here, we found no association between the circulating antioxidant levels and plumage or integument carotenoid pigmentation on an interspecific level, which seems to support the idea that carotenoid‐based traits are not tightly linked with antioxidative physiology. However, the correlative nature of interspecific comparative studies did not allow us to directly test for the existence of the hypothesized allocation trade‐off, and intraspecific studies likely remain the key to further elucidate this question.

In conclusion, our study highlights that species‐specific oxidative physiology is largely unrelated to plumage pigmentation despite multiple predictions for a tight link between the antioxidants and pigment synthesis. Nevertheless, species with more eumelanic and less pheomelanic plumage may experience higher oxidative stress as measured by the concentration of reactive oxygen metabolites. Given the complexity of the pathways in which both melanin and carotenoid pigments interact with other physiological pathways, the nature of associations between coloration and physiology needs further correlative and experimental research to help us understand their co‐evolution across the tree of life.

## AUTHOR CONTRIBUTIONS

**Attila Marton:** Data curation (equal); formal analysis (supporting); funding acquisition (supporting); resources (equal); writing – original draft (equal); writing – review and editing (lead). **Orsolya Vincze:** Data curation (equal); formal analysis (equal); funding acquisition (equal); resources (equal); writing – original draft (equal); writing – review and editing (equal). **Veronika Bókony:** Formal analysis (equal); funding acquisition (supporting); writing – original draft (equal); writing – review and editing (equal). **Péter L. Pap:** Funding acquisition (equal); resources (equal); writing – review and editing (equal). **Laura Pătraș:** Resources (equal). **Janka Pénzes:** Funding acquisition (equal); resources (equal). **Lőrinc Bărbos:** Resources (equal). **Attila Fülöp:** Resources (equal); writing – review and editing (supporting). **Gergely Osváth:** Resources (equal); writing – review and editing (supporting). **Simon Ducatez:** Conceptualization (equal); data curation (equal); methodology (equal); writing – original draft (equal); writing – review and editing (equal). **Mathieu Giraudeau:** Conceptualization (lead); data curation (equal); methodology (equal); writing – original draft (equal); writing – review and editing (equal). **Csongor I. Vágási:** Conceptualization (lead); data curation (lead); funding acquisition (equal); methodology (equal); project administration (equal); resources (equal); supervision (lead); writing – original draft (equal); writing – review and editing (equal).

## CONFLICT OF INTEREST

The authors declare no conflict of interest.

### OPEN RESEARCH BADGES

This article has earned Open Data, Open Materials and Preregistered Research Design badges. Data, materials and the preregistered design and analysis plan are available at https://figshare.com/s/49d792c230cd3e1d08fb, DOI: 10.6084/m9.figshare.20309556.

## Supporting information


Appendix S1

## Data Availability

The dataset used for this study is freely available for download from Figshare (https://figshare.com/s/49d792c230cd3e1d08fb, DOI: 10.6084/m9.figshare.20309556). The R Markdown file generated for the analysis and visualization of the data is included in the Appendix S1.
