# Supplementary material for: Oxidative physiology is weakly associated with pigmentation in birds
Source: Ecol Evol. 2022 Aug 11;12(8):e9177. doi: 10.1002/ece3.9177 (PMC9366753; doi:10.1002/ece3.9177)
Supplement: Supplementary file 1 — Appendix S1 [file ECE3-12-e9177-s001.pdf]

Oxidative physiology is weakly associated with pigmentation in birds

- Supplementary Material -

Attila Marton, Csongor I. Vágási, Orsolya Vincze, Veronika Bókony, Péter L. Pap,

Laura Pătraş, Janka Pénzes, Lőrinc Bărbos, Attila Fülöp, Gergely Osváth, Simon Ducatez, Mathieu Giraudeau

Corresponding author:

Csongor I. Vágási: csvagasi@gmail.com

Mathieu Giraudeau: giraudeau.mathieu@gmail.com

Methods

Biochemical assays

We measured total antioxidant status (TAS), uric acid (UA), malondialdehyde (MDA) and reactive oxygen metabolite (ROM) levels from plasma, and total glutathione (tGSH) levels from erythrocytes using blood samples collected from 104 European bird species.

We measured TAS as described by (Erel 2004), with modifications as per Sepp et al. (2010). This assay relies on the ability of non-enzymatic antioxidants (e.g., UA, vitamins, sulfhydryl groups of proteins, GSH) to decolorize the blue-green ABTS+ (2,2'-azino-bis(3-ethylbenzothiazoline-6-sulfonate)) to a degree proportional to their concentrations, which can be measured spectrophotometrically at 660 nm. Briefly, we measured the first absorbance level after mixing 5 µL of plasma with 200 µL of 0.4 M acetate buffer (pH 5.8), as sample blank, and the last absorbance level 20 min after incubating the previous mix with 20 µL of 10 mM ABTS+ (Sigma A1888) in 30 mM acetate buffer (pH 3.6). We used an antioxidant of known concentration (Trolox, Sigma 23,881-3) as a standard for calculating the antioxidant levels in the samples, as such, assay results are expressed as mM Trolox equivalents. Repeatability of within and between plates in a subset of samples was very high (within plate: intraclass correlation coefficient, ICC = 0.91, 95% CI = 0.82–1.00, $F_{13,14} = 21.7$, $P < 0.001$; between plate: ICC = 0.94, 95% CI = 0.89–0.99, $F_{13,28} = 52.0$, $P < 0.001$). UA is a major component of TAS (species-level correlation: $r = 0.32$, 95% CI: 0.12–0.50, $t = 3.07$, df = 82, $P = 0.003$) in agreement with previous studies (Cohen et al. 2008).

We measured plasma UA concentration spectrophotometrically from 5 µL of plasma with an uricase/peroxidase method (Uric Acid liquicolor kit, Human, Wiesbaden, Germany). Results are given as mg/dL plasma (see details and repeatability in Bókony et al. (2014)).

We measured tGSH levels using a commercial assay kit (Sigma-Aldrich, St Louis, MO) as described in Galván & Alonso-Alvarez (2008) and Hõrak et al. (2010) with the following modifications: after thawing on ice, we washed the erythrocyte pellet three times with phosphate-buffered saline and centrifuged it at 600 g for 10 min at 4°C. Then,

we weighed the pellets (± 0.001 mg) and deproteinized them with 5% 5-sulfosalicylic acid (SSA; 1:1 w/v). For example, a 100 mg pellet was diluted in 100 µL of 5% SSA. We kept the solution on ice after vortexing it for 10 min, and then centrifuged it at 10,000 g for 10 min at 4°C to remove the precipitated proteins. The supernatant (5 µL) was transferred to another test tube, diluted 10× and used subsequently for tGSH detection, according to the manufacturer's instructions. The determination of tGSH is based on a kinetic assay in which nanomoles of GSH cause a continuous reduction of 5,5′-dithiobis(2-nitrobenzoic acid) to 5-thio-2-nitrobenzoic acid (TNB) and the GSH oxidized to GSSG is subsequently recycled by glutathione reductase and reduced nicotinamide adenine dinucleotide phosphate (NADPH). The yellow colour of the TNB product is proportional to the GSH concentration, and its absorbance was measured spectrophotometrically at 412 nm at 1 min intervals for 5 min. The change in absorbance was compared with that of a standard curve (R2 = 0.99) generated by serial dilution of reduced GSH. Results are given in nM/mg of pellet. We presented data about the high repeatability of measures in Bókony et al. (2014).

We measured MDA concentration in 10 µL of plasma by High Performance Liquid Chromatography (HPLC) on a HPLC SUPELCOSILTM LC-18 column (5 µm particle size; Sigma-Aldrich) with UV detection at 254 nm (Jasco, UV-2075 Plus, Japan), as described in Bókony et al. (2014). The mobile phase was 30 mM monopotassium phosphate (KH2PO4)–methanol (65:35, v/v %) and the flow rate 0.5 mL/min. The retention time of MDA recorded was around 6 min. We determined MDA concentrations in the samples using a calibration curve (R2 = 0.99) of a series of standards generated by acidic hydrolysis of 1,1,3,3-tetraethoxypropane (TEP; Sigma-Aldrich). Results are given as µg/mL plasma and are not corrected for the dilution factor (see Bókony et al. (2014) for details and repeatability).

ROM quantifies mainly hydroperoxides produced by the oxidative damage of biomolecules (chiefly lipids, but also proteins and nucleic acids) and is based on the Fenton reaction, which produces alkoxy and peroxy radicals. The latter react with the N,N-diethyl-para-phenylenediamine, creating a red-purple colored complex. The color of this complex is directly proportional with the amount of hydroperoxides in the sample. ROM levels in plasma were measured following the method employed by Brambilla et al. (2001), with modifications according to Noguera et al. (2011). Briefly, 200 µL of 0.1 M acetate buffer (pH 4.8) were added to 5 µL of plasma and following a 1 min incubation on a thermo-shaker, the absorbance was measured as sample blank. Then, 5 µL of a chromogen solution containing 0.37 M N,N-diethyl-para-phenylenediamine (Sigma 261513) diluted in ethanol were added, followed by incubation for 75 min at 37 °C in the dark with gentle shaking. The absorbance of the colored complex was measured at 505 nm and the obtained values were corrected for the sample blank and then plotted against a calibration curve created by using a dosed activity of 4.23 mM H2O2. ROM levels are expressed as mM H2O2 equivalents. This assay measures the standing ROM levels, i.e., those levels affected by antioxidant defenses and ROM production (Noguera et al., 2011). Repeatability of ROM within and between plates in a subset of samples was very high (within plate: intra-class correlation coefficient, ICC = 0.90, 95% confidence interval, CI of ICC = 0.81–0.98, F21,22 = 18.2, P < 0.0001; between plates: ICC = 0.90, 95% CI of ICC = 0.81–1.00, F15,16 = 19.9, P < 0.0001).

The five markers of oxidative state showed low but significant repeatability at the level of species (Table S1 of Supplementary Material).

Results

Repeatability of oxidative physiology measurements

Table S1. Within-species and within-year repeatability testing. n – number of sampled species, R – repeatability value, 95% lower and upper CI – confidence interval.

	Species					Year				
**OXIDATIVE marker**	**n**	**R**	**95% lower CI**	**95% upper CI**	**P**	**n**	**R**	**95% lower CI**	**95% upper CI**	**P**
**TAS**	104	0.029	0.006	0.066	0.002	7	0.289	0.076	0.489	<0.001
**UA**	104	0.165	0.107	0.230	<0.001	7	0.043	0.002	0.107	0.015
**MDA**	104	0.099	0.055	0.167	<0.001	7	0.388	0.103	0.595	<0.001
**TGSH**	104	0.119	0.069	0.175	<0.001	7	0.198	0.043	0.393	<0.001
**ROM**	79	0.063	<0.001	0.136	0.018	3	0.049	<0.001	0.171	0.031

Phylogenetic signal of oxidative physiology measurements

Table S2. We estimated phylogenetic signal (λ) for each oxidative physiology measurement, in two alternative ways: in the first approach, we took λ as the proportion of variance explained by the phylogeny out of all variances in the data, treating within-species variance as a biologically relevant, species-specific trait; in the second approach, we exclude the within-species variance, treating it as measurement error (noise). Highest posterior density (HPD) interval is calculated from the 95% credibility interval.

**Within-species variance treated as:**	**Biologically relevant**			**Measurement error**		
	**λ**	**lower HPD**	**upper HPD**	**λ**	**lower HPD**	**upper HPD**
**TAS**	0.036	0.013	0.081	0.087	0.019	0.261
**UA**	0.108	0.027	0.226	0.306	0.132	0.681
**MDA**	0.091	0.024	0.206	0.098	0.031	0.426
**tGSH**	0.130	0.046	0.205	0.330	0.102	0.569
**ROM**	0.054	0.008	0.170	0.266	0.038	0.639

Sensitivity analyses

Table S3. Results of Markov chain Monte Carlo (MCMC) phylogenetic linear mixed models showing the relationship of three antioxidant markers (TAS, UA, tGSH) and two markers of lipid peroxidation (MDA, ROMs) with plumage melanin (PC1) and carotenoid (PC2) pigment content, diet, and body mass, performed on a subset of data from which data of 8 species (common kingfisher *Alcedo atthis*, Eurasian roller *Coracias garrulus*, red-rumped swallow *Hirundo daurica*, barn swallow *Hirundo rustica*, blue tit *Parus caeruleus*, magpie *Pica pica*, Eurasian starling *Sturnus vulgaris*, and rosy starling *Sturnus roseus*) with iridescent plumage were excluded. Model parameters (posterior mean with 95% credibility intervals, CrI) were estimated based on 10,000 iterations of each model. Fixed effects with PMCMC < 0.05 are highlighted in bold.

	**TAS**				**UA**				**tGSH**			
	**Post. mean**	**Lower CrI**	**Upper CrI**	**P_{MCMC}**	**Post. mean**	**Lower CrI**	**Upper CrI**	**P_{MCMC}**	**Post. mean**	**Lower CrI**	**Upper CrI**	**P_{MCMC}**
**Fixed effects**												
Intercept	0.919	0.627	1.222	<0.001	4.919	3.936	5.950	<0.001	0.591	0.327	0.868	<0.001
UA	0.073	0.056	0.091	**<0.001**								
PC1	0.001	-0.020	0.022	0.917	-0.045	-0.128	0.040	0.302	0.004	-0.017	0.024	0.712
PC2	0.006	-0.026	0.036	0.733	-0.040	-0.166	0.083	0.539	0.006	-0.025	0.036	0.717
Beak carotenoid	0.051	-0.047	0.150	0.315	0.035	-0.380	0.450	0.873	-0.002	-0.104	0.103	0.985
Leg carotenoid	-0.059	-0.175	0.062	0.342	-0.168	-0.652	0.340	0.506	-0.060	-0.180	0.065	0.332
Body mass	-0.011	-0.111	0.095	0.832	-0.360	-0.810	0.053	0.101	0.148	0.044	0.249	**0.007**
Diet (omnivore)	-0.062	-0.162	0.038	0.231	-0.176	-0.602	0.258	0.418	0.062	-0.046	0.157	0.222
Diet (herbivore)	0.016	-0.102	0.131	0.782	-0.454	-0.987	0.063	0.095	0.157	0.032	0.276	**0.014**
Sex (male)	0.008	-0.039	0.056	0.718	0.043	-0.119	0.213	0.620	-0.011	-0.058	0.031	0.637
Sex (unknown)	-0.003	-0.094	0.079	0.945	-0.302	-0.606	0.010	0.056	-0.117	-0.199	-0.040	**0.003**
**Random effects**												
Phylogeny	0.008	0.002	0.016		0.183	0.035	0.406		0.010	0.003	0.021	
Species	0.006	0.002	0.010		0.172	0.066	0.287		0.006	0.002	0.011	
Year	0.070	0.014	0.164		0.193	0.031	0.472		0.045	0.010	0.106	

	**MDA**				**ROMs**			
	**Post. mean**	**Lower CrI**	**Upper CrI**	**P_{MCMC}**	**Post. mean**	**Lower CrI**	**Upper CrI**	**P_{MCMC}**
**Fixed effects**								
Intercept	2.229	1.775	2.668	<0.001	1.671	1.209	2.138	<0.001
PC1	-0.009	-0.037	0.018	0.502	0.035	-0.004	0.072	0.071
PC2	-0.021	-0.061	0.020	0.304	0.032	-0.023	0.086	0.254

Beak carotenoid	0.055	-0.088	0.189	0.436	-0.022	-0.206	0.157	0.823
Leg carotenoid	0.062	-0.108	0.226	0.465	0.107	-0.123	0.323	0.354
Body mass	-0.193	-0.339	-0.050	**0.010**	-0.204	-0.393	-0.024	**0.030**
Diet (omnivore)	-0.097	-0.241	0.038	0.178	0.154	-0.023	0.324	0.091
Diet (herbivore)	-0.200	-0.381	-0.028	**0.028**	0.077	-0.123	0.273	0.428
Sex (male)	-0.012	-0.068	0.044	0.661	0.004	-0.105	0.104	0.940
Sex (unknown)	-0.001	-0.100	0.099	1.000	0.125	-0.013	0.260	0.074
**Random effects**								
Phylogeny	0.028	0.008	0.055		0.019	0.004	0.041	
Species	0.013	0.004	0.021		0.010	0.003	0.019	
Year	0.161	0.033	0.376		0.073	0.003	0.210	

Table S4. Results of Markov chain Monte Carlo (MCMC) phylogenetic linear mixed models showing the relationship of three antioxidant markers (TAS, UA, tGSH) and two markers of lipid peroxidation (MDA, ROMs) with plumage melanin (PC1) and carotenoid (PC2) pigment content, diet, and body mass, performed on a subset of data containing species with a sample size N ≥ 3. The number of species excluded from each subset varied between 11 and 14 (thus, the number of analyzed species varied between 66 and 93). Model parameters (posterior mean with 95% credibility intervals, CrI) were estimated based on 10,000 iterations of each model. Fixed effects with PMCMC < 0.05 are highlighted in bold.

	TAS				UA				tGSH			
	**Post. mean**	**Lower CrI**	**Upper CrI**	**P_{MCMC}**	**Post. mean**	**Lower CrI**	**Upper CrI**	**P_{MCMC}**	**Post. mean**	**Lower CrI**	**Upper CrI**	**P_{MCMC}**
**Fixed effects**												
Intercept	0.914	0.624	1.208	<0.001	4.845	3.820	5.839	<0.001	0.620	0.340	0.903	<0.001
UA	0.076	0.058	0.093	**<0.001**								
PC1	-0.001	-0.020	0.019	0.927	-0.047	-0.128	0.038	0.272	0.002	-0.019	0.021	0.876
PC2	-0.002	-0.034	0.028	0.877	-0.030	-0.157	0.098	0.647	0.005	-0.026	0.036	0.731
Beak carotenoid	0.059	-0.036	0.149	0.203	-0.129	-0.533	0.261	0.523	0.027	-0.069	0.125	0.575
Leg carotenoid	-0.058	-0.175	0.051	0.316	0.032	-0.477	0.501	0.912	-0.029	-0.147	0.091	0.626
Body mass	-0.023	-0.130	0.076	0.636	-0.326	-0.748	0.123	0.137	0.115	0.008	0.219	**0.034**
Diet (omnivore)	-0.028	-0.131	0.070	0.569	-0.221	-0.666	0.217	0.325	0.083	-0.019	0.186	0.111
Diet (herbivore)	0.039	-0.074	0.151	0.511	-0.557	-1.071	-0.030	**0.038**	0.167	0.043	0.293	**0.011**
Sex (male)	0.013	-0.033	0.059	0.581	0.068	-0.090	0.230	0.403	0.004	-0.038	0.048	0.833
Sex (unknown)	-0.009	-0.094	0.079	0.838	-0.184	-0.488	0.115	0.229	-0.096	-0.171	-0.018	**0.014**
**Random effects**												
Phylogeny	0.007	0.002	0.013		0.187	0.035	0.399		0.012	0.003	0.022	
Species	0.005	0.002	0.008		0.169	0.068	0.279		0.006	0.003	0.011	
Year	0.066	0.013	0.156		0.203	0.029	0.503		0.046	0.009	0.111	

	MDA				ROMs			
	**Post. mean**	**Lower CrI**	**Upper CrI**	**P_{MCMC}**	**Post. mean**	**Lower CrI**	**Upper CrI**	**P_{MCMC}**
**Fixed effects**								
Intercept	2.262	1.828	2.708	<0.001	1.667	1.203	2.138	<0.001
PC1	-0.009	-0.035	0.018	0.504	0.043	0.005	0.080	**0.027**
PC2	-0.023	-0.065	0.019	0.274	0.023	-0.032	0.076	0.385
Beak carotenoid	0.016	-0.122	0.140	0.809	0.005	-0.171	0.173	0.948
Leg carotenoid	0.095	-0.066	0.251	0.251	0.084	-0.127	0.301	0.444

Body mass	-0.198	-0.343	-0.050	**0.009**	-0.204	-0.398	-0.021	**0.036**
Diet (omnivore)	-0.105	-0.246	0.043	0.154	0.170	-0.005	0.359	0.067
Diet (herbivore)	-0.208	-0.380	-0.023	**0.024**	0.105	-0.100	0.298	0.300
Sex (male)	-0.017	-0.071	0.034	0.501	-0.010	-0.107	0.092	0.845
Sex (unknown)	-0.002	-0.104	0.094	0.967	0.077	-0.058	0.223	0.272
**Random effects**								
Phylogeny	0.028	0.008	0.053		0.018	0.004	0.039	
Species	0.015	0.006	0.024		0.010	0.003	0.019	
Year	0.152	0.026	0.355		0.070	0.003	0.206	

Table S5. Results of Markov chain Monte Carlo (MCMC) phylogenetic linear mixed models showing the relationship of three antioxidant markers (TAS, UA, tGSH) and two markers of lipid peroxidation (MDA, ROMs) with plumage melanin (PC1) and carotenoid (PC2) pigment content, diet, and body mass, performed on the full dataset, using an alternative phylogeny (Prum tree: Cooney et al 2017 Nature 542:344-347). Model parameters (posterior mean with 95% credibility intervals, CrI) were estimated based on 10,000 iterations of each model. Fixed effects with PMCMC < 0.05 are highlighted in bold.

	**TAS**				**UA**				**tGSH**			
	**Post. mean**	**Lower CrI**	**Upper CrI**	**P_{MCMC}**	**Post. mean**	**Lower CrI**	**Upper CrI**	**P_{MCMC}**	**Post. mean**	**Lower CrI**	**Upper CrI**	**P_{MCMC}**
**Fixed effects**												
Intercept	0.892	0.604	1.187	<0.001	4.866	3.904	5.834	<0.001	0.608	0.325	0.883	<0.001
UA	0.074	0.056	0.090	**<0.001**								
PC1	0.001	-0.018	0.021	0.908	-0.044	-0.127	0.035	0.284	0.004	-0.016	0.025	0.688
PC2	0.003	-0.027	0.031	0.825	-0.040	-0.160	0.079	0.513	0.002	-0.027	0.032	0.872
Beak carotenoid	0.051	-0.041	0.139	0.274	-0.129	-0.515	0.271	0.527	0.003	-0.095	0.099	0.944
Leg carotenoid	-0.061	-0.174	0.056	0.294	0.004	-0.479	0.487	0.995	-0.025	-0.145	0.094	0.687
Body mass	-0.004	-0.103	0.094	0.928	-0.347	-0.778	0.079	0.108	0.130	0.027	0.235	**0.015**
Diet (omnivore)	-0.059	-0.153	0.040	0.236	-0.194	-0.614	0.251	0.376	0.073	-0.023	0.178	0.155
Diet (herbivore)	0.024	-0.089	0.138	0.668	-0.494	-1.015	0.016	0.064	0.161	0.039	0.286	**0.014**
Sex (male)	0.009	-0.038	0.054	0.699	0.074	-0.085	0.227	0.355	-0.001	-0.043	0.042	0.956
Sex (unknown)	0.007	-0.078	0.095	0.868	-0.241	-0.532	0.068	0.118	-0.098	-0.171	-0.017	**0.013**
**Random effects**												
Phylogeny	0.007	0.002	0.014		0.177	0.035	0.384		0.013	0.004	0.024	
Species	0.005	0.002	0.008		0.181	0.076	0.296		0.006	0.002	0.010	
Year	0.069	0.014	0.162		0.195	0.031	0.480		0.045	0.009	0.106	

	**MDA**				**ROMs**			
	**Post. mean**	**Lower CrI**	**Upper CrI**	**P_{MCMC}**	**Post. mean**	**Lower CrI**	**Upper CrI**	**P_{MCMC}**
**Fixed effects**								
Intercept	2.239	1.818	2.687	<0.001	1.674	1.211	2.134	0.001
PC1	-0.010	-0.037	0.016	0.449	0.041	0.004	0.077	**0.029**
PC2	-0.014	-0.051	0.025	0.460	0.032	-0.019	0.085	0.228
Beak carotenoid	-0.001	-0.129	0.130	0.994	0.005	-0.163	0.172	0.954
Leg carotenoid	0.080	-0.083	0.243	0.323	0.074	-0.143	0.277	0.478
Body mass	-0.184	-0.326	-0.041	**0.010**	-0.202	-0.386	-0.034	**0.024**

Diet (omnivore)	-0.107	-0.252	0.033	0.143	0.158	-0.008	0.332	0.067
Diet (herbivore)	-0.209	-0.382	-0.032	**0.024**	0.087	-0.103	0.281	0.371
Sex (male)	-0.015	-0.067	0.035	0.569	-0.007	-0.100	0.093	0.892
Sex (unknown)	0.006	-0.091	0.107	0.921	0.105	-0.032	0.236	0.125
**Random effects**								
Phylogeny	0.027	0.008	0.052		0.018	0.004	0.037	
Species	0.014	0.006	0.024		0.010	0.003	0.018	
Year	0.153	0.032	0.354		0.075	0.003	0.227	

Table S6. Results of Markov chain Monte Carlo (MCMC) phylogenetic linear mixed models showing relationship between plumage eumelanin and pheomelanin scores and plasma glutathione (tGSH) levels. Model parameters (posterior mean with 95% credibility intervals, CrI) were estimated based on 10,000 iterations of each model. Fixed effects with PMCMC < 0.05 are highlighted in bold.

	**Eumelanin score**				**Pheomelanin score**			
	**Post. mean**	**Lower CrI**	**Upper CrI**	**P_{MCMC}**	**Post. mean**	**Lower CrI**	**Upper CrI**	**P_{MCMC}**
**Fixed terms**								
Intercept	0.604	0.320	0.873	<0.001	0.622	0.345	0.909	<0.001
Eumelanin	0.002	-0.017	0.021	0.870				
Pheomelanin					-0.005	-0.020	0.012	0.562
Beak carotenoid	0.003	-0.095	0.095	0.943	0.003	-0.094	0.096	0.943
Leg carotenoid	-0.022	-0.140	0.096	0.710	-0.020	-0.139	0.096	0.728
Body mass	0.125	0.022	0.226	**0.022**	0.125	0.018	0.217	**0.018**
Diet (omnivore)	0.078	-0.016	0.179	0.114	0.077	-0.021	0.174	0.120
Diet (herbivore)	0.160	0.039	0.285	**0.011**	0.158	0.035	0.282	**0.012**
Sex (male)	0.000	-0.043	0.042	0.996	-0.001	-0.044	0.040	0.942
Sex (unknown)	-0.097	-0.173	-0.020	**0.012**	-0.097	-0.174	-0.021	**0.013**
**Random terms**								
Phylogeny	0.012	0.003	0.023		0.012	0.003	0.023	
Species	0.006	0.002	0.010		0.006	0.002	0.010	
Year	0.045	0.009	0.106		0.045	0.010	0.107	

Table S7. Results of Markov chain Monte Carlo (MCMC) phylogenetic linear mixed models showing the relationship between plumage eumelanin and pheomelanin scores and plasma glutathione (tGSH) and reactive oxygen metabolite (ROMs) levels, performed on a subset of data from which data of 8 species (common kingfisher *Alcedo atthis*, Eurasian roller *Coracias garrulus*, red-rumped swallow *Hirundo daurica*, barn swallow *Hirundo rustica*, blue tit *Parus caeruleus*, magpie *Pica pica*, Eurasian starling *Sturnus vulgaris*, and rosy starling *Sturnus roseus*) with iridescent plumage were excluded. Model parameters (posterior mean with 95% credibility intervals, CrI) were estimated based on 10,000 iterations of each model. Fixed effects with PMCMC < 0.05 are highlighted in bold.

	**tGSH**								**ROMs**							
	**Eumelanin score**				**Pheomelanin score**				**Eumelanin score**				**Pheomelanin score**			
	**Post. mean**	**Lower CrI**	**Upper CrI**	P_{MCMC}	**Post. mean**	**Lower CrI**	**Upper CrI**	P_{MCMC}	**Post. mean**	**Lower CrI**	**Upper CrI**	P_{MCMC}	**Post. mean**	**Lower CrI**	**Upper CrI**	P_{MCMC}
**Fixed terms**																
Intercept	0.591	0.314	0.869	<0.001	0.608	0.332	0.900	<0.001	1.672	1.184	2.138	<0.001	1.750	1.267	2.231	<0.001
Eumelanin	0.001	-0.019	0.020	0.944					0.031	-0.006	0.069	0.106				
Pheomelanin					-0.004	-0.021	0.012	0.589					-0.025	-0.054	0.006	0.105
Beak carotenoid	-0.004	-0.107	0.099	0.958	-0.002	-0.106	0.099	0.980	-0.031	-0.214	0.152	0.753	-0.030	-0.212	0.154	0.759
Leg carotenoid	-0.059	-0.180	0.063	0.332	-0.058	-0.179	0.064	0.340	0.119	-0.101	0.353	0.290	0.114	-0.108	0.344	0.311
Body mass	0.147	0.044	0.251	**0.007**	0.146	0.043	0.247	**0.006**	-0.244	-0.430	-0.058	**0.010**	-0.215	-0.395	-0.032	**0.019**
Diet (omnivore)	0.067	-0.028	0.167	0.183	0.066	-0.031	0.164	0.192	0.196	0.023	0.372	**0.028**	0.180	0.003	0.347	**0.039**
Diet (herbivore)	0.159	0.040	0.285	**0.012**	0.157	0.037	0.282	**0.014**	0.108	-0.085	0.301	0.273	0.105	-0.086	0.300	0.285
Sex (male)	-0.008	-0.051	0.038	0.712	-0.011	-0.055	0.033	0.638	0.008	-0.098	0.113	0.885	0.015	-0.088	0.118	0.779
Sex (unknown)	-0.118	-0.195	-0.039	**0.003**	-0.118	-0.195	-0.038	**0.003**	0.124	-0.008	0.268	0.077	0.127	-0.009	0.265	0.071
**Random terms**																
Phylogeny	0.011	0.003	0.020		0.011	0.003	0.020		0.020	0.004	0.043		0.020	0.004	0.043	
Species	0.006	0.002	0.011		0.006	0.003	0.011		0.010	0.003	0.020		0.010	0.003	0.019	
Year	0.045	0.010	0.109		0.045	0.009	0.109		0.080	0.003	0.221		0.080	0.003	0.219	

Table S8. Results of Markov chain Monte Carlo (MCMC) phylogenetic linear mixed models showing the relationship between plumage eumelanin and pheomelanin scores and plasma glutathione (tGSH) and reactive oxygen metabolite (ROMs) levels, performed on a subset of data containing species with a sample size N ≥ 3. The number of species excluded from each subset varied between 11 and 14 (thus, the number of analyzed species varied between 66 and 93). Model parameters (posterior mean with 95% credibility intervals, CrI) were estimated based on 10,000 iterations of each model. Fixed effects with PMCMC < 0.05 are highlighted in bold.

	**tGSH**								**ROMs**							
	**Eumelanin score**				**Pheomelanin score**				**Eumelanin score**				**Pheomelanin score**			
	**Post. mean**	**Lower CrI**	**Upper CrI**	P_{MCMC}	**Post. mean**	**Lower CrI**	**Upper CrI**	P_{MCMC}	**Post. mean**	**Lower CrI**	**Upper CrI**	P_{MCMC}	**Post. mean**	**Lower CrI**	**Upper CrI**	P_{MCMC}
**Fixed terms**																
Intercept	0.619	0.349	0.898	<0.001	0.631	0.363	0.924	<0.001	1.670	1.198	2.134	<0.001	1.755	1.272	2.225	<0.001
Eumelanin	-0.001	-0.021	0.017	0.912					0.039	0.003	0.076	**0.037**				
Pheomelanin					-0.003	-0.019	0.014	0.719					-0.030	-0.060	0.000	**0.048**
Beak carotenoid	0.026	-0.069	0.123	0.601	0.026	-0.071	0.121	0.598	-0.004	-0.181	0.168	0.956	0.001	-0.177	0.171	1.000
Leg carotenoid	-0.027	-0.148	0.092	0.652	-0.025	-0.143	0.096	0.680	0.100	-0.112	0.322	0.369	0.084	-0.136	0.297	0.454
Body mass	0.114	0.002	0.212	**0.033**	0.112	0.009	0.214	**0.033**	-0.253	-0.444	-0.060	**0.010**	-0.213	-0.404	-0.029	**0.026**
Diet (omnivore)	0.087	-0.012	0.190	0.091	0.087	-0.014	0.187	0.092	0.209	0.023	0.387	**0.024**	0.190	0.010	0.370	**0.037**
Diet (herbivore)	0.169	0.048	0.293	**0.008**	0.167	0.044	0.288	**0.010**	0.134	-0.066	0.331	0.188	0.134	-0.069	0.328	0.188
Sex (male)	0.006	-0.036	0.048	0.772	0.004	-0.038	0.046	0.839	-0.007	-0.109	0.091	0.901	0.003	-0.096	0.101	0.962
Sex (unknown)	-0.096	-0.174	-0.019	**0.017**	-0.096	-0.170	-0.015	**0.017**	0.073	-0.068	0.211	0.301	0.078	-0.067	0.212	0.277
**Random terms**																
Phylogeny	0.012	0.003	0.022		0.012	0.003	0.022		0.019	0.004	0.041		0.019	0.003	0.040	
Species	0.006	0.002	0.011		0.006	0.002	0.011		0.010	0.003	0.020		0.010	0.003	0.020	
Year	0.045	0.009	0.107		0.045	0.009	0.107		0.072	0.003	0.223		0.072	0.003	0.222	

Table S9. Results of Markov chain Monte Carlo (MCMC) phylogenetic linear mixed models showing the relationship between plumage eumelanin and pheomelanin score and plasma glutathione (tGSH) and reactive oxygen metabolite (ROMs) levels, performed on the full dataset, using an alternative phylogeny (Prum tree: Cooney et al 2017 Nature 542:344-347). Model parameters (posterior mean with 95% credibility intervals, CrI) were estimated based on 10,000 iterations of each model. Fixed effects with PMCMC < 0.05 are highlighted in bold.

	**tGSH**								**ROMs**							
	**Eumelanin score**				**Pheomelanin score**				**Eumelanin score**				**Pheomelanin score**			
	**Post. mean**	**Lower CrI**	**Upper CrI**	P_{MCMC}	**Post. mean**	**Lower CrI**	**Upper CrI**	P_{MCMC}	**Post. mean**	**Lower CrI**	**Upper CrI**	P_{MCMC}	**Post. mean**	**Lower CrI**	**Upper CrI**	P_{MCMC}
**Fixed terms**																
Intercept	0.605	0.320	0.872	<0.001	0.624	0.339	0.900	<0.001	1.675	1.214	2.147	<0.001	1.754	1.274	2.222	<0.001
Eumelanin	0.002	-0.019	0.020	0.860					0.035	-0.001	0.069	0.053				
Pheomelanin					-0.005	-0.021	0.011	0.552					-0.028	-0.056	0.000	0.051
Beak carotenoid	0.003	-0.094	0.098	0.961	0.003	-0.093	0.098	0.961	-0.008	-0.173	0.169	0.929	-0.003	-0.168	0.173	0.975
Leg carotenoid	-0.025	-0.147	0.089	0.680	-0.023	-0.137	0.098	0.706	0.087	-0.122	0.304	0.423	0.075	-0.140	0.285	0.484
Body mass	0.128	0.023	0.233	**0.017**	0.128	0.026	0.228	**0.015**	-0.246	-0.419	-0.062	**0.010**	-0.210	-0.382	-0.038	**0.019**
Diet (omnivore)	0.076	-0.023	0.173	0.124	0.075	-0.029	0.167	0.132	0.194	0.023	0.366	**0.029**	0.178	0.003	0.341	**0.041**
Diet (herbivore)	0.164	0.045	0.292	**0.010**	0.162	0.045	0.294	**0.011**	0.116	-0.071	0.311	0.229	0.116	-0.068	0.313	0.230
Sex (male)	0.000	-0.042	0.044	0.998	-0.002	-0.045	0.040	0.950	0.001	-0.098	0.092	0.991	0.007	-0.085	0.102	0.884
Sex (unknown)	-0.098	-0.178	-0.024	**0.015**	-0.098	-0.176	-0.022	**0.016**	0.104	-0.021	0.243	0.120	0.107	-0.021	0.243	0.107
**Random terms**																
Phylogeny	0.013	0.004	0.025		0.013	0.003	0.024		0.019	0.004	0.041		0.019	0.004	0.041	
Species	0.006	0.002	0.010		0.006	0.002	0.010		0.010	0.003	0.019		0.010	0.003	0.019	
Year	0.045	0.009	0.106		0.045	0.008	0.105		0.070	0.003	0.213		0.070	0.003	0.211	

References

Bókony V, Lendvai ÁZ, Vágási CI, Pătraş L, Pap PL, Németh J, Vincze E, Papp S, Preiszner B, Seress G, Liker A (2014) Necessity or capacity? Physiological state predicts problem-solving performance in house sparrows. Behavioral Ecology 25(1):124–135. DOI: 10.1093/beheco/art094

Cohen AA, McGraw KJ, Wiersma P, Williams JB, Robinson WD, Robinson TR, Brawn JD, Ricklefs RE (2008) Interspecific associations between circulating antioxidant levels and life-history variation in birds. The American Naturalist 172(2):178–193. DOI: 10.1086/589456

Cooney C, Bright J, Capp E, Chira AM, Hughes EC, Moody CJA, Nouri LO, Varley ZK, Thomaset GH (2017) Mega-evolutionary dynamics of the adaptive radiation of birds. Nature 542:344–347. DOI: 10.1038/nature21074

Erel O (2004) A novel automated direct measurement method for total antioxidant capacity using a new generation, more stable ABTS radical cation. Clinical Biochemistry 37(4):277–285. DOI: 10.1016/j.clinbiochem.2003.11.015

Galván I, Alonso-Alvarez C (2008) An intracellular antioxidant determines the expression of a melanin-based signal in a bird. PLOS ONE 3(10):e3335. DOI: 10.1371/journal.pone.0003335

Hõrak P, Sild E, Soomets U, Sepp T, Kilk K (2010) Oxidative stress and information content of black and yellow plumage coloration: an experiment with greenfinches. Journal of Experimental Biology 213(13):2225–2233. DOI: 10.1242/jeb.042085

Noguera JC, Lores M, Alonso-Álvarez C, Velando A (2011) Thrifty development: early-life diet restriction reduces oxidative damage during later growth. Functional Ecology 25(5):1144–1153. DOI: 10.1111/j.1365-2435.2011.01856.x

Sepp T, Sild E, Hõrak P (2010) Hematological condition indexes in greenfinches: effects of captivity and diurnal variation. Physiological and Biochemical Zoology 83(2):276–282. DOI: 10.1086/648580

Interspecific associations between oxidative physiology and pigmentation in birds

Attila Marton, Csongor I. Vágási, Orsolya Vincze, Veronika Bókony, Péter L. Pap, Laura Pătraş, Janka Pénzes, Lőrinc Bărbos, Attila Fülöp, Gergely Osváth, Simon Ducatez, Mathieu Giraudeau

01-03-2022

Variable descriptions

ring: individual ring number

species: scientific names, used in the analyses for defining the “species” random effect because multiple individuals per species were sampled

animal: scientific names (same as in “species”), this duplicate column is used in the analyses for matching the dataset with the phylogeny as per MCMCglmm syntax

year: year of sampling, to control for year-effect

date: date of sampling

sex: sex based on morphological characteristics and/or molecular sexing

TAS: total antioxidant status

UA: uric acid

MDA: malondialdehyde

tGSH: total gluthatione

ROM: reactive oxygen metabolites

iridescence: logical variable describing the presence/absence of iridescence of plumage

eumelanin: eumelanin plumage score (0-5)

pheomelanin: pheomelanin plumage score (0-5)

carotenoid: carotenoid plumage score (0-5)

carotBeak: logical variable describing the presence of carotenoids in beak and cere

carotLegs: logical variable describing the presence of carotenoids in legs

PC1: first principal component values (describing melanin-based coloration)

PC2: second principal component values (describing carotenoid-based coloration)

diet: species-specific diet based on Storchová and Hořák (Global Ecology and Biogeography, 274(4):400, 2018), but with categories collapsed to three levels: omnivore, plan-based diet, and carnivorous diet

mass: sex and species-specific mass from Storchová and Hořák (Global Ecology and Biogeography, 274(4):400, 2018), with some corrections

Data preparation

```
R.Version()

## $platform
## [1] "x86_64-w64-mingw32"
##
## $arch
## [1] "x86_64"
##
## $os
## [1] "mingw32"
##
## $system
## [1] "x86_64, mingw32"
##
## $status
## [1] ""
##
## $major
## [1] "4"
##
## $minor
## [1] "1.1"
##
## $year
## [1] "2021"
##
## $month
## [1] "08"
##
## $day
## [1] "10"
##
## $`svn rev`
## [1] "80725"
##
## $language
## [1] "R"
##
## $version.string
```

```
## [1] "R version 4.1.1 (2021-08-10)"
##
## $nickname
## [1] "Kick Things"
```

```
starttime <- Sys.time()
```

```
setwd("C:/Users/Marton Attila/OneDrive - Debreceni Egyetem/PhD/Science/2021-antioxidant vs pigmentation/ms/data")
```

```
library(ape)
library(phytools)
```

```
## Loading required package: maps
```

```
library(MCMCglmm)
```

```
## Loading required package: Matrix
```

```
## Loading required package: coda
```

```
library(car)
```

```
## Loading required package: carData
```

```
library(ggplot2)
library(tidyr)
```

```
##
## Attaching package: 'tidyr'
```

```
## The following objects are masked from 'package:Matrix':
##
##     expand, pack, unpack
```

```
library(dplyr)
```

```
##
## Attaching package: 'dplyr'
```

```
## The following object is masked from 'package:car':
##
##     recode
```

```
## The following objects are masked from 'package:stats':
##
##     filter, lag
```

```
## The following objects are masked from 'package:base':
##
##     intersect, setdiff, setequal, union
```

```
library(stringr)
library(lme4)
library(gtools)

## Warning: package 'gtools' was built under R version 4.1.2

##
## Attaching package: 'gtools'

## The following object is masked from 'package:car':
##
##     logit

library(stringi)
library(merTools)

## Warning: package 'merTools' was built under R version 4.1.2

## Loading required package: arm

## Warning: package 'arm' was built under R version 4.1.2

## Loading required package: MASS

##
## Attaching package: 'MASS'

## The following object is masked from 'package:dplyr':
##
##     select

##
## arm (Version 1.12-2, built: 2021-10-15)

## Working directory is C:/Users/Marton Attila/OneDrive - Debreceni
Egyetem/PhD/Science/2021-antioxidant vs pigmentation/ms/data

##
## Attaching package: 'arm'

## The following object is masked from 'package:gtools':
##
##     logit

## The following object is masked from 'package:car':
##
##     logit

## The following object is masked from 'package:coda':
##
##     traceplot
```

```
## The following object is masked from 'package:ape':
##
##     balance

library(emmeans)
library(rptR)

## Warning: package 'rptR' was built under R version 4.1.2

# reading consensus tree (Jetz) and dataset with 104 species:

tree = read.nexus("consensus_104_ultametric.nex")

data = read.csv("OS_pigmentation_data.csv", header = T, sep = ";",
stringsAsFactors = TRUE)

str(data)

## 'data.frame':    1387 obs. of  23 variables:
##  $ no          : int  1 2 3 4 5 6 7 8 9 10 ...
##  $ ring        : Factor w/ 1377 levels "3x07253","3x08482",..: 55 954 956
1088 1089 1131 1100 955 188 1016 ...
##  $ species     : Factor w/ 104 levels "Accipiter_nisus",..: 1 2 2 2 2 2 2
2 2 2 ...
##  $ animal      : Factor w/ 104 levels "Accipiter_nisus",..: 1 2 2 2 2 2 2
2 2 2 ...
##  $ year        : int  2017 2012 2012 2019 2019 2019 2019 2012 2016 2016
...
##  $ date        : Factor w/ 196 levels "01/05/2011","01/05/2012",..: 111 5
5 145 196 138 153 5 140 64 ...
##  $ sex         : Factor w/ 3 levels "female","male",..: 2 1 1 1 1 1 1 2 2
2 ...
##  $ TAS         : num  0.451 2.653 2.145 2.204 1.309 ...
##  $ UA          : num  6.89 32.41 16.72 34.94 21.15 ...
##  $ MDA         : num  0.77 5.89 3.96 4.2 3.37 ...
##  $ tGSH        : num  3.903 8.333 5 NA 0.545 ...
##  $ ROM         : num  NA 3.816 0.593 NA NA ...
##  $ irridescence: int  0 0 0 0 0 0 0 0 0 0 ...
##  $ eumelanin   : int  2 0 0 0 0 0 0 0 0 0 ...
##  $ pheomelanin : int  2 5 5 5 5 5 5 5 5 5 ...
##  $ carotenoid  : int  0 0 0 0 0 0 0 0 0 0 ...
##  $ carotBeak   : int  1 0 0 0 0 0 0 0 0 0 ...
##  $ carotLegs   : int  1 0 0 0 0 0 0 0 0 0 ...
##  $ PC1         : num  0.406 -1.717 -1.717 -1.717 -1.717 ...
##  $ PC2         : num  -0.5018 0.0112 0.0112 0.0112 0.0112 ...
##  $ diet        : Factor w/ 3 levels "animals","omni",..: 1 1 1 1 1 1 1 1 1
1 ...
##  $ mass        : num  144 28.3 28.3 28.3 28.3 28.3 28.3 32.3 32.3 32.3 ...
##  $ massSource  : Factor w/ 6 levels "Calero-Riestra M, García JT (2016)
Sex-dependent differences in avian malaria prevalence and consequences of
in"| __truncated__,..: 5 5 5 5 5 5 5 5 5 5 ...
```

```
data$irridescence = as.factor(data$irridescence)

data$carotBeak = as.factor(data$carotBeak)

data$carotLegs = as.factor(data$carotLegs)

data$year = as.factor(data$year)

# transforming the variables to improve model fit (based on diagnostic plots.
We chose the transformation that provides the best diagnostics for each
response variable):

data$TAS2 = sqrt(data$TAS)

data$UA2 = sqrt(data$UA)

data$MDA2 = sqrt(data$MDA)

#data$tGSH2 = sqrt(data$tGSH) #sqrt did not solve heterogeneity here, so
using log:

data$log10tGSH = log10(data$tGSH + 1)

data$ROM2 = sqrt(data$ROM)

data$log10mass = log10(data$mass)

# creating subsets with no missing value for each dependent variable:

data.tas = data[!is.na(data$TAS),]

data.ua = data[!is.na(data$UA),]

data.tas.ua = data[!is.na(data$TAS) & !is.na(data$UA),]

data.mda = data[!is.na(data$MDA),]

data.tgsh = data[!is.na(data$tGSH),]

data.rom = data[!is.na(data$ROM),]
```

Species-specific repeatability testing

```
#d <- read.csv("2021data_corrected.csv", sep=';')
#test <- c('TAS','UA','MDA','tGSH','ROM')

d <- data
```

```
test <- c('TAS2','UA2','MDA2','log10tGSH','ROM2')

d$ID <- paste(d$species,d$ring, d$date, sep='/') # ID is created to count the
number of individuals whose data is used here

ID <- c()

REP <- as.data.frame(array(,c(length(test), 11)))

names(REP) <- c("test", "nosp", "nyear", "Rsp","Ryear", "pSp", "pYear",
"LCIsp", "LCIyear", "UCIsp", "UCIyear")

for(j in 1:length(test)){
  i <- test[j]
  REP[j,1] <- i
  rep <- rpt(as.formula(paste(i, " ~  (1|species) +(1|year)")),
             grname = c("species","year"),
             data = d[!is.na(d[,i]) & !is.infinite(d[,i]),], datatype =
"Gaussian",
             nboot = 1000, npermut = 0)

# ID <- c(ID, d$ID[d$species%in%sp & !is.na(d[,i])])
  print(rep)
  REP[j,2:3] <- rep$ngroups
  REP[j,4:5] <- rep$R
  REP[j,6:7] <- rep$P[,1]
  REP[j,8:9] <- rep$CI_emp[,1]
  REP[j,10:11] <- rep$CI_emp[,2]
}

# species-specific repeatability

REP[,4:11] <- round(REP[,4:11],4)

print(REP)

##        test nosp nyear    Rsp  Ryear    pSp  pYear  LCIsp LCIyear  UCIsp
## 1      TAS2  104     7 0.0290 0.2891 0.0017 0.0000 0.0058  0.0763 0.0660
## 2       UA2  104     7 0.1650 0.0426 0.0000 0.0145 0.1069  0.0016 0.2299
## 3      MDA2  104     7 0.0991 0.3877 0.0000 0.0000 0.0547  0.1031 0.1672
## 4 log10tGSH  104     7 0.1191 0.1978 0.0000 0.0000 0.0688  0.0426 0.1747
## 5      ROM2   79     3 0.0625 0.0490 0.0175 0.0310 0.0000  0.0000 0.1358
##   UCIyear
## 1  0.4890
## 2  0.1073
## 3  0.5945
## 4  0.3927
## 5  0.1711
```

Prior definition

We defined priors for each dependent variable, splitting its variance equally between the 3 random effects and the residuals:

```
p.var.tas <- var(data.tas$TAS2,na.rm = TRUE)

prior.tas <- list(G = list(G1 = list(V = matrix(p.var.tas/4),n = 1), G2 =
list(V = matrix(p.var.tas/4),n = 1), G3 = list(V = matrix(p.var.tas/4),n =
1)), R = list(V = matrix(p.var.tas/4),n = 1))

p.var.ua <- var(data.ua$UA2,na.rm = TRUE)

prior.ua <- list(G = list(G1 = list(V = matrix(p.var.ua/4),n = 1), G2 =
list(V = matrix(p.var.ua/4),n = 1), G3 = list(V = matrix(p.var.ua/4),n = 1)),
R = list(V = matrix(p.var.ua/4),n = 1))

p.var.tas.ua <- var(data.tas.ua$TAS2,na.rm = TRUE)

prior.tas.ua <- list(G = list(G1 = list(V = matrix(p.var.tas.ua/4),n = 1), G2
= list(V = matrix(p.var.tas.ua/4),n = 1), G3 = list(V =
matrix(p.var.tas.ua/4),n = 1)), R = list(V = matrix(p.var.tas.ua/4),n = 1))

p.var.mda <- var(data.mda$MDA2,na.rm = TRUE)

prior.mda <- list(G = list(G1 = list(V = matrix(p.var.mda/4),n = 1), G2 =
list(V = matrix(p.var.mda/4),n = 1), G3 = list(V = matrix(p.var.mda/4),n =
1)), R = list(V = matrix(p.var.mda/4),n = 1))

p.var.tgsh <- var(data.tgsh$log10tGSH,na.rm = TRUE)

prior.tgsh <- list(G = list(G1 = list(V = matrix(p.var.tgsh/4),n = 1), G2 =
list(V = matrix(p.var.tgsh/4),n = 1), G3 = list(V = matrix(p.var.tgsh/4),n =
1)), R = list(V = matrix(p.var.tgsh/4),n = 1))

p.var.rom <- var(data.rom$ROM2,na.rm = TRUE)

prior.rom <- list(G = list(G1 = list(V = matrix(p.var.rom/4),n = 1), G2 =
list(V = matrix(p.var.rom/4),n = 1), G3 = list(V = matrix(p.var.rom/4),n =
1)), R = list(V = matrix(p.var.rom/4),n = 1))
```

Phylogenetic s signal testing

For each response variable, we estimate phylogenetic signal (λ) in two alternative ways. First, we take λ as the proportion of variance explained by the phylogeny out of all variance in the data. This approach treats within-species variance as a biologically relevant, species-

specific trait. In the second version, we exclude the within-species variance, treating it as measurement error (noise):

```
lambdamodel.tas=MCMCglmm(TAS2 ~ 1, random=~animal+species+year,
pedigree=tree,
prior=prior.tas, data=data.tas, nitt=250001, thin=225, burnin=25000)

lambdamodel.ua=MCMCglmm(UA2 ~ 1, random=~animal+species+year, pedigree=tree,
prior=prior.ua, data=data.ua, nitt=250001, thin=225, burnin=25000)

lambdamodel.mda=MCMCglmm(MDA2 ~ 1, random=~animal+species+year,
pedigree=tree,
prior=prior.mda, data=data.mda, nitt=250001, thin=225, burnin=25000)

lambdamodel.tgsh=MCMCglmm(log10tGSH ~ 1, random=~animal+species+year,
pedigree=tree,
prior=prior.tgsh, data=data.tgsh, nitt=250001, thin=225, burnin=25000)

lambdamodel.rom=MCMCglmm(ROM2 ~ 1, random=~animal+species+year,
pedigree=tree,
prior=prior.rom, data=data.rom, nitt=250001, thin=225, burnin=25000)



lambda.tas.1 = lambdamodel.tas$VCV[,'animal'] /
(lambdamodel.tas$VCV[,'animal'] +
lambdamodel.tas$VCV[,'species'] + lambdamodel.tas$VCV[,'year'] +
lambdamodel.tas$VCV[,'units'])

posterior.mode(lambda.tas.1)

##       var1
## 0.03589235

HPDinterval(lambda.tas.1)

##            lower      upper
## var1 0.01320427 0.08068133
## attr(,"Probability")
## [1] 0.95005

lambda.tas.2 = lambdamodel.tas$VCV[,'animal'] /
(lambdamodel.tas$VCV[,'animal'] +
lambdamodel.tas$VCV[,'species'] + lambdamodel.tas$VCV[,'year'])

posterior.mode(lambda.tas.2)

##      var1
## 0.0868851

HPDinterval(lambda.tas.2)
```

```
##            lower     upper
## var1 0.01877047 0.2614061
## attr(,"Probability")
## [1] 0.95005
```

```
lambda.ua.1 = lambdamodel.ua$VCV[,'animal'] / (lambdamodel.ua$VCV[,'animal']
+
lambdamodel.ua$VCV[,'species'] + lambdamodel.ua$VCV[,'year'] +
lambdamodel.ua$VCV[,'units'])

posterior.mode(lambda.ua.1)
```

```
##      var1
## 0.1076937
```

```
HPDinterval(lambda.ua.1)
```

```
##           lower     upper
## var1 0.0267491 0.2257958
## attr(,"Probability")
## [1] 0.95005
```

```
lambda.ua.2 = lambdamodel.ua$VCV[,'animal'] / (lambdamodel.ua$VCV[,'animal']
+
lambdamodel.ua$VCV[,'species'] + lambdamodel.ua$VCV[,'year'])

posterior.mode(lambda.ua.2)
```

```
##     var1
## 0.306308
```

```
HPDinterval(lambda.ua.2)
```

```
##           lower     upper
## var1 0.1319797 0.6811811
## attr(,"Probability")
## [1] 0.95005
```

```
lambda.mda.1 = lambdamodel.mda$VCV[,'animal'] /
(lambdamodel.mda$VCV[,'animal'] +
lambdamodel.mda$VCV[,'species'] + lambdamodel.mda$VCV[,'year'] +
lambdamodel.mda$VCV[,'units'])

posterior.mode(lambda.mda.1)
```

```
##       var1
## 0.09142686
```

```
HPDinterval(lambda.mda.1)
```

```
##            lower     upper
## var1 0.02361939 0.2060498
```

```
## attr(,"Probability")
## [1] 0.95005
```

```
lambda.mda.2 = lambdamodel.mda$VCV[,'animal'] /
(lambdamodel.mda$VCV[,'animal'] +
lambdamodel.mda$VCV[,'species'] + lambdamodel.mda$VCV[,'year'])

posterior.mode(lambda.mda.2)
```

```
##       var1
## 0.09764209
```

```
HPDinterval(lambda.mda.2)
```

```
##           lower     upper
## var1 0.03106485 0.4255724
## attr(,"Probability")
## [1] 0.95005
```

```
lambda.tgsh.1 = lambdamodel.tgsh$VCV[,'animal'] /
(lambdamodel.tgsh$VCV[,'animal'] +
lambdamodel.tgsh$VCV[,'species'] + lambdamodel.tgsh$VCV[,'year'] +
lambdamodel.tgsh$VCV[,'units'])

posterior.mode(lambda.tgsh.1)
```

```
##      var1
## 0.1297387
```

```
HPDinterval(lambda.tgsh.1)
```

```
##           lower     upper
## var1 0.04563088 0.2051215
## attr(,"Probability")
## [1] 0.95005
```

```
lambda.tgsh.2 = lambdamodel.tgsh$VCV[,'animal'] /
(lambdamodel.tgsh$VCV[,'animal'] +
lambdamodel.tgsh$VCV[,'species'] + lambdamodel.tgsh$VCV[,'year'])

posterior.mode(lambda.tgsh.2)
```

```
##      var1
## 0.3304267
```

```
HPDinterval(lambda.tgsh.2)
```

```
##         lower     upper
## var1 0.102385 0.5686859
## attr(,"Probability")
## [1] 0.95005
```

```
lambda.rom.1 = lambdamodel.rom$VCV[,'animal'] /
(lambdamodel.rom$VCV[,'animal'] +
lambdamodel.rom$VCV[,'species'] + lambdamodel.rom$VCV[,'year'] +
lambdamodel.rom$VCV[,'units'])

posterior.mode(lambda.rom.1)

##       var1
## 0.05441251

HPDinterval(lambda.rom.1)

##            lower     upper
## var1 0.008140277 0.1703917
## attr(,"Probability")
## [1] 0.95005

lambda.rom.2 = lambdamodel.rom$VCV[,'animal'] /
(lambdamodel.rom$VCV[,'animal'] +
lambdamodel.rom$VCV[,'species'] + lambdamodel.rom$VCV[,'year'])

posterior.mode(lambda.rom.2)

##      var1
## 0.2657349

HPDinterval(lambda.rom.2)

##          lower     upper
## var1 0.0382381 0.6390429
## attr(,"Probability")
## [1] 0.95005

# creating two tables with the different approaches

lambda.vars <- c(test)

posterior.mode.1 <- c(posterior.mode(lambda.tas.1),
                      posterior.mode(lambda.ua.1),
                      posterior.mode(lambda.mda.1),
                      posterior.mode(lambda.tgsh.1),
                      posterior.mode(lambda.rom.1))

lower.HPDinterval.1 <- c(HPDinterval(lambda.tas.1)[1],
                      HPDinterval(lambda.ua.1)[1],
                      HPDinterval(lambda.mda.1)[1],
                      HPDinterval(lambda.tgsh.1)[1],
                      HPDinterval(lambda.rom.1)[1])

upper.HPDinterval.1 <- c(HPDinterval(lambda.tas.1)[2],
                      HPDinterval(lambda.ua.1)[2],
```

```
                      HPDinterval(lambda.mda.1)[2],
                      HPDinterval(lambda.tgsh.1)[2],
                      HPDinterval(lambda.rom.1)[2])

posterior.mode.2 <- c(posterior.mode(lambda.tas.2),
                      posterior.mode(lambda.ua.2),
                      posterior.mode(lambda.mda.2),
                      posterior.mode(lambda.tgsh.2),
                      posterior.mode(lambda.rom.2))

lower.HPDinterval.2 <- c(HPDinterval(lambda.tas.2)[1],
                      HPDinterval(lambda.ua.2)[1],
                      HPDinterval(lambda.mda.2)[1],
                      HPDinterval(lambda.tgsh.2)[1],
                      HPDinterval(lambda.rom.2)[1])

upper.HPDinterval.2 <- c(HPDinterval(lambda.tas.2)[2],
                      HPDinterval(lambda.ua.2)[2],
                      HPDinterval(lambda.mda.2)[2],
                      HPDinterval(lambda.tgsh.2)[2],
                      HPDinterval(lambda.rom.2)[2])

# phylogenetic signal with within-species variance as a biologically relevant

lambda.1 <- data.frame(lambda.vars, posterior.mode.1, lower.HPDinterval.1,
upper.HPDinterval.1)

# phylogenetic signal with within-species variance as a biologically relevant

lambda.2 <- data.frame(lambda.vars, posterior.mode.2, lower.HPDinterval.2,
upper.HPDinterval.2)

print(lambda.1)

##   lambda.vars posterior.mode.1 lower.HPDinterval.1 upper.HPDinterval.1
## 1        TAS2       0.03589235         0.013204272          0.08068133
## 2         UA2       0.10769374         0.026749100          0.22579584
## 3        MDA2       0.09142686         0.023619390          0.20604980
## 4   log10tGSH       0.12973873         0.045630876          0.20512152
## 5        ROM2       0.05441251         0.008140277          0.17039169

print(lambda.2)

##   lambda.vars posterior.mode.2 lower.HPDinterval.2 upper.HPDinterval.2
## 1        TAS2       0.08688510          0.01877047           0.2614061
## 2         UA2       0.30630796          0.13197967           0.6811811
## 3        MDA2       0.09764209          0.03106485           0.4255724
## 4   log10tGSH       0.33042674          0.10238501           0.5686859
## 5        ROM2       0.26573489          0.03823810           0.6390429
```

Information on sample sizes:

• All 104 species have data for all oxidative parameters, except that 25 species have no data for ROM.

• There are 8 species in the 104-species dataset for which the coloration scores may be affected by iridescence. One of these 8 species have no ROM data.

```
# Checking multi-collinearity - no problem! (all VIF < 2):

# in the full dataset:

m1 = lm(no ~ PC1 + PC2 + carotBeak + carotLegs + year + sex + log10(mass) +
diet, data = data)
v = vif(m1)
v[,3]^2

##         PC1         PC2   carotBeak   carotLegs        year         sex
##    1.158462    1.273858    1.556444    1.589929    1.039471    1.129041
## log10(mass)        diet
##    1.768259    1.188999

# in subsets for each response variable:

m2 = lm(TAS2 ~ UA2 + PC1 + PC2 + carotBeak + carotLegs + year + sex +
log10(mass) + diet, data = data.tas.ua)
v = vif(m2)
v[,3]^2

##         UA2         PC1         PC2   carotBeak   carotLegs        year
##    1.078190    1.126123    1.303258    1.598249    1.576698    1.041310
##         sex log10(mass)        diet
##    1.115543    1.827714    1.214316

m3 = lm(UA2 ~ PC1 + PC2 + carotBeak + carotLegs + year + sex + log10(mass) +
diet, data = data.ua)
v = vif(m3)
v[,3]^2

##         PC1         PC2   carotBeak   carotLegs        year         sex
##    1.131025    1.293331    1.544150    1.572337    1.042166    1.116358
## log10(mass)        diet
##    1.756356    1.194423

m4 = lm(MDA2 ~ PC1 + PC2 + carotBeak + carotLegs + year + sex + log10(mass) +
diet, data = data.mda)
v = vif(m4)
v[,3]^2

##         PC1         PC2   carotBeak   carotLegs        year         sex
##    1.135151    1.287756    1.532449    1.545690    1.040761    1.113155
```

```
## log10(mass)        diet
##    1.763344    1.193550

m5 = lm(log10tGSH ~ PC1 + PC2 + carotBeak + carotLegs + year + sex +
log10(mass) + diet, data = data.tgsh)
v = vif(m5)
v[,3]^2

##         PC1         PC2   carotBeak   carotLegs        year         sex
##    1.130485    1.247326    1.605637    1.544178    1.039342    1.115630
## log10(mass)        diet
##    1.729942    1.160771

m6 = lm(ROM2 ~ PC1 + PC2 + carotBeak + carotLegs + year + sex + log10(mass) +
diet, data = data.rom)
v = vif(m6)
v[,3]^2

##         PC1         PC2   carotBeak   carotLegs        year         sex
##    1.193230    1.289594    1.504248    1.733558    1.071368    1.168938
## log10(mass)        diet
##    1.814596    1.266089
```

Full MCMCglmm models

We did not z-transform the continuous fixed effects (i.e. body mass, as PC1-2 are already standardized) because model fit is good and the coefficients are easier to interpret without further transformation. The only significant relationship with coloration is a positive correlation of ROM~PC1:

TAS vs. PC1 + PC2, all data

```
set.seed(7777)

m.tas.ua = MCMCglmm(TAS2 ~ UA2 + PC1 + PC2 + carotBeak + carotLegs +
log10mass + diet + sex, random = ~animal + species + year, pedigree = tree,
prior = prior.tas.ua, data = data.tas.ua, nitt = 2500001, thin = 245, burnin
= 50000)

plot(m.tas.ua)

autocorr(m.tas.ua$VCV)

summary(m.tas.ua)

##
##  Iterations = 50001:2500001
##  Thinning interval  = 245
##  Sample size  = 10001
##
##  DIC: 662.3051
```

```
##
##  G-structure:  ~animal
##
##        post.mean l-95% CI u-95% CI eff.samp
## animal  0.007399 0.002392  0.01394    10001
##
##                ~species
##
##         post.mean l-95% CI u-95% CI eff.samp
## species  0.005065 0.002155 0.008549     8932
##
##                ~year
##
##      post.mean l-95% CI u-95% CI eff.samp
## year   0.06707  0.01271   0.1604    10500
##
##  R-structure:  ~units
##
##       post.mean l-95% CI u-95% CI eff.samp
## units    0.1057  0.09638   0.1151    10001
##
##  Location effects: TAS2 ~ UA2 + PC1 + PC2 + carotBeak + carotLegs + log10mass + diet + sex
##
##             post.mean  l-95% CI  u-95% CI eff.samp  pMCMC
## (Intercept)  0.900105  0.585085  1.179901    10001 <1e-04 ***
## UA2          0.073495  0.056765  0.090512    10001 <1e-04 ***
## PC1          0.001277 -0.018318  0.020326    10001  0.903
## PC2          0.003354 -0.024474  0.034797    10001  0.840
## carotBeak1   0.054522 -0.039716  0.140883    10001  0.242
## carotLegs1  -0.066695 -0.176913  0.046662    10001  0.244
## log10mass   -0.006611 -0.105404  0.090687    10001  0.894
## dietomni    -0.058240 -0.155452  0.040575    10001  0.248
## dietplants   0.027885 -0.085695  0.139637    10001  0.630
## sexmale      0.008972 -0.034727  0.055823    10001  0.701
## sexunknown   0.007246 -0.077859  0.092881    10001  0.861
## ---
## Signif. codes:  0 '***' 0.001 '**' 0.01 '*' 0.05 '.' 0.1 ' ' 1
```

UA vs. PC1 + PC2, all data

```
set.seed(7777)

m.ua = MCMCglmm(UA2 ~ PC1 + PC2 + carotBeak + carotLegs + log10mass + diet + sex, random = ~animal + species + year, pedigree = tree, prior = prior.ua, data = data.ua, nitt = 2500001, thin = 245, burnin = 50000)

plot(m.ua)

autocorr(m.ua$VCV)
```

```
summary(m.ua)

##
##  Iterations = 50001:2500001
##  Thinning interval  = 245
##  Sample size  = 10001
##
##  DIC: 3675.789
##
##  G-structure:  ~animal
##
##        post.mean l-95% CI u-95% CI eff.samp
## animal    0.1861  0.03037    0.403    10299
##
##                ~species
##
##         post.mean l-95% CI u-95% CI eff.samp
## species     0.175  0.06534   0.2866     9470
##
##                ~year
##
##      post.mean l-95% CI u-95% CI eff.samp
## year     0.197  0.02944   0.4959    10001
##
##  R-structure:  ~units
##
##       post.mean l-95% CI u-95% CI eff.samp
## units     1.363    1.245    1.477    10001
##
##  Location effects: UA2 ~ PC1 + PC2 + carotBeak + carotLegs + log10mass +
diet + sex
##
##             post.mean  l-95% CI  u-95% CI eff.samp  pMCMC
## (Intercept)  4.924261  3.926938  5.959784    10001 <1e-04 ***
## PC1         -0.044349 -0.124655  0.037988    10001 0.2908
## PC2         -0.041581 -0.167484  0.075112    10001 0.5023
## carotBeak1  -0.123129 -0.525275  0.259703    10001 0.5415
## carotLegs1   0.007916 -0.481910  0.476759    10001 0.9847
## log10mass   -0.369123 -0.785660  0.076945     9992 0.0946 .
## dietomni    -0.180453 -0.598019  0.265508    10001 0.4070
## dietplants  -0.511046 -1.039228  0.029543    10001 0.0626 .
## sexmale      0.077667 -0.087254  0.225663    10129 0.3310
## sexunknown  -0.242477 -0.530338  0.060134    10001 0.1086
## ---
## Signif. codes:  0 '***' 0.001 '**' 0.01 '*' 0.05 '.' 0.1 ' ' 1
```

MDA vs. PC1 + PC2, all data

```
set.seed(7777)

m.mda = MCMCglmm(MDA2 ~ PC1 + PC2 + carotBeak + carotLegs + log10mass + diet
```

```
+ sex, random = ~animal + species + year, pedigree = tree, prior = prior.mda,
data = data.mda, nitt = 2500001, thin = 245, burnin = 50000)

plot(m.mda)

autocorr(m.mda$VCV)

summary(m.mda)

##
##  Iterations = 50001:2500001
##  Thinning interval  = 245
##  Sample size  = 10001
##
##  DIC: 1257.894
##
##  G-structure:  ~animal
##
##        post.mean l-95% CI u-95% CI eff.samp
## animal    0.0272 0.007456  0.05165    10001
##
##                ~species
##
##         post.mean l-95% CI u-95% CI eff.samp
## species   0.01386 0.005327   0.0238    10001
##
##                ~year
##
##      post.mean l-95% CI u-95% CI eff.samp
## year    0.1561  0.02951   0.3599    10001
##
##  R-structure:  ~units
##
##       post.mean l-95% CI u-95% CI eff.samp
## units    0.1562   0.1437   0.1695    10001
##
##  Location effects: MDA2 ~ PC1 + PC2 + carotBeak + carotLegs + log10mass +
diet + sex
##
##             post.mean  l-95% CI  u-95% CI eff.samp  pMCMC
## (Intercept)  2.230206  1.790889  2.659472    10001 <1e-04 ***
## PC1         -0.010067 -0.036672  0.016054     8977 0.4568
## PC2         -0.014923 -0.054085  0.022888    10001 0.4500
## carotBeak1   0.002404 -0.122159  0.132830     9417 0.9729
## carotLegs1   0.087542 -0.072808  0.252566    10338 0.2922
## log10mass   -0.183380 -0.320459 -0.040809    10001 0.0104 *
## dietomni    -0.106570 -0.249614  0.029867    10001 0.1344
## dietplants  -0.215202 -0.389053 -0.037270    10001 0.0224 *
## sexmale     -0.014981 -0.067836  0.035042    10001 0.5651
## sexunknown   0.005465 -0.088516  0.107170    10001 0.9197
```

```
## ---
## Signif. codes:  0 '***' 0.001 '**' 0.01 '*' 0.05 '.' 0.1 ' ' 1
```

tGSH vs. PC1 + PC2, all data

```
set.seed(7777)

m.tgsh = MCMCglmm(log10tGSH ~ PC1 + PC2 + carotBeak + carotLegs + log10mass +
diet + sex, random = ~animal + species + year, pedigree = tree, prior =
prior.tgsh, data = data.tgsh, nitt = 2500001, thin = 245, burnin = 50000)

plot(m.tgsh)

autocorr(m.tgsh$VCV)

summary(m.tgsh)

##
##  Iterations = 50001:2500001
##  Thinning interval  = 245
##  Sample size  = 10001
##
##  DIC: 614.9948
##
##  G-structure:  ~animal
##
##        post.mean l-95% CI u-95% CI eff.samp
## animal   0.01202 0.003232  0.02321    10001
##
##                ~species
##
##         post.mean l-95% CI u-95% CI eff.samp
## species  0.006219  0.00227  0.01059    10252
##
##                ~year
##
##      post.mean l-95% CI u-95% CI eff.samp
## year   0.04422 0.009115   0.1044    10001
##
##  R-structure:  ~units
##
##       post.mean l-95% CI u-95% CI eff.samp
## units    0.0963  0.08829   0.1047    10001
##
##  Location effects: log10tGSH ~ PC1 + PC2 + carotBeak + carotLegs +
log10mass + diet + sex
##
##             post.mean   l-95% CI   u-95% CI eff.samp  pMCMC
## (Intercept) 0.6066335  0.3321195  0.8824766    10001 0.0002 ***
## PC1         0.0039766 -0.0160461  0.0238003     9391 0.6901
## PC2         0.0015915 -0.0273967  0.0315042    11492 0.9137
```

```
## carotBeak1   0.0037659 -0.0912972  0.1006818    10001 0.9281
## carotLegs1  -0.0214387 -0.1303494  0.1037866    10001 0.7225
## log10mass    0.1251499  0.0266628  0.2298750    10001 0.0182 *
## dietomni     0.0770258 -0.0208572  0.1817914    10001 0.1284
## dietplants   0.1580615  0.0332627  0.2773293     8991 0.0130 *
## sexmale     -0.0008688 -0.0428059  0.0426100    10001 0.9681
## sexunknown  -0.0972428 -0.1787368 -0.0234509    10001 0.0144 *
## ---
## Signif. codes:  0 '***' 0.001 '**' 0.01 '*' 0.05 '.' 0.1 ' ' 1
```

ROM vs. PC1 + PC2, all data

```
set.seed(7777)

m.rom = MCMCglmm(ROM2 ~ PC1 + PC2 + carotBeak + carotLegs + log10mass + diet
+ sex, random = ~animal + species + year, pedigree = tree, prior = prior.rom,
data = data.rom, nitt = 2500001, thin = 245, burnin = 50000)

plot(m.rom)

autocorr(m.rom$VCV)

summary(m.rom)

##
##  Iterations = 50001:2500001
##  Thinning interval  = 245
##  Sample size  = 10001
##
##  DIC: 458.0114
##
##  G-structure:  ~animal
##
##        post.mean l-95% CI u-95% CI eff.samp
## animal   0.01737 0.003704  0.03721    10001
##
##                ~species
##
##         post.mean l-95% CI u-95% CI eff.samp
## species  0.009678 0.003016  0.01848    10001
##
##                ~year
##
##      post.mean l-95% CI u-95% CI eff.samp
## year   0.07282  0.00398   0.2152    10001
##
##  R-structure:  ~units
##
##       post.mean l-95% CI u-95% CI eff.samp
## units    0.1573   0.1344   0.1794    10001
##
```

```
##  Location effects: ROM2 ~ PC1 + PC2 + carotBeak + carotLegs + log10mass + 
diet + sex 
##
##               post.mean  l-95% CI  u-95% CI eff.samp  pMCMC    
## (Intercept)   1.650873  1.193617  2.110060    10662 <1e-04 ***
## PC1           0.040338  0.004218  0.075093    10001 0.0244 *  
## PC2           0.031550 -0.020114  0.082815    10001 0.2256    
## carotBeak1    0.001926 -0.172894  0.165977    10001 0.9839    
## carotLegs1    0.079812 -0.129748  0.283474    10001 0.4436    
## log10mass    -0.192885 -0.364573 -0.013202    10001 0.0294 *  
## dietomni      0.151735 -0.015612  0.325724    10001 0.0852 .  
## dietplants    0.078736 -0.115624  0.273427     7924 0.4338    
## sexmale      -0.005122 -0.099517  0.090717    10001 0.9161    
## sexunknown    0.107579 -0.024555  0.237555    10001 0.1088    
## ---
## Signif. codes:  0 '***' 0.001 '**' 0.01 '*' 0.05 '.' 0.1 ' ' 1
```

Sensitivity analyses I:

tGSH vs. "raw" eumelanin and pheomelanin, all data

We repeated the tGSH analysis with only eumelanin or pheomelanin instead of PC1 + PC2. The relationship remains NS when the PCA scores are replaced with the "raw" eumelanin or phenomelanin scores:

```
set.seed(7777)

m.tgsh.eum = MCMCglmm(log10tGSH ~ eumelanin + carotBeak + carotLegs + 
log10mass + diet + sex, random = ~animal + species + year, pedigree = tree, 
prior = prior.tgsh, data = data.tgsh, nitt = 2500001, thin = 245, burnin = 
50000)

set.seed(7777)

m.tgsh.pheom = MCMCglmm(log10tGSH ~ pheomelanin + carotBeak + carotLegs + 
log10mass + diet + sex, random = ~animal + species + year, pedigree = tree, 
prior = prior.tgsh, data = data.tgsh, nitt = 2500001, thin = 245, burnin = 
50000)

plot(m.tgsh.eum)

autocorr(m.tgsh$VCV.eum)

plot(m.tgsh.pheom)

autocorr(m.tgsh$VCV.pheom)

summary(m.tgsh.eum)
```

```
##
##  Iterations = 50001:2500001
##  Thinning interval  = 245
##  Sample size  = 10001
##
##  DIC: 613.6874
##
##  G-structure:  ~animal
##
##        post.mean l-95% CI u-95% CI eff.samp
## animal   0.01196 0.003346   0.0227     9700
##
##                ~species
##
##         post.mean l-95% CI u-95% CI eff.samp
## species  0.006136 0.002282  0.01049    10001
##
##                ~year
##
##      post.mean l-95% CI u-95% CI eff.samp
## year   0.04511 0.009262   0.1064    10001
##
##  R-structure:  ~units
##
##       post.mean l-95% CI u-95% CI eff.samp
## units   0.09629  0.08772   0.1044    10001
##
##  Location effects: log10tGSH ~ eumelanin + carotBeak + carotLegs + 
log10mass + diet + sex
##
##             post.mean   l-95% CI   u-95% CI eff.samp  pMCMC
## (Intercept)  6.040e-01  3.202e-01  8.730e-01     9432 0.0002 ***
## eumelanin    1.573e-03 -1.726e-02  2.071e-02    10001 0.8697
## carotBeak1   3.350e-03 -9.489e-02  9.534e-02    10001 0.9433
## carotLegs1  -2.150e-02 -1.402e-01  9.588e-02    10001 0.7101
## log10mass    1.250e-01  2.151e-02  2.262e-01     8400 0.0216 *
## dietomni     7.829e-02 -1.605e-02  1.794e-01    10001 0.1136
## dietplants   1.604e-01  3.852e-02  2.845e-01    10256 0.0110 *
## sexmale      6.734e-05 -4.276e-02  4.182e-02    10001 0.9959
## sexunknown  -9.746e-02 -1.732e-01 -1.970e-02     9097 0.0124 *
## ---
## Signif. codes:  0 '***' 0.001 '**' 0.01 '*' 0.05 '.' 0.1 ' ' 1
```

```
summary(m.tgsh.pheom)
```

```
##
##  Iterations = 50001:2500001
##  Thinning interval  = 245
##  Sample size  = 10001
##
```

```
##  DIC: 613.4318
##
##  G-structure:  ~animal
##
##        post.mean l-95% CI u-95% CI eff.samp
## animal   0.01196 0.003451   0.0227     9767
##
##                ~species
##
##         post.mean l-95% CI u-95% CI eff.samp
## species  0.006104 0.002349  0.01049    10001
##
##                ~year
##
##      post.mean l-95% CI u-95% CI eff.samp
## year   0.04515 0.009576   0.1068    10001
##
##  R-structure:  ~units
##
##       post.mean l-95% CI u-95% CI eff.samp
## units   0.09627  0.08768   0.1044    10001
##
##  Location effects: log10tGSH ~ pheomelanin + carotBeak + carotLegs +
log10mass + diet + sex
##
##             post.mean  l-95% CI  u-95% CI eff.samp  pMCMC
## (Intercept)  0.621512  0.344526  0.908707     9500 <1e-04 ***
## pheomelanin -0.004693 -0.019773  0.011886    10001 0.5621
## carotBeak1   0.003392 -0.094198  0.095621    10001 0.9431
## carotLegs1  -0.020048 -0.138568  0.096244    10001 0.7277
## log10mass    0.124701  0.018172  0.217016     8145 0.0176 *
## dietomni     0.076854 -0.020794  0.174071    10001 0.1198
## dietplants   0.158161  0.035322  0.281622    10256 0.0124 *
## sexmale     -0.001426 -0.043527  0.040329    10001 0.9423
## sexunknown  -0.097126 -0.174182 -0.020839     9105 0.0134 *
## ---
## Signif. codes:  0 '***' 0.001 '**' 0.01 '*' 0.05 '.' 0.1 ' ' 1
```

ROM vs. “raw” eumelanin and pheomelanin, all data

We also repeated the ROM model with the PCA scores replaced with the “raw” melanin scores. The relationships of the two variables are marginally significant when the PCA scores are replaced with the “raw” eumelanin or phenomelanin scores:

```
set.seed(7777)

m.rom.eum = MCMCglmm(ROM2 ~ eumelanin + carotBeak + carotLegs + log10mass +
diet + sex, random = ~animal + species + year, pedigree = tree, prior =
prior.rom, data = data.rom, nitt = 2500001, thin = 245, burnin = 50000)
```

```r
set.seed(7777)

m.rom.pheom = MCMCglmm(ROM2 ~ pheomelanin + carotBeak + carotLegs + log10mass
+ diet + sex, random = ~animal + species + year, pedigree = tree, prior =
prior.rom, data = data.rom, nitt = 2500001, thin = 245, burnin = 50000)

plot(m.rom.eum)

autocorr(m.rom.eum$VCV)

plot(m.rom.pheom)

autocorr(m.rom.pheom$VCV)

summary(m.rom.eum)

##
##  Iterations = 50001:2500001
##  Thinning interval  = 245
##  Sample size  = 10001
##
##  DIC: 458.501
##
##  G-structure:  ~animal
##
##        post.mean l-95% CI u-95% CI eff.samp
## animal   0.01884  0.00379  0.04041    10110
##
##                ~species
##
##         post.mean l-95% CI u-95% CI eff.samp
## species    0.0101 0.002825  0.01929     9443
##
##                ~year
##
##      post.mean l-95% CI u-95% CI eff.samp
## year   0.07724 0.003004   0.2252    10001
##
##  R-structure:  ~units
##
##       post.mean l-95% CI u-95% CI eff.samp
## units    0.1572   0.1354   0.1807    10001
##
##  Location effects: ROM2 ~ eumelanin + carotBeak + carotLegs + log10mass +
diet + sex
##
##              post.mean   l-95% CI   u-95% CI eff.samp  pMCMC
## (Intercept)  1.6564626  1.1941294  2.0978945    10001 0.0004 ***
## eumelanin    0.0345313  0.0002263  0.0691941    10001 0.0514 .
## carotBeak1  -0.0109208 -0.1834155  0.1596786    10001 0.8921
```

```
## carotLegs1   0.0937930 -0.1164538  0.3041162    10001 0.3882
## log10mass   -0.2382350 -0.4112655 -0.0636635    10598 0.0084 **
## dietomni     0.1920600  0.0217317  0.3577744    10001 0.0256 *
## dietplants   0.1090418 -0.0885324  0.2958938    10001 0.2644
## sexmale      0.0005965 -0.0999471  0.0946558    10001 0.9929
## sexunknown   0.1053940 -0.0351722  0.2358316    10001 0.1284
## ---
## Signif. codes:  0 '***' 0.001 '**' 0.01 '*' 0.05 '.' 0.1 ' ' 1
```

```
summary(m.rom.pheom)
```

```
##
##  Iterations = 50001:2500001
##  Thinning interval  = 245
##  Sample size  = 10001
##
##  DIC: 458.6576
##
##  G-structure:  ~animal
##
##        post.mean l-95% CI u-95% CI eff.samp
## animal   0.01865 0.003621  0.04017    10168
##
##                ~species
##
##         post.mean l-95% CI u-95% CI eff.samp
## species   0.01011   0.0028  0.01931     9473
##
##                ~year
##
##      post.mean l-95% CI u-95% CI eff.samp
## year   0.07638 0.002978   0.2226    10001
##
##  R-structure:  ~units
##
##       post.mean l-95% CI u-95% CI eff.samp
## units    0.1573   0.1346     0.18    10001
##
##  Location effects: ROM2 ~ pheomelanin + carotBeak + carotLegs + log10mass
+ diet + sex
##
##              post.mean   l-95% CI   u-95% CI eff.samp  pMCMC
## (Intercept)  1.735e+00  1.266e+00  2.193e+00    10001 0.0004 ***
## pheomelanin -2.781e-02 -5.622e-02 -3.265e-05     9526 0.0516 .
## carotBeak1  -6.813e-03 -1.796e-01  1.623e-01    10001 0.9345
## carotLegs1   8.167e-02 -1.268e-01  2.917e-01    10001 0.4446
## log10mass   -2.030e-01 -3.689e-01 -2.968e-02    10625 0.0196 *
## dietomni     1.758e-01  1.145e-02  3.452e-01    10001 0.0392 *
## dietplants   1.084e-01 -9.388e-02  2.907e-01    10001 0.2664
## sexmale      7.307e-03 -8.583e-02  1.068e-01    10001 0.8817
```

```
## sexunknown   1.086e-01 -2.459e-02  2.473e-01    10001 0.1190
## ---
## Signif. codes:  0 '***' 0.001 '**' 0.01 '*' 0.05 '.' 0.1 ' ' 1
```

Iridescent colored species excluded

We repeated the analyses after excluding the species with iridescent coloration. → The ROM~PC1 relationship is near significant, all other coloration variables remained NS.

TAS vs. PC1 + PC2, iridescent excluded

```
data.tas.ua.i = subset(data.tas.ua, data.tas.ua$irridescence == "0")

p.var.tas.ua.i <- var(data.tas.ua.i$TAS2,na.rm = TRUE)

prior.tas.ua.i <- list(G = list(G1 = list(V = matrix(p.var.tas.ua.i/4),n =
1), G2 = list(V = matrix(p.var.tas.ua.i/4),n = 1), G3 = list(V =
matrix(p.var.tas.ua.i/4),n = 1)), R = list(V = matrix(p.var.tas.ua.i/4),n =
1))

set.seed(7777)

m.tas.ua.i = MCMCglmm(TAS2 ~ UA2 + PC1 + PC2 + carotBeak + carotLegs +
log10mass + diet + sex, random = ~animal + species + year, pedigree = tree,
prior = prior.tas.ua.i, data = data.tas.ua.i, nitt = 2500001, thin = 245,
burnin = 50000)

plot(m.tas.ua.i)

autocorr(m.tas.ua.i$VCV)

summary(m.tas.ua.i)

##
##  Iterations = 50001:2500001
##  Thinning interval  = 245
##  Sample size  = 10001
##
##  DIC: 603.5412
##
##  G-structure:  ~animal
##
##         post.mean l-95% CI u-95% CI eff.samp
## animal  0.007987 0.002415   0.0155    10001
##
##                ~species
##
##          post.mean l-95% CI u-95% CI eff.samp
## species  0.005636 0.002244 0.009638    10001
##
```

```
##                 ~year
##
##       post.mean l-95% CI u-95% CI eff.samp
## year    0.06981  0.01415   0.1635    10001
##
##  R-structure:  ~units
##
##        post.mean l-95% CI u-95% CI eff.samp
## units     0.1047  0.09506   0.1146    10001
##
##  Location effects: TAS2 ~ UA2 + PC1 + PC2 + carotBeak + carotLegs +
log10mass + diet + sex
##
##              post.mean  l-95% CI  u-95% CI eff.samp  pMCMC
## (Intercept)  0.918680  0.626663  1.221775    10001 <1e-04 ***
## UA2          0.073174  0.055724  0.091366    10001 <1e-04 ***
## PC1          0.001144 -0.019745  0.021755    10001  0.917
## PC2          0.005534 -0.025657  0.035592    10001  0.733
## carotBeak1   0.051206 -0.047170  0.150325    10001  0.315
## carotLegs1  -0.059118 -0.175238  0.061736    10001  0.342
## log10mass   -0.010878 -0.111107  0.094506     9687  0.832
## dietomni    -0.061506 -0.161572  0.038389    10001  0.231
## dietplants   0.015748 -0.102326  0.130714    10001  0.782
## sexmale      0.008459 -0.039422  0.056430    10366  0.718
## sexunknown  -0.003413 -0.093590  0.078765    10001  0.945
## ---
## Signif. codes:  0 '***' 0.001 '**' 0.01 '*' 0.05 '.' 0.1 ' ' 1
```

UA vs. PC1 + PC2, iridescent excluded

```
data.ua.i = subset(data.ua, data.ua$irridescence == "0")

p.var.ua.i <- var(data.ua.i$UA2,na.rm = TRUE)

prior.ua.i <- list(G = list(G1 = list(V = matrix(p.var.ua.i/4),n = 1), G2 =
list(V = matrix(p.var.ua.i/4),n = 1), G3 = list(V = matrix(p.var.ua.i/4),n =
1)), R = list(V = matrix(p.var.ua.i/4),n = 1))

set.seed(7777)

m.ua.i = MCMCglmm(UA2 ~ PC1 + PC2 + carotBeak + carotLegs + log10mass + diet
+ sex, random = ~animal + species + year, pedigree = tree, prior =
prior.ua.i, data = data.ua.i, nitt = 2500001, thin = 245, burnin = 50000)

plot(m.ua.i)

autocorr(m.ua.i$VCV)

summary(m.ua.i)
```

```
##
##  Iterations = 50001:2500001
##  Thinning interval  = 245
##  Sample size  = 10001
##
##  DIC: 3341.453
##
##  G-structure:  ~animal
##
##        post.mean l-95% CI u-95% CI eff.samp
## animal    0.1827  0.03452   0.4059    10173
##
##                ~species
##
##         post.mean l-95% CI u-95% CI eff.samp
## species    0.1717  0.06559   0.2871     8941
##
##                ~year
##
##      post.mean l-95% CI u-95% CI eff.samp
## year    0.1929  0.03123   0.4721     9225
##
##  R-structure:  ~units
##
##       post.mean l-95% CI u-95% CI eff.samp
## units      1.36    1.242    1.484    10001
##
##  Location effects: UA2 ~ PC1 + PC2 + carotBeak + carotLegs + log10mass +
diet + sex
##
##             post.mean l-95% CI u-95% CI eff.samp  pMCMC
## (Intercept)   4.91919  3.93592  5.95007    10001 <1e-04 ***
## PC1          -0.04510 -0.12830  0.04029    10001 0.3024
## PC2          -0.04018 -0.16614  0.08320    10001 0.5385
## carotBeak1    0.03516 -0.38010  0.44986    10001 0.8725
## carotLegs1   -0.16824 -0.65216  0.33984    10001 0.5055
## log10mass    -0.36042 -0.80970  0.05286    10001 0.1008
## dietomni     -0.17595 -0.60169  0.25808     8377 0.4182
## dietplants   -0.45403 -0.98716  0.06278    10001 0.0954 .
## sexmale       0.04311 -0.11868  0.21287    10001 0.6195
## sexunknown   -0.30232 -0.60562  0.01012    10295 0.0564 .
## ---
## Signif. codes:  0 '***' 0.001 '**' 0.01 '*' 0.05 '.' 0.1 ' ' 1
```

MDA vs. PC1 + PC2, iridescent excluded

```
data.mda.i = subset(data.mda, data.mda$irridescence == "0")

p.var.mda.i <- var(data.mda.i$MDA2,na.rm = TRUE)

prior.mda.i <- list(G = list(G1 = list(V = matrix(p.var.mda.i/4),n = 1), G2 =
```

```
list(V = matrix(p.var.mda.i/4),n = 1), G3 = list(V = matrix(p.var.mda.i/4),n
= 1)), R = list(V = matrix(p.var.mda.i/4),n = 1))

set.seed(7777)

m.mda.i = MCMCglmm(MDA2 ~ PC1 + PC2 + carotBeak + carotLegs + log10mass +
diet + sex, random = ~animal + species + year, pedigree = tree, prior =
prior.mda.i, data = data.mda.i, nitt = 2500001, thin = 245, burnin = 50000)

plot(m.mda.i)

autocorr(m.mda.i$VCV)

summary(m.mda.i)

##
##  Iterations = 50001:2500001
##  Thinning interval  = 245
##  Sample size  = 10001
##
##  DIC: 1156.513
##
##  G-structure:  ~animal
##
##        post.mean l-95% CI u-95% CI eff.samp
## animal   0.02837 0.007537  0.05491    10001
##
##                ~species
##
##         post.mean l-95% CI u-95% CI eff.samp
## species   0.01265 0.004243  0.02141    10001
##
##                ~year
##
##      post.mean l-95% CI u-95% CI eff.samp
## year     0.161   0.0331    0.376    10001
##
##  R-structure:  ~units
##
##       post.mean l-95% CI u-95% CI eff.samp
## units    0.1572   0.1435   0.1706    10938
##
##  Location effects: MDA2 ~ PC1 + PC2 + carotBeak + carotLegs + log10mass +
diet + sex
##
##                post.mean   l-95% CI   u-95% CI eff.samp  pMCMC
## (Intercept)  2.2293738  1.7753942  2.6681915    10001 <1e-04 ***
## PC1         -0.0093961 -0.0369184  0.0176199    10739 0.5017
## PC2         -0.0210584 -0.0612237  0.0195948    10817 0.3044
## carotBeak1   0.0553288 -0.0876391  0.1885481     9557 0.4360
```

```
## carotLegs1   0.0619930 -0.1084014  0.2259573    10001 0.4652
## log10mass   -0.1926324 -0.3394205 -0.0497715    10317 0.0096 **
## dietomni    -0.0971927 -0.2405028  0.0377839    10001 0.1780
## dietplants  -0.2003189 -0.3810205 -0.0278923    10001 0.0284 *
## sexmale     -0.0124600 -0.0675897  0.0441825    10001 0.6613
## sexunknown  -0.0007237 -0.1001904  0.0985299     9269 0.9997
## ---
## Signif. codes:  0 '***' 0.001 '**' 0.01 '*' 0.05 '.' 0.1 ' ' 1
```

tGSH vs. PC1 + PC2, iridescent excluded

```
data.tgsh.i = subset(data.tgsh, data.tgsh$irridescence == "0")

p.var.tgsh.i <- var(data.tgsh.i$log10tGSH,na.rm = TRUE)

prior.tgsh.i <- list(G = list(G1 = list(V = matrix(p.var.tgsh.i/4),n = 1), G2
= list(V = matrix(p.var.tgsh.i/4),n = 1), G3 = list(V =
matrix(p.var.tgsh.i/4),n = 1)), R = list(V = matrix(p.var.tgsh.i/4),n = 1))

set.seed(7777)

m.tgsh.i = MCMCglmm(log10tGSH ~ PC1 + PC2 + carotBeak + carotLegs + log10mass
+ diet + sex, random = ~animal + species + year, pedigree = tree, prior =
prior.tgsh.i, data = data.tgsh.i, nitt = 2500001, thin = 245, burnin = 50000)

plot(m.tgsh.i)

autocorr(m.tgsh.i$VCV)

summary(m.tgsh.i)

## 
##  Iterations = 50001:2500001
##  Thinning interval  = 245
##  Sample size  = 10001 
## 
##  DIC: 577.8767 
## 
##  G-structure:  ~animal
## 
##        post.mean l-95% CI u-95% CI eff.samp
## animal   0.01046 0.002987  0.02052    10001
## 
##                ~species
## 
##         post.mean l-95% CI u-95% CI eff.samp
## species  0.006351 0.002389    0.011    10001
## 
##                ~year
## 
##      post.mean l-95% CI u-95% CI eff.samp
```

```
## year   0.04457   0.0098   0.1058     9532
##
##  R-structure:  ~units
##
##       post.mean l-95% CI u-95% CI eff.samp
## units   0.09735  0.08867   0.1061    10001
##
##  Location effects: log10tGSH ~ PC1 + PC2 + carotBeak + carotLegs + 
log10mass + diet + sex
##
##               post.mean  l-95% CI  u-95% CI eff.samp  pMCMC
## (Intercept)   0.591347  0.326756  0.868141    10088 0.0002 ***
## PC1           0.003901 -0.016651  0.024202    10001 0.7123
## PC2           0.005842 -0.025151  0.036023    10001 0.7165
## carotBeak1   -0.001568 -0.104083  0.103439     8600 0.9847
## carotLegs1   -0.059772 -0.179672  0.064704    10001 0.3316
## log10mass     0.148304  0.043903  0.248944    10001 0.0066 **
## dietomni      0.061602 -0.046115  0.157083    10001 0.2222
## dietplants    0.156863  0.032394  0.275961    10001 0.0140 *
## sexmale      -0.010639 -0.057813  0.031319    10325 0.6371
## sexunknown   -0.117374 -0.198926 -0.040388    10492 0.0032 **
## ---
## Signif. codes:  0 '***' 0.001 '**' 0.01 '*' 0.05 '.' 0.1 ' ' 1
```

ROM vs. PC1 + PC2, iridescent excluded

```
data.rom.i = subset(data.rom, data.rom$irridescence == "0")

p.var.rom.i <- var(data.rom.i$ROM2,na.rm = TRUE)

prior.rom.i <- list(G = list(G1 = list(V = matrix(p.var.rom.i/4),n = 1), G2 = 
list(V = matrix(p.var.rom.i/4),n = 1), G3 = list(V = matrix(p.var.rom.i/4),n 
= 1)), R = list(V = matrix(p.var.rom.i/4),n = 1))

set.seed(7777)

m.rom.i = MCMCglmm(ROM2 ~ PC1 + PC2 + carotBeak + carotLegs + log10mass + 
diet + sex, random = ~animal + species + year, pedigree = tree, prior = 
prior.rom.i, data = data.rom.i, nitt = 2500001, thin = 245, burnin = 50000)

plot(m.rom.i)

autocorr(m.rom.i$VCV)

summary(m.rom.i)

##
##  Iterations = 50001:2500001
##  Thinning interval  = 245
##  Sample size  = 10001
##
```

```
##  DIC: 420.5178
##
##  G-structure:  ~animal
##
##        post.mean l-95% CI u-95% CI eff.samp
## animal   0.01901 0.003894  0.04057    10001
##
##                ~species
##
##         post.mean l-95% CI u-95% CI eff.samp
## species  0.009969 0.003177  0.01941    10001
##
##                ~year
##
##      post.mean l-95% CI u-95% CI eff.samp
## year   0.07358 0.003034   0.2104    10001
##
##  R-structure:  ~units
##
##       post.mean l-95% CI u-95% CI eff.samp
## units    0.1566   0.1333   0.1795    10001
##
##  Location effects: ROM2 ~ PC1 + PC2 + carotBeak + carotLegs + log10mass +
diet + sex
##
##             post.mean  l-95% CI  u-95% CI eff.samp  pMCMC
## (Intercept)  1.670637  1.209220  2.138122    10134 0.0002 ***
## PC1          0.035388 -0.004245  0.071750     9714 0.0706 .
## PC2          0.031554 -0.022832  0.085686    10337 0.2538
## carotBeak1  -0.022242 -0.205759  0.157041    10033 0.8227
## carotLegs1   0.106889 -0.122692  0.322839    10001 0.3540
## log10mass   -0.204463 -0.392717 -0.024259    10001 0.0300 *
## dietomni     0.153715 -0.022627  0.324192    10470 0.0912 .
## dietplants   0.077042 -0.122518  0.273434    10001 0.4276
## sexmale      0.004263 -0.104999  0.103654    10001 0.9397
## sexunknown   0.124753 -0.013373  0.259520     9229 0.0738 .
## ---
## Signif. codes:  0 '***' 0.001 '**' 0.01 '*' 0.05 '.' 0.1 ' ' 1
```

Sensitivity analyses II:

We repeated tGSH and ROM models after excluding the species with iridescent coloration, with “raw” eumelanin or pheomelanin scores instead of PC1 + PC2.

tGSH vs. “raw” eumelanin and pheomelanin, species after excluding the species with iridescent coloration excluded

```
set.seed(7777)
```

```r
m.tgsh.i.eum = MCMCglmm(log10tGSH ~ eumelanin + carotBeak + carotLegs +
log10mass + diet + sex, random = ~animal + species + year, pedigree = tree,
prior = prior.tgsh.i, data = data.tgsh.i, nitt = 2500001, thin = 245, burnin
= 50000)

set.seed(7777)

m.tgsh.i.pheom = MCMCglmm(log10tGSH ~ pheomelanin + carotBeak + carotLegs +
log10mass + diet + sex, random = ~animal + species + year, pedigree = tree,
prior = prior.tgsh.i, data = data.tgsh.i, nitt = 2500001, thin = 245, burnin
= 50000)

summary(m.tgsh.i.eum)

##
##  Iterations = 50001:2500001
##  Thinning interval  = 245
##  Sample size  = 10001
##
##  DIC: 576.6851
##
##  G-structure:  ~animal
##
##        post.mean l-95% CI u-95% CI eff.samp
## animal    0.0106 0.002686  0.02042    10521
##
##                ~species
##
##         post.mean l-95% CI u-95% CI eff.samp
## species  0.006221 0.002459  0.01082    10318
##
##                ~year
##
##      post.mean l-95% CI u-95% CI eff.samp
## year   0.04506 0.009505   0.1093    10001
##
##  R-structure:  ~units
##
##       post.mean l-95% CI u-95% CI eff.samp
## units   0.09735  0.08868   0.1063    10001
##
##  Location effects: log10tGSH ~ eumelanin + carotBeak + carotLegs +
log10mass + diet + sex
##
##              post.mean   l-95% CI   u-95% CI eff.samp  pMCMC
## (Intercept)  0.5913522  0.3135969  0.8690043     9688 0.0004 ***
## eumelanin    0.0006113 -0.0191112  0.0202766    10354 0.9441
## carotBeak1  -0.0035372 -0.1067369  0.0987923    10001 0.9581
## carotLegs1  -0.0592142 -0.1798639  0.0626958    10362 0.3324
## log10mass    0.1465910  0.0440937  0.2512556    10001 0.0072 **
```

```
## dietomni     0.0670796 -0.0281750  0.1672532    10600 0.1834
## dietplants   0.1594498  0.0403958  0.2853590    10767 0.0122 *
## sexmale     -0.0084866 -0.0513264  0.0379075    10001 0.7119
## sexunknown  -0.1183398 -0.1952638 -0.0391815    10001 0.0028 **
## ---
## Signif. codes:  0 '***' 0.001 '**' 0.01 '*' 0.05 '.' 0.1 ' ' 1
```

```
summary(m.tgsh.i.pheom)
```

```
##
##  Iterations = 50001:2500001
##  Thinning interval  = 245
##  Sample size  = 10001
##
##  DIC: 576.4161
##
##  G-structure:  ~animal
##
##        post.mean l-95% CI u-95% CI eff.samp
## animal   0.01054 0.002683  0.02026    10531
##
##                ~species
##
##         post.mean l-95% CI u-95% CI eff.samp
## species  0.006212 0.002506  0.01084    10320
##
##                ~year
##
##      post.mean l-95% CI u-95% CI eff.samp
## year   0.04513 0.009454   0.1093    10001
##
##  R-structure:  ~units
##
##       post.mean l-95% CI u-95% CI eff.samp
## units   0.09733  0.08872   0.1063    10001
##
##  Location effects: log10tGSH ~ pheomelanin + carotBeak + carotLegs +
log10mass + diet + sex
##
##             post.mean  l-95% CI  u-95% CI eff.samp  pMCMC
## (Intercept)  0.608141  0.332471  0.899995     9663 0.0004 ***
## pheomelanin -0.004460 -0.020774  0.011803    11639 0.5893
## carotBeak1  -0.002221 -0.106305  0.098549    10001 0.9801
## carotLegs1  -0.058374 -0.178952  0.063522    10363 0.3404
## log10mass    0.145711  0.042940  0.246552    10001 0.0062 **
## dietomni     0.065808 -0.030891  0.163829    10557 0.1920
## dietplants   0.156940  0.037189  0.282339    10775 0.0136 *
## sexmale     -0.010600 -0.054935  0.033488    10001 0.6379
## sexunknown  -0.118337 -0.194600 -0.038394    10001 0.0026 **
```

```
## ---
## Signif. codes:  0 '***' 0.001 '**' 0.01 '*' 0.05 '.' 0.1 ' ' 1
```

ROM vs. "raw" eumelanin and pheomelanin, species after excluding the species with iridescent coloration excluded

```
set.seed(7777)

m.rom.i.eum = MCMCglmm(ROM2 ~ eumelanin + carotBeak + carotLegs + log10mass +
diet + sex, random = ~animal + species + year, pedigree = tree, prior =
prior.rom.i, data = data.rom.i, nitt = 2500001, thin = 245, burnin = 50000)

set.seed(7777)

m.rom.i.pheom = MCMCglmm(ROM2 ~ pheomelanin + carotBeak + carotLegs +
log10mass + diet + sex, random = ~animal + species + year, pedigree = tree,
prior = prior.rom.i, data = data.rom.i, nitt = 2500001, thin = 245, burnin =
50000)

summary(m.rom.i.eum)

##
##  Iterations = 50001:2500001
##  Thinning interval  = 245
##  Sample size  = 10001
##
##  DIC: 420.8284
##
##  G-structure:  ~animal
##
##        post.mean l-95% CI u-95% CI eff.samp
## animal   0.02039 0.003947  0.04294    10001
##
##                ~species
##
##         post.mean l-95% CI u-95% CI eff.samp
## species   0.01023 0.003089   0.0198    10001
##
##                ~year
##
##      post.mean l-95% CI u-95% CI eff.samp
## year   0.08079 0.003064   0.2209    10001
##
##  R-structure:  ~units
##
##       post.mean l-95% CI u-95% CI eff.samp
## units    0.1567   0.1343   0.1804    10001
##
##  Location effects: ROM2 ~ eumelanin + carotBeak + carotLegs + log10mass +
diet + sex
##
```

```
##              post.mean  l-95% CI  u-95% CI eff.samp  pMCMC
## (Intercept)  1.671545  1.184287  2.138160    10001 0.0004 ***
## eumelanin    0.030884 -0.005786  0.069230    10001 0.1058
## carotBeak1  -0.030794 -0.214122  0.151922    10001 0.7527
## carotLegs1   0.119446 -0.100901  0.352628    10001 0.2902
## log10mass   -0.243678 -0.430435 -0.058331    10001 0.0100 **
## dietomni     0.195596  0.023214  0.371546    10001 0.0278 *
## dietplants   0.107760 -0.084954  0.301494    10001 0.2728
## sexmale      0.007730 -0.097992  0.112725    10001 0.8847
## sexunknown   0.123634 -0.007886  0.267623    10001 0.0774 .
## ---
## Signif. codes:  0 '***' 0.001 '**' 0.01 '*' 0.05 '.' 0.1 ' ' 1
```

```
summary(m.rom.i.pheom)
```

```
##
##  Iterations = 50001:2500001
##  Thinning interval  = 245
##  Sample size  = 10001
##
##  DIC: 420.7553
##
##  G-structure:  ~animal
##
##        post.mean l-95% CI u-95% CI eff.samp
## animal   0.02031 0.003839  0.04273    10001
##
##                 ~species
##
##         post.mean l-95% CI u-95% CI eff.samp
## species    0.0102 0.002729  0.01929    10001
##
##                 ~year
##
##      post.mean l-95% CI u-95% CI eff.samp
## year   0.08028 0.002919   0.2194    10001
##
##  R-structure:  ~units
##
##       post.mean l-95% CI u-95% CI eff.samp
## units    0.1567   0.1338   0.1798    10001
##
##  Location effects: ROM2 ~ pheomelanin + carotBeak + carotLegs + log10mass
+ diet + sex
##
##              post.mean  l-95% CI  u-95% CI eff.samp  pMCMC
## (Intercept)  1.750114  1.266534  2.230715    10001 0.0002 ***
## pheomelanin -0.025057 -0.053847  0.006238    10001 0.1046
## carotBeak1  -0.030168 -0.212253  0.154112    10001 0.7591
## carotLegs1   0.113976 -0.108325  0.343939    10001 0.3108
```

```
## log10mass   -0.215406 -0.395344 -0.032132    10001 0.0188 *
## dietomni     0.179922  0.003000  0.347429    10001 0.0388 *
## dietplants   0.104804 -0.086062  0.300455    10001 0.2854
## sexmale      0.015067 -0.087548  0.118260    10001 0.7789
## sexunknown   0.126825 -0.009096  0.265167    10001 0.0714 .
## ---
## Signif. codes:  0 '***' 0.001 '**' 0.01 '*' 0.05 '.' 0.1 ' ' 1
```

Species with N<3 excluded

We repeated the analyses after excluding species with N<3. The number of species excluded from each subset varied between 11 and 14 (thus, the number of analyzed species varied between 66 and 93). ROM exhibited a significant positive association with PC1, all coloration results remained qualitatively unchanged:

TAS vs. PC1 + PC2, species with N<3 excluded

```
t.tas.ua = table(data.tas.ua$species)

w.tas.ua = which(t.tas.ua<3)

names.tas.ua = names(w.tas.ua)

data.tas.ua.3 = data.tas.ua[!is.element(data.tas.ua$species, names.tas.ua),]

p.var.tas.ua.3<-var(data.tas.ua.3$TAS2,na.rm = TRUE)

prior.tas.ua.3<-list(G = list(G1 = list(V = matrix(p.var.tas.ua.3/4),n = 1),
G2 = list(V = matrix(p.var.tas.ua.3/4),n = 1), G3 = list(V =
matrix(p.var.tas.ua.3/4),n = 1)), R = list(V = matrix(p.var.tas.ua.3/4),n =
1))

set.seed(7777)

m.tas.ua.3 = MCMCglmm(TAS2 ~ UA2 + PC1 + PC2 + carotBeak + carotLegs +
log10mass + diet + sex, random = ~animal + species + year, pedigree = tree,
prior = prior.tas.ua.3, data = data.tas.ua.3,  nitt = 2500001, thin = 245,
burnin = 50000)

plot(m.tas.ua.3)

autocorr(m.tas.ua.3$VCV)

summary(m.tas.ua.3)

##
##  Iterations = 50001:2500001
##  Thinning interval  = 245
##  Sample size  = 10001
```

```
##
##  DIC: 647.2647
##
##  G-structure:  ~animal
##
##        post.mean l-95% CI u-95% CI eff.samp
## animal   0.00714  0.00247  0.01331    10001
##
##                ~species
##
##         post.mean l-95% CI u-95% CI eff.samp
## species  0.004991 0.002077 0.008426    10001
##
##                ~year
##
##      post.mean l-95% CI u-95% CI eff.samp
## year   0.06604  0.01324   0.1557    10321
##
##  R-structure:  ~units
##
##       post.mean l-95% CI u-95% CI eff.samp
## units    0.1056  0.09585   0.1149    10001
##
##  Location effects: TAS2 ~ UA2 + PC1 + PC2 + carotBeak + carotLegs + 
log10mass + diet + sex
##
##                post.mean   l-95% CI   u-95% CI eff.samp  pMCMC
## (Intercept)  0.9142424  0.6239186  1.2080327     9710 <1e-04 ***
## UA2          0.0758511  0.0582274  0.0927566     8082 <1e-04 ***
## PC1         -0.0009712 -0.0199325  0.0189639    10001  0.927
## PC2         -0.0024260 -0.0336046  0.0276301    10001  0.877
## carotBeak1   0.0585527 -0.0357177  0.1486000    10651  0.203
## carotLegs1  -0.0581001 -0.1749716  0.0510684    10001  0.316
## log10mass   -0.0233647 -0.1297225  0.0759531    10001  0.636
## dietomni    -0.0284801 -0.1312818  0.0698568    10001  0.569
## dietplants   0.0385916 -0.0740862  0.1514357    10001  0.511
## sexmale      0.0127702 -0.0327111  0.0592605    10001  0.581
## sexunknown  -0.0087769 -0.0944956  0.0791607    10001  0.838
## ---
## Signif. codes:  0 '***' 0.001 '**' 0.01 '*' 0.05 '.' 0.1 ' ' 1
```

UA vs. PC1 + PC2, species with N<3 excluded

```
t.ua = table(data.ua$species)

w.ua = which(t.ua<3)

names.ua = names(w.ua)

data.ua.3 = data.ua[!is.element(data.ua$species, names.ua),]
```

```
p.var.ua.3<-var(data.ua.3$UA2,na.rm = TRUE)

prior.ua.3<-list(G = list(G1 = list(V = matrix(p.var.ua.3/4),n = 1), G2 =
list(V = matrix(p.var.ua.3/4),n = 1), G3 = list(V = matrix(p.var.ua.3/4),n =
1)), R = list(V = matrix(p.var.ua.3/4),n = 1))

set.seed(7777)

m.ua.3 = MCMCglmm(UA2 ~ PC1 + PC2 + carotBeak + carotLegs + log10mass + diet
+ sex, random = ~animal + species + year, pedigree = tree, prior =
prior.ua.3, data = data.ua.3,  nitt = 2500001, thin = 245, burnin = 50000)

plot(m.ua.3)

autocorr(m.ua.3$VCV)

summary(m.ua.3)

##
##  Iterations = 50001:2500001
##  Thinning interval  = 245
##  Sample size  = 10001
##
##  DIC: 3615.154
##
##  G-structure:  ~animal
##
##        post.mean l-95% CI u-95% CI eff.samp
## animal    0.1871  0.03509   0.3989    10001
##
##                ~species
##
##         post.mean l-95% CI u-95% CI eff.samp
## species    0.1685  0.06754   0.2792    10001
##
##                ~year
##
##      post.mean l-95% CI u-95% CI eff.samp
## year     0.203  0.02896   0.5034    10001
##
##  R-structure:  ~units
##
##       post.mean l-95% CI u-95% CI eff.samp
## units      1.36    1.249    1.484    10001
##
##  Location effects: UA2 ~ PC1 + PC2 + carotBeak + carotLegs + log10mass +
diet + sex
##
##             post.mean l-95% CI u-95% CI eff.samp  pMCMC
## (Intercept)   4.84457  3.81958  5.83856    10001 <1e-04 ***
```

```
## PC1           -0.04678 -0.12813  0.03831    10001 0.2720
## PC2           -0.02963 -0.15706  0.09824    10001 0.6467
## carotBeak1    -0.12912 -0.53279  0.26083    10001 0.5225
## carotLegs1     0.03230 -0.47653  0.50136    10350 0.9123
## log10mass     -0.32596 -0.74750  0.12317    10001 0.1374
## dietomni      -0.22132 -0.66616  0.21657     9733 0.3248
## dietplants    -0.55652 -1.07138 -0.03006    10836 0.0378 *
## sexmale        0.06810 -0.08981  0.23013    10001 0.4028
## sexunknown    -0.18416 -0.48812  0.11488    10001 0.2288
## ---
## Signif. codes:  0 '***' 0.001 '**' 0.01 '*' 0.05 '.' 0.1 ' ' 1
```

MDA vs. PC1 + PC2, species with N<3 excluded

```
t.mda = table(data.mda$species)

w.mda = which(t.mda<3)

names.mda = names(w.mda)

data.mda.3 = data.mda[!is.element(data.mda$species, names.mda),]

p.var.mda.3<-var(data.mda.3$MDA2,na.rm = TRUE)

prior.mda.3<-list(G = list(G1 = list(V = matrix(p.var.mda.3/4),n = 1), G2 =
list(V = matrix(p.var.mda.3/4),n = 1), G3 = list(V = matrix(p.var.mda.3/4),n
= 1)), R = list(V = matrix(p.var.mda.3/4),n = 1))

set.seed(7777)

m.mda.3 = MCMCglmm(MDA2 ~ PC1 + PC2 + carotBeak + carotLegs + log10mass +
diet + sex, random = ~animal + species + year, pedigree = tree, prior =
prior.mda.3, data = data.mda.3,  nitt = 2500001, thin = 245, burnin = 50000)

plot(m.mda.3)

autocorr(m.mda.3$VCV)

summary(m.mda.3)

##
##  Iterations = 50001:2500001
##  Thinning interval  = 245
##  Sample size  = 10001
##
##  DIC: 1232.281
##
##  G-structure:  ~animal
##
##        post.mean l-95% CI u-95% CI eff.samp
## animal   0.02775  0.00753  0.05299    10001
```

```
##
##                ~species
##
##         post.mean l-95% CI u-95% CI eff.samp
## species   0.01374 0.005564  0.02383    10001
##
##                ~year
##
##      post.mean l-95% CI u-95% CI eff.samp
## year    0.1523  0.02595   0.3545    10001
##
##  R-structure:  ~units
##
##       post.mean l-95% CI u-95% CI eff.samp
## units    0.1551   0.1427   0.1687    10001
##
##  Location effects: MDA2 ~ PC1 + PC2 + carotBeak + carotLegs + log10mass +
diet + sex
##
##              post.mean  l-95% CI  u-95% CI eff.samp  pMCMC
## (Intercept)  2.262206  1.827969  2.707512    10001 <1e-04 ***
## PC1         -0.009002 -0.034727  0.018334    10001 0.5041
## PC2         -0.023067 -0.064769  0.019296    10001 0.2744
## carotBeak1   0.015939 -0.121643  0.139850    10001 0.8087
## carotLegs1   0.094624 -0.065985  0.251129    10001 0.2514
## log10mass   -0.198205 -0.342902 -0.050284    10530 0.0092 **
## dietomni    -0.105287 -0.245883  0.043286    10001 0.1536
## dietplants  -0.207949 -0.379925 -0.022970    10001 0.0240 *
## sexmale     -0.017430 -0.070774  0.033720    10001 0.5005
## sexunknown  -0.002258 -0.104053  0.094408    10001 0.9667
## ---
## Signif. codes:  0 '***' 0.001 '**' 0.01 '*' 0.05 '.' 0.1 ' ' 1
```

tGSH vs. PC1 + PC2, species with N<3 excluded

```
t.tgsh = table(data.tgsh$species)

w.tgsh = which(t.tgsh<3)

names.tgsh = names(w.tgsh)

data.tgsh.3 = data.tgsh[!is.element(data.tgsh$species, names.tgsh),]

p.var.tgsh.3<-var(data.tgsh.3$log10tGSH,na.rm = TRUE)

prior.tgsh.3<-list(G = list(G1 = list(V = matrix(p.var.tgsh.3/4),n = 1), G2 =
list(V = matrix(p.var.tgsh.3/4),n = 1), G3 = list(V =
matrix(p.var.tgsh.3/4),n = 1)), R = list(V = matrix(p.var.tgsh.3/4),n = 1))

set.seed(7777)
```

```
m.tgsh.3 = MCMCglmm(log10tGSH ~ PC1 + PC2 + carotBeak + carotLegs + log10mass
+ diet + sex, random = ~animal + species + year, pedigree = tree, prior =
prior.tgsh.3, data = data.tgsh.3,  nitt = 2500001, thin = 245, burnin =
50000)

plot(m.tgsh.3)

autocorr(m.tgsh.3$VCV)

summary(m.tgsh.3)

##
##  Iterations = 50001:2500001
##  Thinning interval  = 245
##  Sample size  = 10001
##
##  DIC: 606.2014
##
##  G-structure:  ~animal
##
##        post.mean l-95% CI u-95% CI eff.samp
## animal   0.01161 0.002976  0.02219    10001
##
##                ~species
##
##         post.mean l-95% CI u-95% CI eff.samp
## species  0.006202  0.00254  0.01089    10001
##
##                ~year
##
##      post.mean l-95% CI u-95% CI eff.samp
## year   0.04584  0.00874   0.1108    10001
##
##  R-structure:  ~units
##
##       post.mean l-95% CI u-95% CI eff.samp
## units    0.0967   0.0884   0.1052    10001
##
##  Location effects: log10tGSH ~ PC1 + PC2 + carotBeak + carotLegs +
log10mass + diet + sex
##
##             post.mean  l-95% CI  u-95% CI eff.samp  pMCMC
## (Intercept)  0.619713  0.340387  0.902602    10226 0.0002 ***
## PC1          0.001580 -0.019380  0.021441    10001 0.8763
## PC2          0.005463 -0.026023  0.036435     9655 0.7311
## carotBeak1   0.027187 -0.069496  0.125494    10001 0.5747
## carotLegs1  -0.029209 -0.147315  0.091127    10001 0.6263
## log10mass    0.115432  0.008091  0.218835    10001 0.0342 *
## dietomni     0.083237 -0.019261  0.186194     9428 0.1110
```

```
## dietplants   0.167453  0.043211  0.292813    10001 0.0108 *
## sexmale      0.004445 -0.037633  0.047677    10001 0.8327
## sexunknown  -0.095582 -0.170503 -0.017798    10001 0.0140 *
## ---
## Signif. codes:  0 '***' 0.001 '**' 0.01 '*' 0.05 '.' 0.1 ' ' 1
```

ROM vs. PC1 + PC2, species with N<3 excluded

```
t.rom = table(data.rom$species)

w.rom = which(t.rom<3)

names.rom = names(w.rom)

data.rom.3 = data.rom[!is.element(data.rom$species, names.rom),]

p.var.rom.3<-var(data.rom.3$ROM2,na.rm = TRUE)

prior.rom.3<-list(G = list(G1 = list(V = matrix(p.var.rom.3/4),n = 1), G2 =
list(V = matrix(p.var.rom.3/4),n = 1), G3 = list(V = matrix(p.var.rom.3/4),n
= 1)), R = list(V = matrix(p.var.rom.3/4),n = 1))

set.seed(7777)

m.rom.3 = MCMCglmm(ROM2 ~ PC1 + PC2 + carotBeak + carotLegs + log10mass +
diet + sex, random = ~animal + species + year, pedigree = tree, prior =
prior.rom.3, data = data.rom.3,  nitt = 2500001, thin = 245, burnin = 50000)

plot(m.rom.3)

autocorr(m.rom.3$VCV)

summary(m.rom.3)

##
##  Iterations = 50001:2500001
##  Thinning interval  = 245
##  Sample size  = 10001
##
##  DIC: 445.2381
##
##  G-structure:  ~animal
##
##        post.mean l-95% CI u-95% CI eff.samp
## animal   0.01792 0.003696  0.03869    10001
##
##                ~species
##
##         post.mean l-95% CI u-95% CI eff.samp
## species   0.01008 0.002815  0.01918    10001
##
```

```
##                  ~year
##
##          post.mean l-95% CI u-95% CI eff.samp
## year    0.07008 0.003015   0.2062    10001
##
##  R-structure:  ~units
##
##           post.mean l-95% CI u-95% CI eff.samp
## units     0.1602   0.1382   0.1846    10001
##
##  Location effects: ROM2 ~ PC1 + PC2 + carotBeak + carotLegs + log10mass +
diet + sex
##
##              post.mean  l-95% CI  u-95% CI eff.samp  pMCMC
## (Intercept)  1.666915  1.203202  2.138217    10001 0.0002 ***
## PC1          0.042910  0.005148  0.079527    10001 0.0268 *
## PC2          0.023401 -0.031760  0.075714    10001 0.3854
## carotBeak1   0.005405 -0.170796  0.173194    10001 0.9477
## carotLegs1   0.084119 -0.127167  0.301344    10001 0.4440
## log10mass   -0.204220 -0.398063 -0.021320    10001 0.0364 *
## dietomni     0.169547 -0.004912  0.358518    10001 0.0668 .
## dietplants   0.105346 -0.100232  0.298262    10001 0.2996
## sexmale     -0.009905 -0.107398  0.091680    10001 0.8447
## sexunknown   0.077439 -0.057987  0.222518    10001 0.2722
## ---
## Signif. codes:  0 '***' 0.001 '**' 0.01 '*' 0.05 '.' 0.1 ' ' 1
```

Sensitivity analyses III:

We repeated tGSH and ROM models, with N<3 excluded, with “raw” eumelanin or pheomelanin scores instead of PC1 + PC2.

tGSH vs. “raw” eumelanin and pheomelanin, species with N<3 excluded

The relationship remains NS in the tGSH models, when the PCA scores are replaced with the “raw” eumelanin or phenomelanin scores.

```
set.seed(7777)

m.tgsh.3.eum = MCMCglmm(log10tGSH ~ eumelanin + carotBeak + carotLegs +
log10mass + diet + sex, random = ~animal + species + year, pedigree = tree,
prior = prior.tgsh.3, data = data.tgsh.3, nitt = 2500001, thin = 245, burnin
= 50000)

set.seed(7777)

m.tgsh.3.pheom = MCMCglmm(log10tGSH ~ pheomelanin + carotBeak + carotLegs +
log10mass + diet + sex, random = ~animal + species + year, pedigree = tree,
```

```
prior = prior.tgsh.3, data = data.tgsh.3, nitt = 2500001, thin = 245, burnin
= 50000)

summary(m.tgsh.3.eum)

##
##  Iterations = 50001:2500001
##  Thinning interval  = 245
##  Sample size  = 10001
##
##  DIC: 604.9599
##
##  G-structure:  ~animal
##
##        post.mean l-95% CI u-95% CI eff.samp
## animal   0.01158 0.003223  0.02208    10959
##
##                ~species
##
##         post.mean l-95% CI u-95% CI eff.samp
## species  0.006132 0.002346  0.01069    10001
##
##                ~year
##
##      post.mean l-95% CI u-95% CI eff.samp
## year   0.04458 0.008748   0.1067    10001
##
##  R-structure:  ~units
##
##       post.mean l-95% CI u-95% CI eff.samp
## units   0.09661  0.08829   0.1053    10001
##
##  Location effects: log10tGSH ~ eumelanin + carotBeak + carotLegs +
log10mass + diet + sex
##
##             post.mean  l-95% CI  u-95% CI eff.samp  pMCMC
## (Intercept)  0.618726  0.348500  0.897928    10001 0.0002 ***
## eumelanin   -0.001058 -0.020950  0.017417    10001 0.9115
## carotBeak1   0.025736 -0.069379  0.122847    10001 0.6011
## carotLegs1  -0.026738 -0.147552  0.091854    10001 0.6515
## log10mass    0.114240  0.001696  0.212467    10001 0.0334 *
## dietomni     0.087425 -0.011537  0.189896     9768 0.0908 .
## dietplants   0.169252  0.048070  0.292563    10001 0.0084 **
## sexmale      0.006490 -0.035830  0.048496    10001 0.7719
## sexunknown  -0.095571 -0.174219 -0.018624    10001 0.0168 *
## ---
## Signif. codes:  0 '***' 0.001 '**' 0.01 '*' 0.05 '.' 0.1 ' ' 1

summary(m.tgsh.3.pheom)
```

```
##
##  Iterations = 50001:2500001
##  Thinning interval  = 245
##  Sample size  = 10001
##
##  DIC: 604.8725
##
##  G-structure:  ~animal
##
##        post.mean l-95% CI u-95% CI eff.samp
## animal   0.01153 0.003109  0.02189    10974
##
##               ~species
##
##         post.mean l-95% CI u-95% CI eff.samp
## species  0.006132 0.002372  0.01068    10001
##
##               ~year
##
##      post.mean l-95% CI u-95% CI eff.samp
## year    0.0446 0.008734   0.1068    10001
##
##  R-structure:  ~units
##
##       post.mean l-95% CI u-95% CI eff.samp
## units   0.09661  0.08822   0.1052    10001
##
##  Location effects: log10tGSH ~ pheomelanin + carotBeak + carotLegs +
log10mass + diet + sex
##
##             post.mean  l-95% CI  u-95% CI eff.samp  pMCMC
## (Intercept)  0.630870  0.363242  0.923775    10001 0.0002 ***
## pheomelanin -0.002894 -0.018745  0.013877    10001 0.7187
## carotBeak1   0.025669 -0.070975  0.121368    10001 0.5975
## carotLegs1  -0.024646 -0.143311  0.095915    10001 0.6803
## log10mass    0.111798  0.008884  0.213810    10001 0.0328 *
## dietomni     0.086850 -0.014252  0.187219     9698 0.0918 .
## dietplants   0.167269  0.043543  0.288047    10001 0.0098 **
## sexmale      0.004449 -0.037956  0.045662    10001 0.8391
## sexunknown  -0.095565 -0.170220 -0.014504    10001 0.0168 *
## ---
## Signif. codes:  0 '***' 0.001 '**' 0.01 '*' 0.05 '.' 0.1 ' ' 1
```

ROM vs. “raw” eumelanin and pheomelanin, species with N<3 excluded

The relationship is significantly positive with eumelanin and significantly negative with pheomelanin.

```
set.seed(7777)
```

```
m.rom.3.eum = MCMCglmm(ROM2 ~ eumelanin + carotBeak + carotLegs + log10mass +
diet + sex, random = ~animal + species + year, pedigree = tree, prior =
prior.rom.3, data = data.rom.3, nitt = 2500001, thin = 245, burnin = 50000)

set.seed(7777)

m.rom.3.pheom = MCMCglmm(ROM2 ~ pheomelanin + carotBeak + carotLegs +
log10mass + diet + sex, random = ~animal + species + year, pedigree = tree,
prior = prior.rom.3, data = data.rom.3, nitt = 2500001, thin = 245, burnin =
50000)

summary(m.rom.3.eum)

##
##  Iterations = 50001:2500001
##  Thinning interval  = 245
##  Sample size  = 10001
##
##  DIC: 444.8305
##
##  G-structure:  ~animal
##
##         post.mean l-95% CI u-95% CI eff.samp
## animal   0.01927 0.003784  0.04077    10701
##
##                ~species
##
##          post.mean l-95% CI u-95% CI eff.samp
## species   0.01036 0.003257  0.02005     9753
##
##                ~year
##
##       post.mean l-95% CI u-95% CI eff.samp
## year   0.07253 0.002706   0.2234    10001
##
##  R-structure:  ~units
##
##        post.mean l-95% CI u-95% CI eff.samp
## units    0.1602   0.1369   0.1834    10001
##
##  Location effects: ROM2 ~ eumelanin + carotBeak + carotLegs + log10mass +
diet + sex
##
##             post.mean  l-95% CI  u-95% CI eff.samp  pMCMC
## (Intercept)  1.670367  1.197861  2.133921    10410 0.0002 ***
## eumelanin    0.039149  0.003110  0.076497    10308 0.0374 *
## carotBeak1  -0.004011 -0.181145  0.167644    10147 0.9555
## carotLegs1   0.099540 -0.112218  0.322244    10001 0.3686
## log10mass   -0.252924 -0.444325 -0.059716    10380 0.0098 **
## dietomni     0.208575  0.023296  0.387298    10001 0.0244 *
```

```
## dietplants   0.133892 -0.065635  0.330706    10001 0.1884
## sexmale     -0.006572 -0.109312  0.091044    10001 0.9009
## sexunknown   0.072944 -0.068307  0.210973    10001 0.3010
## ---
## Signif. codes:  0 '***' 0.001 '**' 0.01 '*' 0.05 '.' 0.1 ' ' 1
```

```
summary(m.rom.3.pheom)
```

```
##
##  Iterations = 50001:2500001
##  Thinning interval  = 245
##  Sample size  = 10001
##
##  DIC: 445.376
##
##  G-structure:  ~animal
##
##        post.mean l-95% CI u-95% CI eff.samp
## animal   0.01917  0.00333  0.04046    10690
##
##                ~species
##
##         post.mean l-95% CI u-95% CI eff.samp
## species   0.01039 0.003094  0.01998     9765
##
##                ~year
##
##      post.mean l-95% CI u-95% CI eff.samp
## year   0.07173 0.003355   0.2215    10001
##
##  R-structure:  ~units
##
##       post.mean l-95% CI u-95% CI eff.samp
## units    0.1605   0.1381   0.1848    10001
##
##  Location effects: ROM2 ~ pheomelanin + carotBeak + carotLegs + log10mass
+ diet + sex
##
##               post.mean   l-95% CI   u-95% CI eff.samp  pMCMC
## (Intercept)  1.7548305  1.2720836  2.2246183    10431 0.0002 ***
## pheomelanin -0.0300103 -0.0602899 -0.0004923    10444 0.0484 *
## carotBeak1   0.0010088 -0.1765182  0.1713362    10001 0.9999
## carotLegs1   0.0838699 -0.1364137  0.2966344    10001 0.4538
## log10mass   -0.2125483 -0.4039802 -0.0287614    10298 0.0264 *
## dietomni     0.1902674  0.0098448  0.3701498    10001 0.0372 *
## dietplants   0.1343604 -0.0688991  0.3278511    10001 0.1876
## sexmale      0.0025131 -0.0956375  0.1007019    10001 0.9619
## sexunknown   0.0781402 -0.0668412  0.2121607    10001 0.2768
## ---
## Signif. codes:  0 '***' 0.001 '**' 0.01 '*' 0.05 '.' 0.1 ' ' 1
```

Models rerun with Prum tree

We repeated the analyses with an alternative phylogeny (Prum tree: Cooney et al 2017 Nature 542:344-347). All coloration results remained qualitatively unchanged.

```
# reading in the Prum tree

prum = read.nexus("prum.nex")

prumnames <- prum$tip

toDrop <- setdiff(prumnames, data$species)

prum104 <- drop.tip(prum, toDrop)

prum104u <- force.ultrametric(prum104)

## ***************************************************************
## *                          Note:                              *
## *    force.ultrametric does not include a formal method to    *
## *    ultrametricize a tree & should only be used to coerce    *
## *   a phylogeny that fails is.ultramtric due to rounding --   *
## *    not as a substitute for formal rate-smoothing methods.   *
## ***************************************************************
```

TAS vs. PC1 + PC2, all data, Prum tree

```
set.seed(7777)

m.tas.ua.p = MCMCglmm(TAS2 ~ UA2 + PC1 + PC2 + carotBeak + carotLegs +
log10mass + diet + sex, random = ~animal + species + year, pedigree =
prum104u, prior = prior.tas.ua, data = data.tas.ua,  nitt = 2500001, thin =
245, burnin = 50000)

plot(m.tas.ua.p)

autocorr(m.tas.ua.p$VCV)

summary(m.tas.ua.p)

##
##  Iterations = 50001:2500001
##  Thinning interval  = 245
##  Sample size  = 10001
##
##  DIC: 662.2771
##
##  G-structure:  ~animal
##
##        post.mean l-95% CI u-95% CI eff.samp
## animal  0.007477 0.002417  0.01428    10792
```

```
##
##                ~species
##
##          post.mean l-95% CI u-95% CI eff.samp
## species  0.005062 0.002109 0.008478    10001
##
##                ~year
##
##       post.mean l-95% CI u-95% CI eff.samp
## year   0.06869  0.01379   0.1615    10001
##
##  R-structure:  ~units
##
##        post.mean l-95% CI u-95% CI eff.samp
## units    0.1059  0.09646   0.1152    10951
##
##  Location effects: TAS2 ~ UA2 + PC1 + PC2 + carotBeak + carotLegs +
log10mass + diet + sex
##
##             post.mean  l-95% CI  u-95% CI eff.samp  pMCMC
## (Intercept)  0.891775  0.603550  1.187466    10001 <1e-04 ***
## UA2          0.073504  0.056215  0.090311    10001 <1e-04 ***
## PC1          0.001210 -0.018010  0.021327    10002  0.908
## PC2          0.003209 -0.026828  0.031322    10001  0.825
## carotBeak1   0.051131 -0.041261  0.139376     9579  0.274
## carotLegs1  -0.061386 -0.173817  0.056247    10554  0.294
## log10mass   -0.004295 -0.102709  0.094354    10001  0.928
## dietomni    -0.058535 -0.153156  0.039811    10001  0.236
## dietplants   0.024073 -0.088770  0.138187    10001  0.668
## sexmale      0.008796 -0.037950  0.054247    10001  0.699
## sexunknown   0.007445 -0.078474  0.095189    10001  0.868
## ---
## Signif. codes:  0 '***' 0.001 '**' 0.01 '*' 0.05 '.' 0.1 ' ' 1
```

UA vs. PC1 + PC2, all data, Prum tree

```
set.seed(7777)

m.ua.p = MCMCglmm(UA2 ~ PC1 + PC2 + carotBeak + carotLegs + log10mass + diet
+ sex, random = ~animal + species + year, pedigree = prum104u, prior =
prior.ua, data = data.ua,  nitt = 2500001, thin = 245, burnin = 50000)

plot(m.ua.p)

autocorr(m.ua.p$VCV)

summary(m.ua.p)

##
##  Iterations = 50001:2500001
##  Thinning interval  = 245
```

```
##  Sample size  = 10001
##
##  DIC: 3676.145
##
##  G-structure:  ~animal
##
##        post.mean l-95% CI u-95% CI eff.samp
## animal    0.1769  0.03464   0.3842     9674
##
##                ~species
##
##         post.mean l-95% CI u-95% CI eff.samp
## species    0.1808   0.0761   0.2956    10001
##
##                ~year
##
##      post.mean l-95% CI u-95% CI eff.samp
## year     0.195  0.03143   0.4802    10001
##
##  R-structure:  ~units
##
##       post.mean l-95% CI u-95% CI eff.samp
## units     1.363    1.245    1.477    10001
##
##  Location effects: UA2 ~ PC1 + PC2 + carotBeak + carotLegs + log10mass +
diet + sex
##
##             post.mean  l-95% CI  u-95% CI eff.samp  pMCMC
## (Intercept)  4.866424  3.903588  5.833913    10001 <1e-04 ***
## PC1         -0.044467 -0.127245  0.034765     9874 0.2840
## PC2         -0.040083 -0.160118  0.079155    10001 0.5133
## carotBeak1  -0.128647 -0.515297  0.271022    10001 0.5267
## carotLegs1   0.004323 -0.478716  0.487191    10001 0.9949
## log10mass   -0.346698 -0.778444  0.079156    10001 0.1076
## dietomni    -0.194300 -0.613689  0.251450     8494 0.3762
## dietplants  -0.494211 -1.015116  0.015940    10001 0.0636 .
## sexmale      0.074283 -0.085350  0.226761     9451 0.3550
## sexunknown  -0.241299 -0.532171  0.067746    10001 0.1180
## ---
## Signif. codes:  0 '***' 0.001 '**' 0.01 '*' 0.05 '.' 0.1 ' ' 1
```

MDA vs. PC1 + PC2, all data, Prum tree

```
set.seed(7777)

m.mda.p = MCMCglmm(MDA2 ~ PC1 + PC2 + carotBeak + carotLegs + log10mass +
diet + sex, random = ~animal + species + year, pedigree = prum104u, prior =
prior.mda, data = data.mda,  nitt = 2500001, thin = 245, burnin = 50000)
```

```
plot(m.mda.p)

autocorr(m.mda.p$VCV)

summary(m.mda.p)

##
##  Iterations = 50001:2500001
##  Thinning interval  = 245
##  Sample size  = 10001
##
##  DIC: 1258.305
##
##  G-structure:  ~animal
##
##        post.mean l-95% CI u-95% CI eff.samp
## animal   0.02716 0.007583   0.0516     9932
##
##                ~species
##
##         post.mean l-95% CI u-95% CI eff.samp
## species   0.01405 0.005581  0.02427    10001
##
##                ~year
##
##      post.mean l-95% CI u-95% CI eff.samp
## year    0.1527  0.03237   0.3544    10001
##
##  R-structure:  ~units
##
##       post.mean l-95% CI u-95% CI eff.samp
## units    0.1562   0.1435   0.1691    10001
##
##  Location effects: MDA2 ~ PC1 + PC2 + carotBeak + carotLegs + log10mass +
diet + sex
##
##             post.mean  l-95% CI  u-95% CI eff.samp  pMCMC
## (Intercept)  2.238949  1.818256  2.686832     9923 <1e-04 ***
## PC1         -0.010484 -0.037250  0.015674    10910 0.4490
## PC2         -0.014212 -0.051049  0.024740    10001 0.4596
## carotBeak1  -0.000948 -0.129062  0.129899    10001 0.9939
## carotLegs1   0.079543 -0.082754  0.242878    10001 0.3228
## log10mass   -0.184346 -0.326290 -0.040515     9983 0.0098 **
## dietomni    -0.106578 -0.251542  0.032688    10001 0.1432
## dietplants  -0.208860 -0.381932 -0.032311     9421 0.0236 *
## sexmale     -0.015037 -0.066606  0.035352    10001 0.5689
## sexunknown   0.005796 -0.090857  0.106688    10001 0.9207
## ---
## Signif. codes:  0 '***' 0.001 '**' 0.01 '*' 0.05 '.' 0.1 ' ' 1
```

tGSH vs. PC1 + PC2, all data, Prum tree

```
set.seed(7777)

m.tgsh.p = MCMCglmm(log10tGSH ~ PC1 + PC2 + carotBeak + carotLegs + log10mass
+ diet + sex, random = ~animal + species + year, pedigree = prum104u, prior =
prior.tgsh, data = data.tgsh,  nitt = 2500001, thin = 245, burnin = 50000)

plot(m.tgsh.p)

autocorr(m.tgsh.p$VCV)

summary(m.tgsh.p)

##
##  Iterations = 50001:2500001
##  Thinning interval  = 245
##  Sample size  = 10001
##
##  DIC: 614.4087
##
##  G-structure:  ~animal
##
##        post.mean l-95% CI u-95% CI eff.samp
## animal   0.01282 0.003545  0.02445    10001
##
##                ~species
##
##          post.mean l-95% CI u-95% CI eff.samp
## species  0.006106 0.002189  0.01036    10001
##
##                ~year
##
##      post.mean l-95% CI u-95% CI eff.samp
## year   0.04512 0.009433   0.1061    10001
##
##  R-structure:  ~units
##
##       post.mean l-95% CI u-95% CI eff.samp
## units   0.09621  0.08827   0.1049     9343
##
##  Location effects: log10tGSH ~ PC1 + PC2 + carotBeak + carotLegs +
log10mass + diet + sex
##
##             post.mean  l-95% CI  u-95% CI eff.samp  pMCMC
## (Intercept)  0.607662  0.324702  0.882801    10001 0.0002 ***
## PC1          0.004134 -0.015900  0.024648    10057 0.6883
## PC2          0.002492 -0.026789  0.032435    10001 0.8717
## carotBeak1   0.003341 -0.095181  0.099117    10001 0.9443
## carotLegs1  -0.024861 -0.144736  0.094288    10001 0.6869
## log10mass    0.129698  0.027128  0.234925    10001 0.0148 *
```

```
## dietomni      0.073459 -0.023115  0.178381    10001 0.1554
## dietplants    0.161489  0.039456  0.285769    10001 0.0138 *
## sexmale      -0.001187 -0.043069  0.041825    10001 0.9559
## sexunknown   -0.098001 -0.170905 -0.016640    10001 0.0132 *
## ---
## Signif. codes:  0 '***' 0.001 '**' 0.01 '*' 0.05 '.' 0.1 ' ' 1
```

ROM vs. PC1 + PC2, all data, Prum tree

```
set.seed(7777)

m.rom.p = MCMCglmm(ROM2 ~ PC1 + PC2 + carotBeak + carotLegs + log10mass +
diet + sex, random = ~animal + species + year, pedigree = prum104u, prior =
prior.rom, data = data.rom,  nitt = 2500001, thin = 245, burnin = 50000)

plot(m.rom.p)

autocorr(m.rom.p$VCV)

summary(m.rom.p)

##
##  Iterations = 50001:2500001
##  Thinning interval  = 245
##  Sample size  = 10001
##
##  DIC: 457.7928
##
##  G-structure:  ~animal
##
##        post.mean l-95% CI u-95% CI eff.samp
## animal   0.01768 0.003741  0.03749     9708
##
##                ~species
##
##         post.mean l-95% CI u-95% CI eff.samp
## species  0.009548 0.002989  0.01809     9671
##
##                ~year
##
##      post.mean l-95% CI u-95% CI eff.samp
## year   0.07516  0.00291   0.2272    10001
##
##  R-structure:  ~units
##
##       post.mean l-95% CI u-95% CI eff.samp
## units    0.1571   0.1357   0.1801    10001
##
##  Location effects: ROM2 ~ PC1 + PC2 + carotBeak + carotLegs + log10mass +
diet + sex
##
```

```
##               post.mean  l-95% CI  u-95% CI eff.samp  pMCMC
## (Intercept)  1.673917  1.211412  2.134258    10001 0.0012 **
## PC1          0.040716  0.004012  0.076647    10001 0.0290 *
## PC2          0.031746 -0.019044  0.084633    10001 0.2280
## carotBeak1   0.004664 -0.163027  0.171509    10001 0.9541
## carotLegs1   0.074347 -0.142527  0.277316    10001 0.4778
## log10mass   -0.202255 -0.386092 -0.034481     9712 0.0242 *
## dietomni     0.157766 -0.008446  0.331632    10001 0.0670 .
## dietplants   0.086849 -0.102672  0.280975    10001 0.3714
## sexmale     -0.006904 -0.099973  0.093467    10001 0.8917
## sexunknown   0.104553 -0.032046  0.235515    10001 0.1250
## ---
## Signif. codes:  0 '***' 0.001 '**' 0.01 '*' 0.05 '.' 0.1 ' ' 1
```

Sensitivity analyses IV:

We repeated tGSH and ROM models, with the Prum tree, with only eumelanin or pheomelanin instead of PC1 + PC2.

tGSH vs. "raw" eumelanin and pheomelanin, with Prum tree

```
set.seed(7777)

m.tgsh.p.eum = MCMCglmm(log10tGSH ~ eumelanin + carotBeak + carotLegs +
log10mass + diet + sex, random = ~animal + species + year, pedigree =
prum104u, prior = prior.tgsh, data = data.tgsh, nitt = 2500001, thin = 245,
burnin = 50000)

set.seed(7777)

m.tgsh.p.pheom = MCMCglmm(log10tGSH ~ pheomelanin + carotBeak + carotLegs +
log10mass + diet + sex, random = ~animal + species + year, pedigree =
prum104u, prior = prior.tgsh, data = data.tgsh, nitt = 2500001, thin = 245,
burnin = 50000)

summary(m.tgsh.p.eum)

##
##  Iterations = 50001:2500001
##  Thinning interval  = 245
##  Sample size  = 10001
##
##  DIC: 613.1915
##
##  G-structure:  ~animal
##
##        post.mean l-95% CI u-95% CI eff.samp
## animal   0.01281  0.00365  0.02468    10001
##
##               ~species
##
```

```
##          post.mean l-95% CI u-95% CI eff.samp
## species  0.006033 0.002257  0.01037     9839
##
##                ~year
##
##      post.mean l-95% CI u-95% CI eff.samp
## year   0.04459 0.009104   0.1057    10001
##
##  R-structure:  ~units
##
##       post.mean l-95% CI u-95% CI eff.samp
## units   0.09623  0.08822   0.1046    10001
##
##  Location effects: log10tGSH ~ eumelanin + carotBeak + carotLegs +
log10mass + diet + sex
##
##              post.mean   l-95% CI   u-95% CI eff.samp  pMCMC
## (Intercept)  6.053e-01  3.198e-01  8.717e-01    10052 0.0004 ***
## eumelanin    1.708e-03 -1.869e-02  1.974e-02    10001 0.8601
## carotBeak1   2.759e-03 -9.356e-02  9.763e-02    10001 0.9607
## carotLegs1  -2.450e-02 -1.467e-01  8.864e-02    10001 0.6797
## log10mass    1.278e-01  2.280e-02  2.333e-01    10001 0.0168 *
## dietomni     7.643e-02 -2.286e-02  1.733e-01    10001 0.1238
## dietplants   1.642e-01  4.470e-02  2.919e-01    10001 0.0098 **
## sexmale      4.949e-05 -4.151e-02  4.430e-02    10001 0.9975
## sexunknown  -9.846e-02 -1.777e-01 -2.434e-02    10001 0.0150 *
## ---
## Signif. codes:  0 '***' 0.001 '**' 0.01 '*' 0.05 '.' 0.1 ' ' 1
```

```
summary(m.tgsh.p.pheom)
```

```
##
##  Iterations = 50001:2500001
##  Thinning interval  = 245
##  Sample size  = 10001
##
##  DIC: 612.9031
##
##  G-structure:  ~animal
##
##        post.mean l-95% CI u-95% CI eff.samp
## animal   0.01281 0.003437  0.02437    10001
##
##                ~species
##
##          post.mean l-95% CI u-95% CI eff.samp
## species  0.005998 0.002255  0.01029     9767
##
##                ~year
##
```

```
##      post.mean l-95% CI u-95% CI eff.samp
## year    0.04464 0.008452   0.1052    10001
##
##  R-structure:  ~units
##
##       post.mean l-95% CI u-95% CI eff.samp
## units   0.09621  0.08826   0.1046    10001
##
##  Location effects: log10tGSH ~ pheomelanin + carotBeak + carotLegs +
log10mass + diet + sex
##
##              post.mean  l-95% CI  u-95% CI eff.samp  pMCMC
## (Intercept)  0.623514  0.338791  0.899514     9988 0.0004 ***
## pheomelanin -0.004931 -0.020964  0.010774    10001 0.5519
## carotBeak1   0.002738 -0.092959  0.098113    10001 0.9611
## carotLegs1  -0.022727 -0.136701  0.098442    10001 0.7061
## log10mass    0.127603  0.025757  0.228211    10001 0.0146 *
## dietomni     0.074843 -0.029029  0.167165    10001 0.1324
## dietplants   0.161825  0.045450  0.293723    10001 0.0110 *
## sexmale     -0.001502 -0.045440  0.039775    10001 0.9499
## sexunknown  -0.098126 -0.175652 -0.022145    10001 0.0156 *
## ---
## Signif. codes:  0 '***' 0.001 '**' 0.01 '*' 0.05 '.' 0.1 ' ' 1
```

ROM vs. “raw” eumelanin and pheomelanin, with Prum tree

```
set.seed(7777)

m.rom.p.eum = MCMCglmm(ROM2 ~ eumelanin + carotBeak + carotLegs + log10mass +
diet + sex, random = ~animal + species + year, pedigree = prum104u, prior =
prior.rom, data = data.rom, nitt = 2500001, thin = 245, burnin = 50000)

set.seed(7777)

m.rom.p.pheom = MCMCglmm(ROM2 ~ pheomelanin + carotBeak + carotLegs +
log10mass + diet + sex, random = ~animal + species + year, pedigree =
prum104u, prior = prior.rom, data = data.rom, nitt = 2500001, thin = 245,
burnin = 50000)

summary(m.rom.p.eum)

##
##  Iterations = 50001:2500001
##  Thinning interval  = 245
##  Sample size  = 10001
##
##  DIC: 458.4385
##
##  G-structure:  ~animal
##
##        post.mean l-95% CI u-95% CI eff.samp
```

```
## animal   0.01946 0.003778  0.04136    10001
##
##                ~species
##
##          post.mean l-95% CI u-95% CI eff.samp
## species   0.01005 0.003125  0.01938    10001
##
##                ~year
##
##      post.mean l-95% CI u-95% CI eff.samp
## year   0.07041 0.003114   0.2133    10001
##
##  R-structure:  ~units
##
##       post.mean l-95% CI u-95% CI eff.samp
## units    0.1573   0.1344    0.179    10001
##
##  Location effects: ROM2 ~ eumelanin + carotBeak + carotLegs + log10mass +
diet + sex
##
##              post.mean   l-95% CI   u-95% CI eff.samp  pMCMC
## (Intercept)  1.6747486  1.2138768  2.1468578    12794 0.0002 ***
## eumelanin    0.0346596 -0.0007378  0.0690466    10605 0.0534 .
## carotBeak1  -0.0077905 -0.1725377  0.1689743    10001 0.9293
## carotLegs1   0.0873177 -0.1224247  0.3039253    10001 0.4230
## log10mass   -0.2458101 -0.4187734 -0.0615876    10001 0.0100 **
## dietomni     0.1937677  0.0231970  0.3656452    10001 0.0290 *
## dietplants   0.1163714 -0.0708598  0.3107717    10001 0.2294
## sexmale      0.0007088 -0.0975269  0.0919151    10474 0.9905
## sexunknown   0.1039775 -0.0211709  0.2431470    10001 0.1202
## ---
## Signif. codes:  0 '***' 0.001 '**' 0.01 '*' 0.05 '.' 0.1 ' ' 1
```

```
summary(m.rom.p.pheom)
```

```
##
##  Iterations = 50001:2500001
##  Thinning interval  = 245
##  Sample size  = 10001
##
##  DIC: 458.5566
##
##  G-structure:  ~animal
##
##        post.mean l-95% CI u-95% CI eff.samp
## animal    0.0193  0.00357  0.04098    10001
##
##                ~species
##
##          post.mean l-95% CI u-95% CI eff.samp
```

```
## species   0.01003 0.003104  0.01937    10001
##
##                  ~year
##
##      post.mean l-95% CI u-95% CI eff.samp
## year   0.06973 0.003301    0.211    10001
##
##  R-structure:  ~units
##
##       post.mean l-95% CI u-95% CI eff.samp
## units    0.1574   0.1348   0.1794    10001
##
##  Location effects: ROM2 ~ pheomelanin + carotBeak + carotLegs + log10mass
+ diet + sex
##
##               post.mean   l-95% CI   u-95% CI eff.samp  pMCMC
## (Intercept)  1.7539976  1.2738526  2.2216863    10001 0.0002 ***
## pheomelanin -0.0280029 -0.0562757  0.0002043    11352 0.0514 .
## carotBeak1  -0.0031107 -0.1677129  0.1733537    10001 0.9753
## carotLegs1   0.0753835 -0.1404122  0.2850707    10001 0.4838
## log10mass   -0.2104286 -0.3824369 -0.0375604    10001 0.0192 *
## dietomni     0.1778694  0.0032674  0.3413369    10001 0.0406 *
## dietplants   0.1157313 -0.0679276  0.3133864    10001 0.2304
## sexmale      0.0073564 -0.0852894  0.1016547    10455 0.8835
## sexunknown   0.1073212 -0.0214613  0.2430155    10001 0.1068
## ---
## Signif. codes:  0 '***' 0.001 '**' 0.01 '*' 0.05 '.' 0.1 ' ' 1
```

Plots for Supplementary Material

```
# TAS2 means vs. PC1 and PC2, all data

data.tas.ua %>%
 group_by(species) %>%
 summarize(species = species, yvalue = mean(TAS2), xvalue = mean(PC1)) %>%
 distinct() %>%
 ggplot(aes(xvalue, yvalue)) +
 geom_point(shape = 1, size = 3) +
 theme(legend.position = "none",
       text = element_text(size = 10),
       panel.background = element_blank(),
       axis.line = element_line(color = "black")) +
  ylab(expression("Total antioxidant status (mM trolox)")) +
  xlab("Melanin pigment dimension (PC1)")

## `summarise()` has grouped output by 'species'. You can override using the
`.groups` argument.
```

```
# TAS2 means vs. PC1 and PC2, all data

ggp1 <- data.tas.ua %>%
 group_by(species) %>%
 summarize(species = species, yvalue = mean(TAS2), xvalue = mean(PC1), n = n()) %>%
 distinct() %>%
 ggplot(aes(xvalue, yvalue, size = n)) +
 geom_point(shape = 21) +
 theme(panel.background = element_blank(),
       axis.line = element_line(color = "black")) +
  ylab(expression("Total antioxidant status (mM trolox)")) +
  xlab("Melanin pigment dimension (PC1)")
```

```
## `summarise()` has grouped output by 'species'. You can override using the
## `.groups` argument.
```

```
print(ggp1)
```

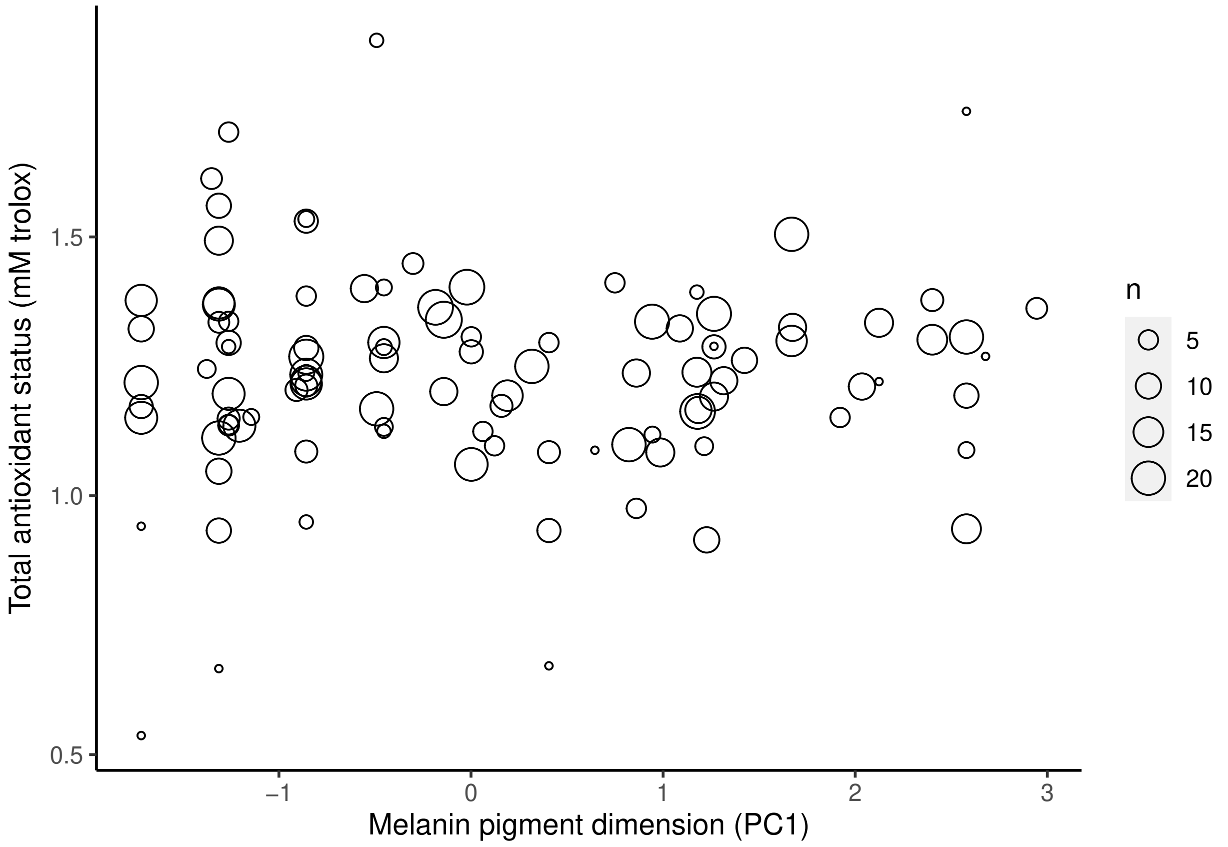


```
ggp2 <- data.tas.ua %>%
 group_by(species) %>%
 summarize(species = species, yvalue = mean(TAS2), xvalue = mean(PC2), n = n()) %>%
 distinct() %>%
 ggplot(aes(xvalue, yvalue, size = n)) +
 geom_point(shape = 21) +
```

```
 theme(panel.background = element_blank(),
       axis.line = element_line(color = "black")) +
 ylab(expression("Total antioxidant status (mM trolox)")) +
 xlab("Carotenoid pigment dimension (PC2)")
```

```
## `summarise()` has grouped output by 'species'. You can override using the
## `.groups` argument.
```

```
print(ggp2)
```

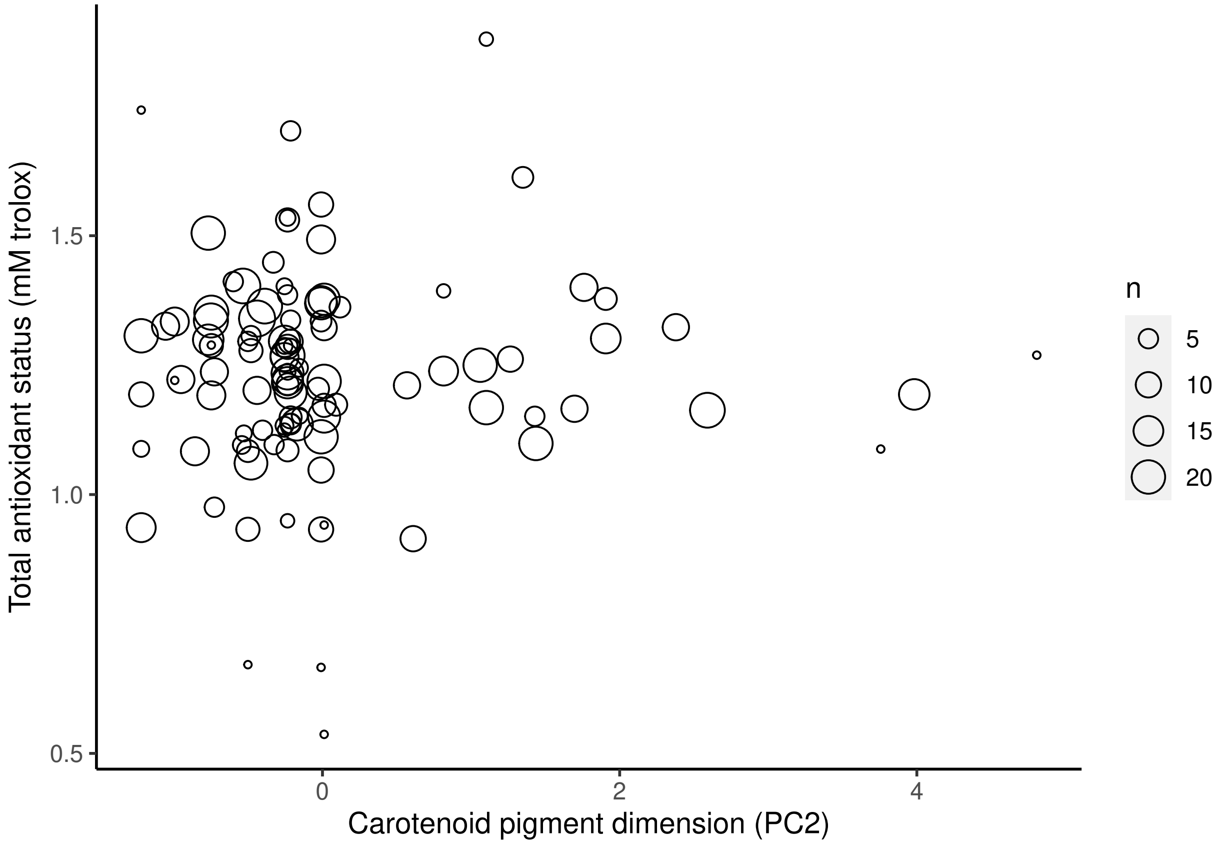


```
# UA2 means vs. PC1 and PC2, all data

ggp3 <- data.ua %>%
 group_by(species) %>%
 summarize(species = species, yvalue = mean(UA2), xvalue = mean(PC1), n = n()) %>%
 distinct() %>%
 ggplot(aes(xvalue, yvalue, size = n)) +
 geom_point(shape =  21) +
 theme(panel.background = element_blank(),
       axis.line = element_line(color = "black")) +
 ylab(expression("Uric acid (mg/dL)")) +
 xlab("Melanin pigment dimension (PC1)")
```

```
## `summarise()` has grouped output by 'species'. You can override using the
## `.groups` argument.
```

```
print(ggp3)
```

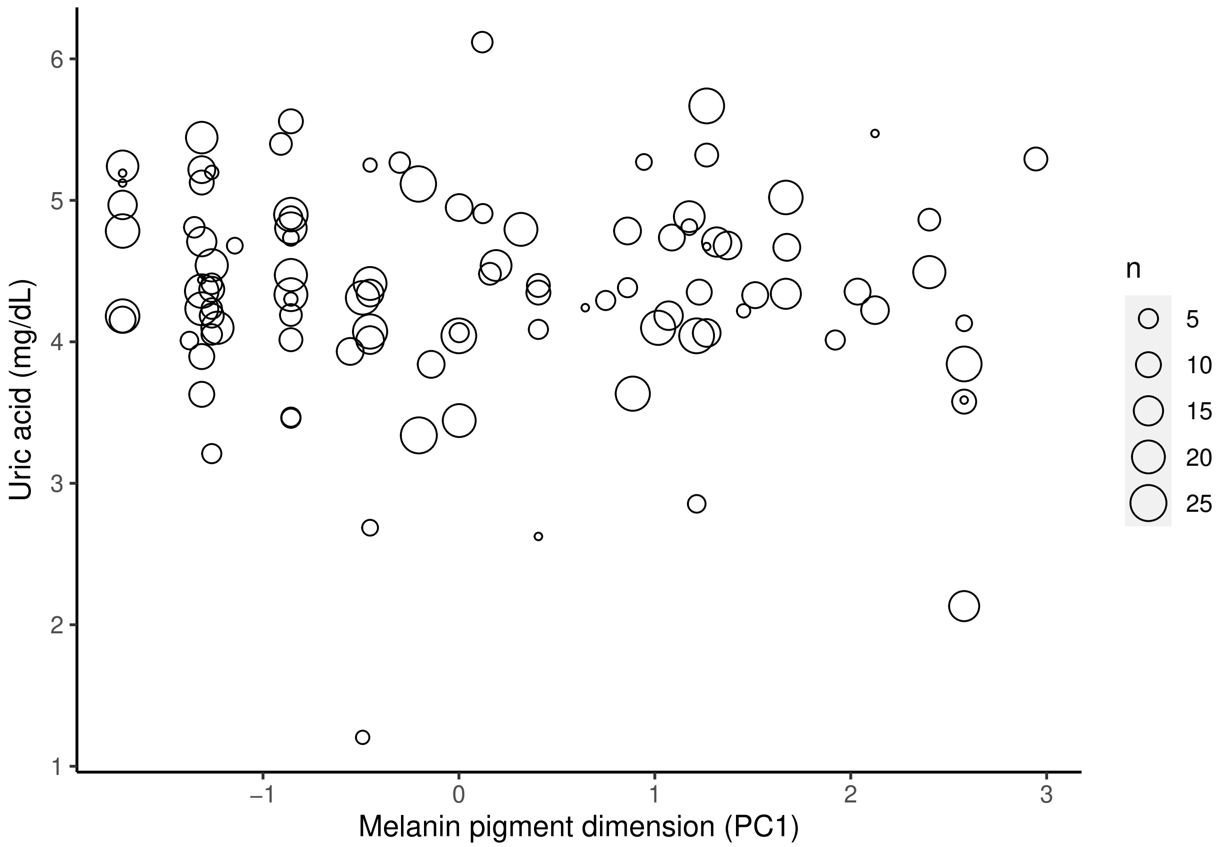


```
ggp4 <- data.ua %>%
 group_by(species) %>%
 summarize(species = species, yvalue = mean(UA2), xvalue = mean(PC2), n = n()) %>%
 distinct() %>%
 ggplot(aes(xvalue, yvalue, size = n)) +
 geom_point(shape =  21) +
 theme(panel.background = element_blank(),
       axis.line = element_line(color = "black")) +
 ylab(expression("Uric acid (mg/dL)")) +
 xlab("Carotenoid pigment dimension (PC2)")
```

```
## `summarise()` has grouped output by 'species'. You can override using the
## `.groups` argument.
```

```
print(ggp4)
```

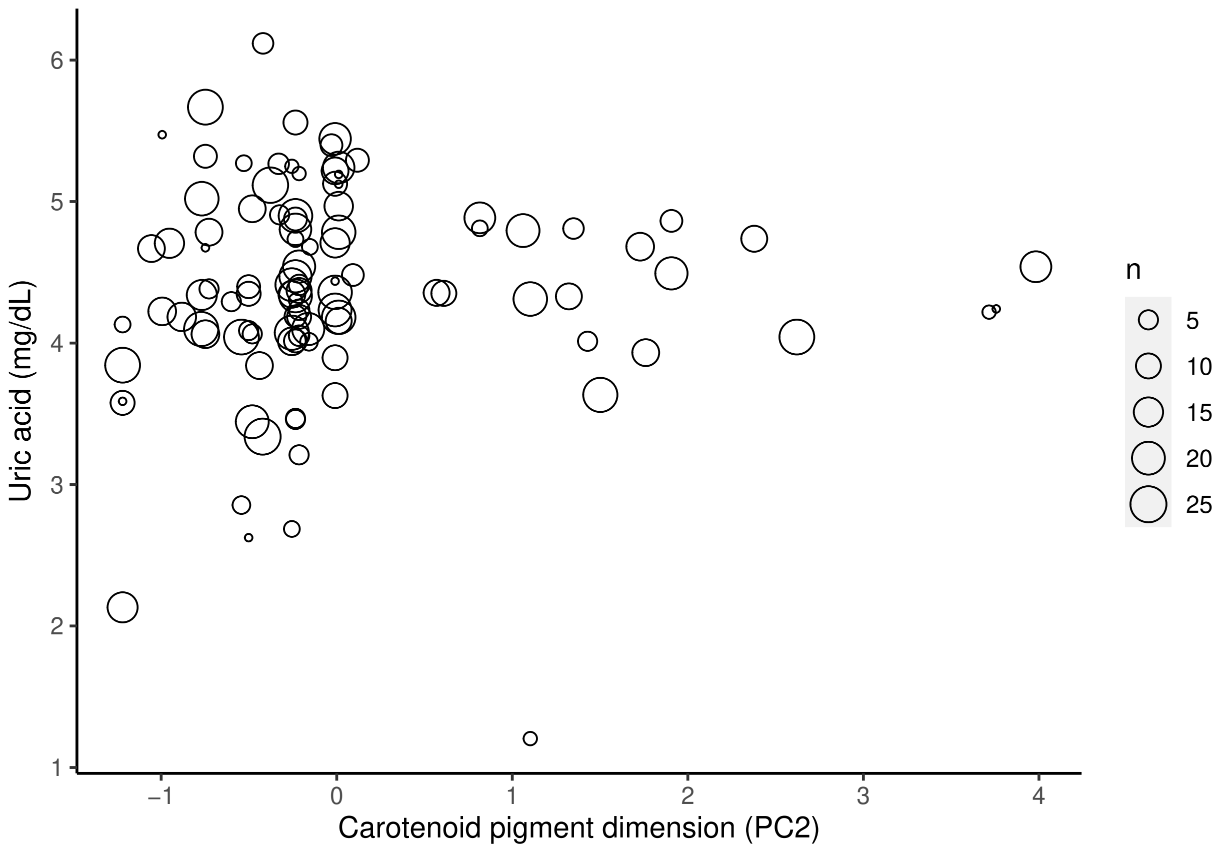


```
# MDA means vs. PC1 and PC2, all data

ggp5 <- data.mda %>%
 group_by(species) %>%
 summarize(species = species, yvalue = mean(MDA2), xvalue = mean(PC1), n = n()) %>%
 distinct() %>%
 ggplot(aes(xvalue, yvalue, size = n)) +
 geom_point(shape =  21) +
 theme(panel.background = element_blank(),
       axis.line = element_line(color = "black")) +
 ylab(expression(Malondialdehyde ~ (mu * g ~ mL^-1))) +
 xlab("Melanin pigment dimension (PC1)")
```

```
## `summarise()` has grouped output by 'species'. You can override using the
## `.groups` argument.
```

```
print(ggp5)
```

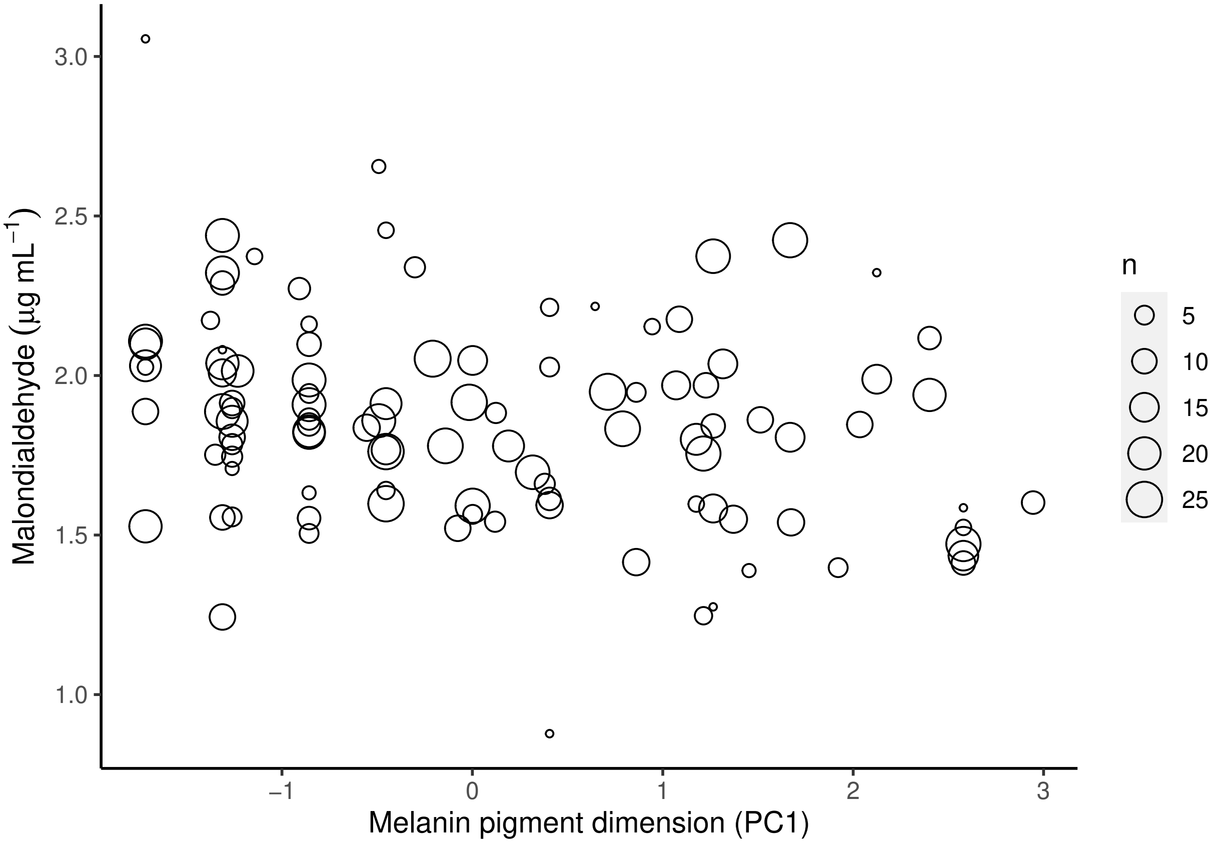


```
ggp6 <- data.mda %>%
 group_by(species) %>%
 summarize(species = species, yvalue = mean(MDA2), xvalue = mean(PC2), n = n()) %>%
 distinct() %>%
 ggplot(aes(xvalue, yvalue, size = n)) +
 geom_point(shape =  21) +
 theme(panel.background = element_blank(),
       axis.line = element_line(color = "black")) +
 ylab(expression(Malondialdehyde ~ (mu * g ~ mL^-1))) +
 xlab("Carotenoid pigment dimension (PC2)")
```

```
## `summarise()` has grouped output by 'species'. You can override using the
## `.groups` argument.
```

```
print(ggp6)
```

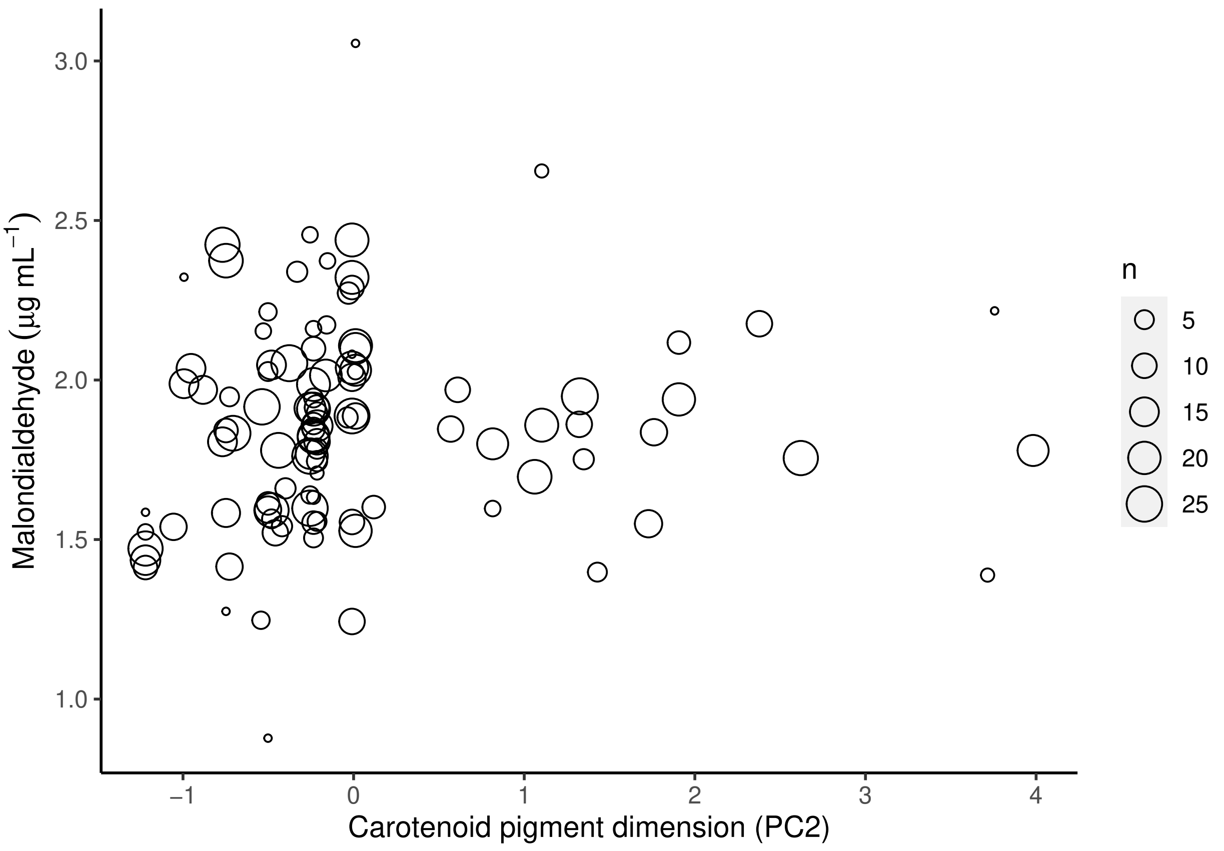


```
# log10tGSH means vs. PC1 and PC2, all data

ggp7 <- data.tgsh %>%
 group_by(species) %>%
 summarize(species = species, yvalue = mean(log10tGSH), xvalue = mean(PC1), n = n()) %>%
 distinct() %>%
 ggplot(aes(xvalue, yvalue, size = n)) +
 geom_point(shape =  21) +
 theme(panel.background = element_blank(),
       axis.line = element_line(color = "black")) +
 ylab(expression("Total gluthatione content (nM/mg)")) +
 xlab("Melanin pigment dimension (PC1)")
```

```
## `summarise()` has grouped output by 'species'. You can override using the
## `.groups` argument.
```

```
print(ggp7)
```

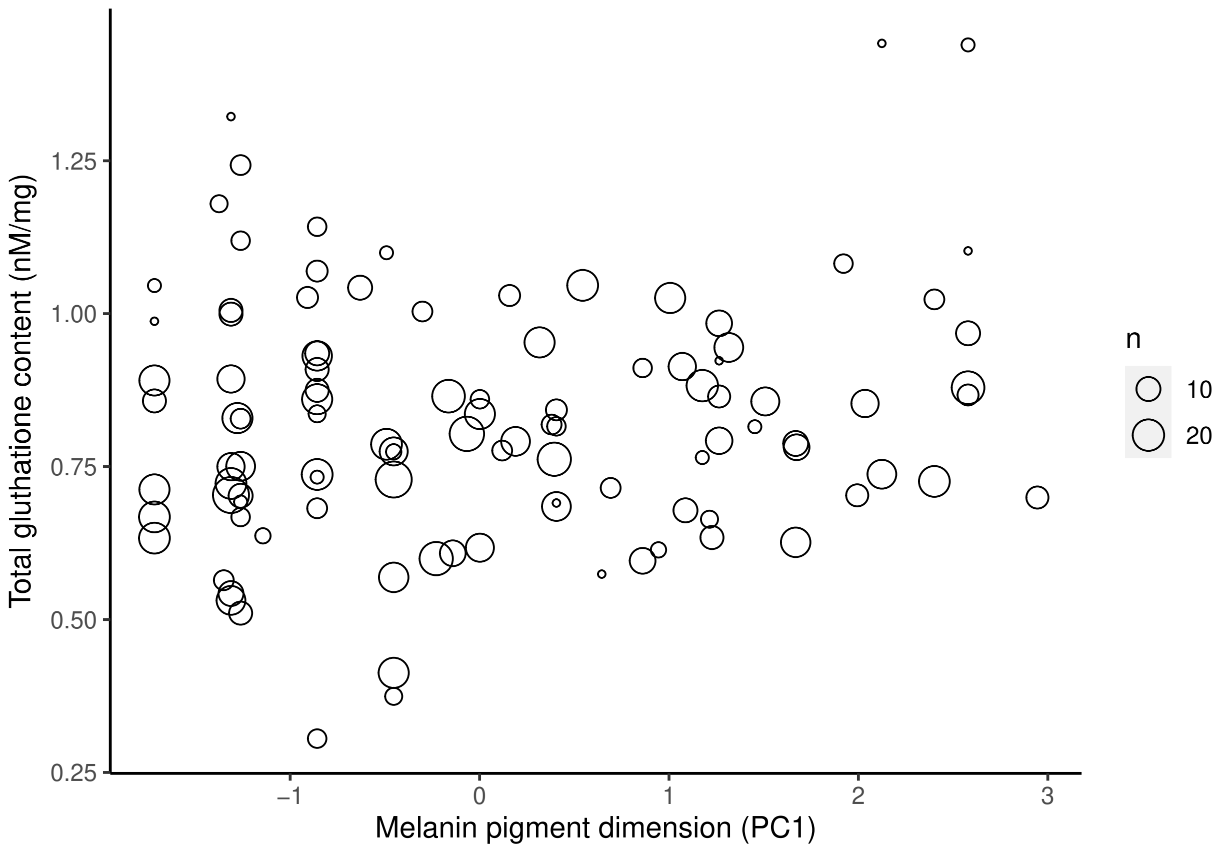


```
ggp8 <- data.tgsh %>%
 group_by(species) %>%
 summarize(species = species, yvalue = mean(log10tGSH), xvalue = mean(PC2), n = n()) %>%
 distinct() %>%
 ggplot(aes(xvalue, yvalue, size = n)) +
 geom_point(shape =  21) +
 theme(panel.background = element_blank(),
       axis.line = element_line(color = "black")) +
 ylab(expression("Total gluthatione content (nM/mg)")) +
 xlab("Carotenoid pigment dimension (PC2)")
```

```
## ‘summarise()‘ has grouped output by ’species’. You can override using the
## ‘.groups‘ argument.
```

```
print(ggp8)
```

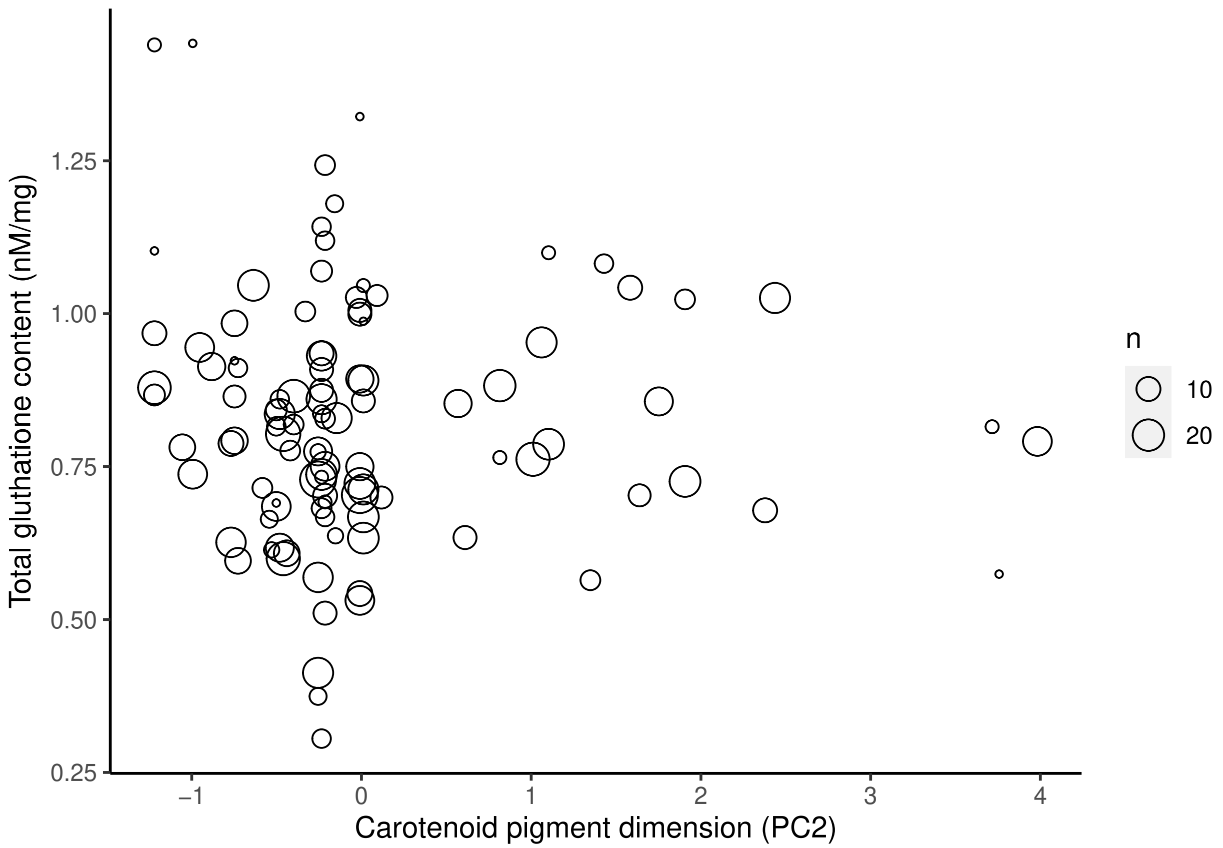


```
# ROM2 means vs. PC1 and PC2, all data

ggp9 <- data.rom %>%
 group_by(species) %>%
 summarize(species = species, yvalue = mean(ROM2), xvalue = mean(PC1), n = n()) %>%
 distinct() %>%
 ggplot(aes(xvalue, yvalue, size = n)) +
 geom_point(shape =  21) +
 theme(panel.background = element_blank(),
       axis.line = element_line(color = "black")) +
 ylab(expression("Reactive oxygen metabolites (mM H "[2]*"O"[2]*")")) +
 xlab("Melanin pigment dimension (PC1)")
```

```
## ‘summarise()‘ has grouped output by ’species’. You can override using the
## ‘.groups‘ argument.
```

```
print(ggp9)
```

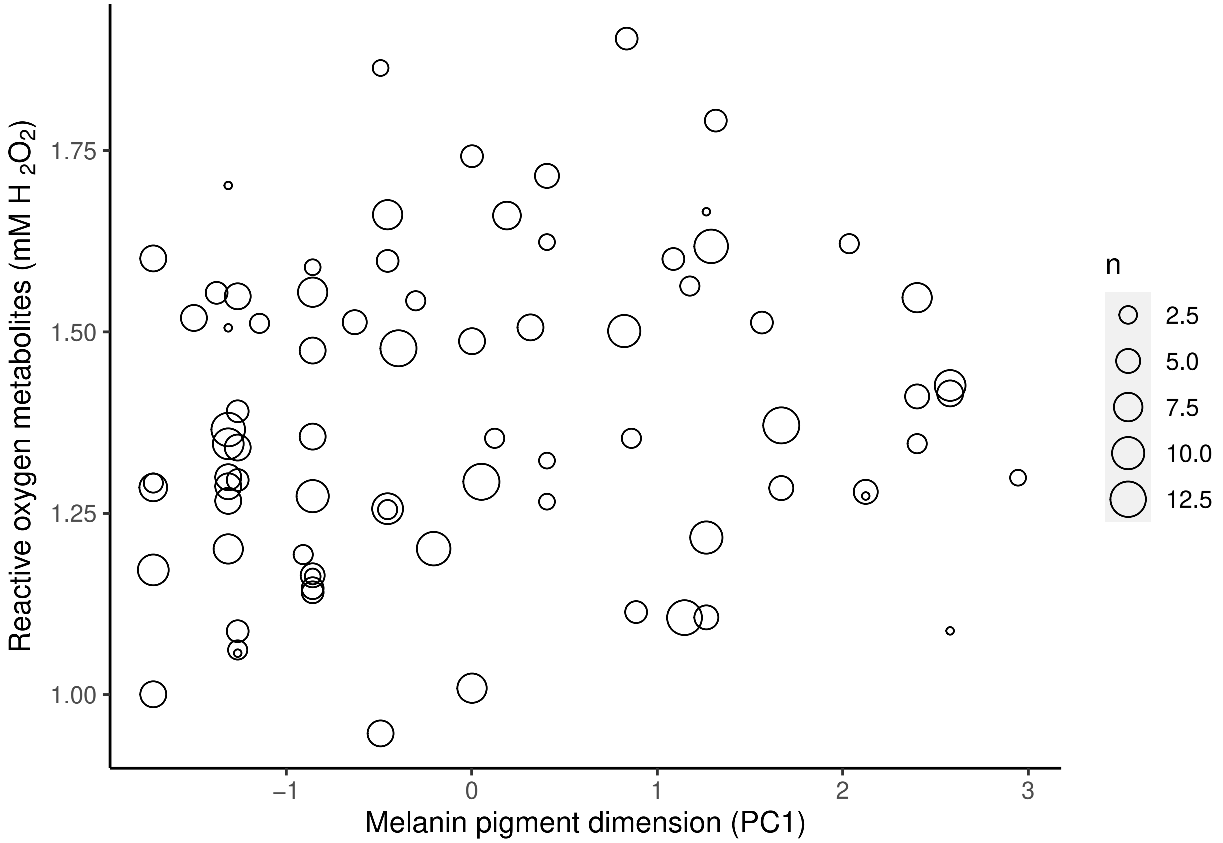


```
ggp10 <- data.rom %>%
 group_by(species) %>%
 summarize(species = species, yvalue = mean(ROM2), xvalue = mean(PC2), n = n()) %>%
 distinct() %>%
 ggplot(aes(xvalue, yvalue, size = n)) +
 geom_point(shape =  21) +
 theme(panel.background = element_blank(),
       axis.line = element_line(color = "black")) +
 ylab(expression("Reactive oxygen metabolites (mM H "[2]*"O"[2]*")")) +
 xlab("Carotenoid pigment dimension (PC2)")
```

```
## `summarise()` has grouped output by 'species'. You can override using the
## `.groups` argument.
```

```
print(ggp10)
```

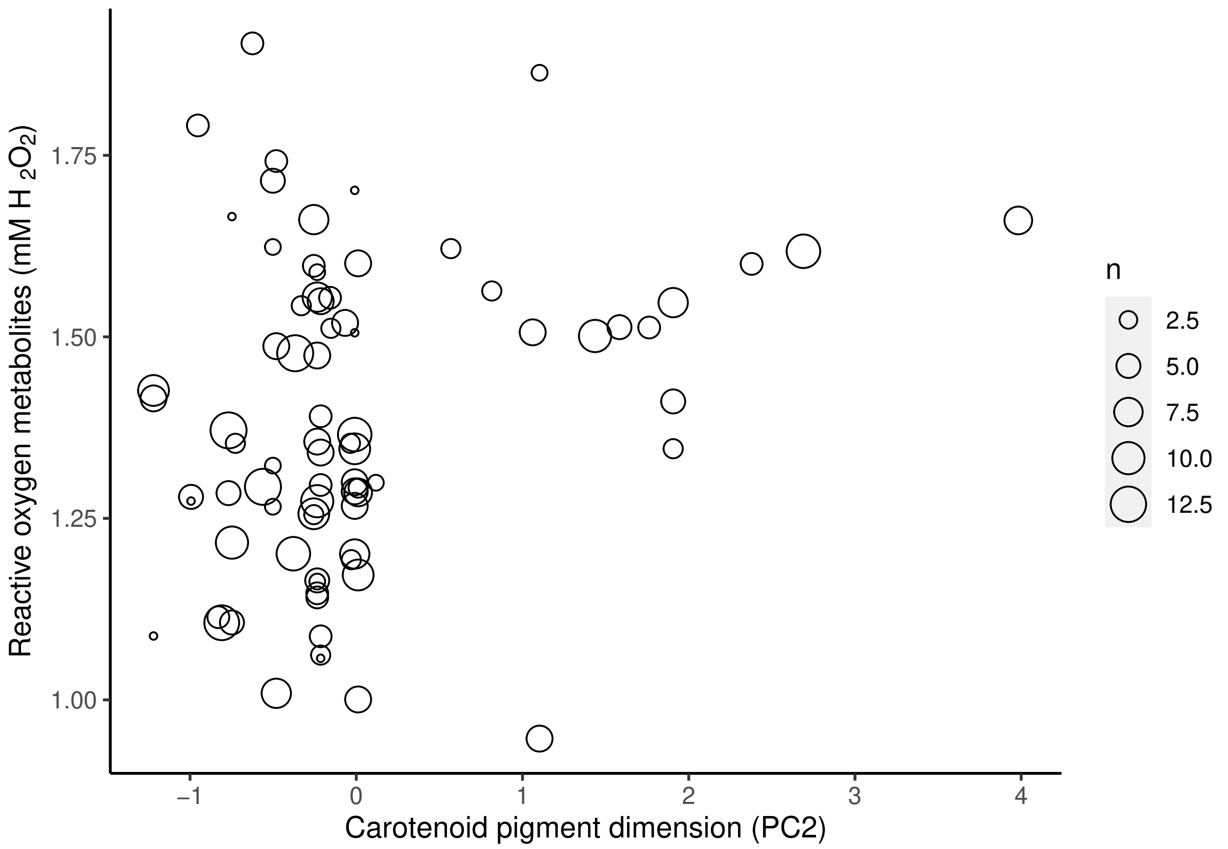


```r
# TAS2 means vs. eumelanin and pheomelanin, all data

ggp11 <- data.tas.ua %>%
 group_by(species) %>%
 summarize(species = species, yvalue = mean(TAS2), xvalue = mean(eumelanin), n = n()) %>%
 distinct() %>%
 ggplot(aes(xvalue, yvalue, size = n)) +
 geom_point(shape =  21) +
 theme(panel.background = element_blank(),
       axis.line = element_line(color = "black")) +
 ylab(expression("Total antioxidant status (mM trolox)")) +
 xlab("Plumage eumelanin content")
```

```
## `summarise()` has grouped output by 'species'. You can override using the
## `.groups` argument.
```

```r
print(ggp11)
```

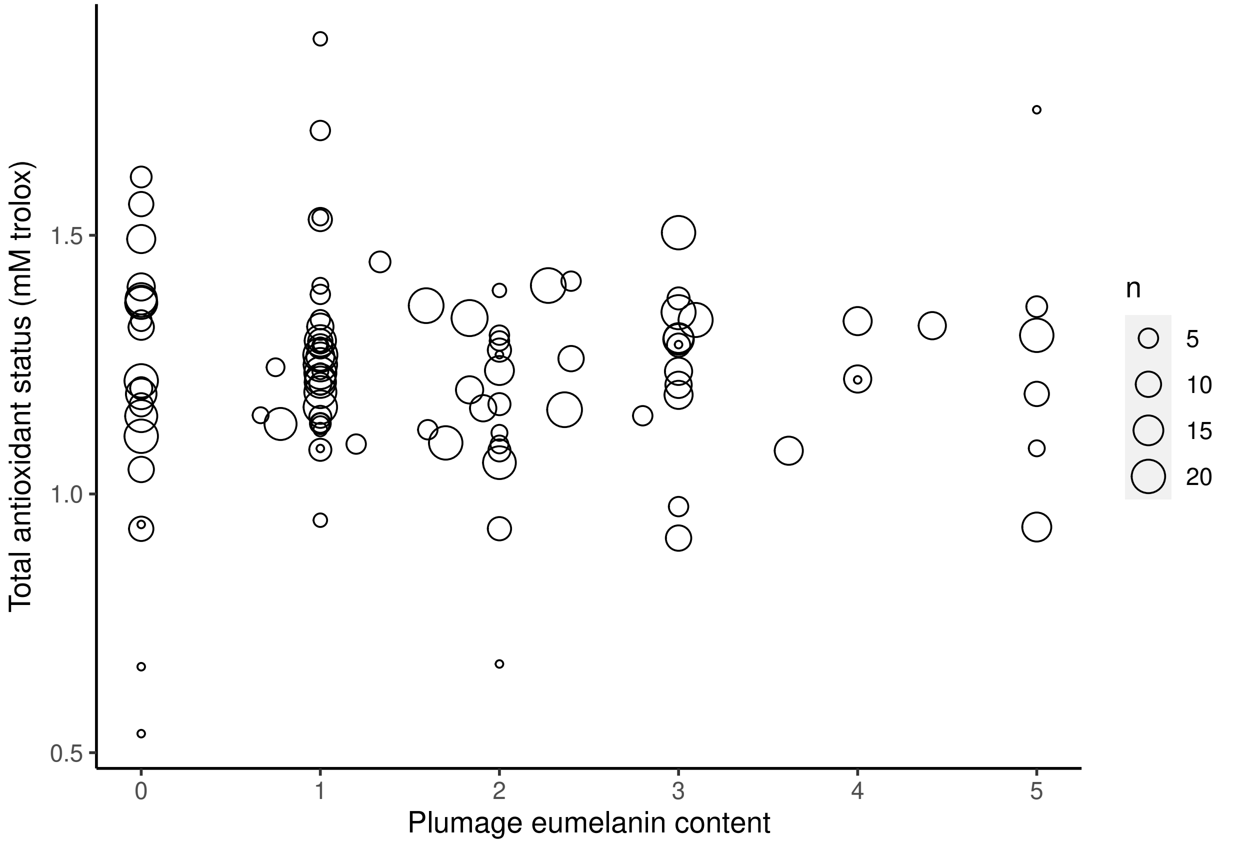


```
ggp12 <- data.tas.ua %>%
 group_by(species) %>%
 summarize(species = species, yvalue = mean(TAS2), xvalue = mean(pheomelanin), n = n()) %>%
 distinct() %>%
 ggplot(aes(xvalue, yvalue, size = n)) +
 geom_point(shape =  21) +
 theme(panel.background = element_blank(),
       axis.line = element_line(color = "black")) +
 ylab(expression("Total antioxidant status (mM trolox)")) +
 xlab("Plumage pheomelanin content")
```

```
## `summarise()` has grouped output by 'species'. You can override using the
## `.groups` argument.
```

```
print(ggp12)
```

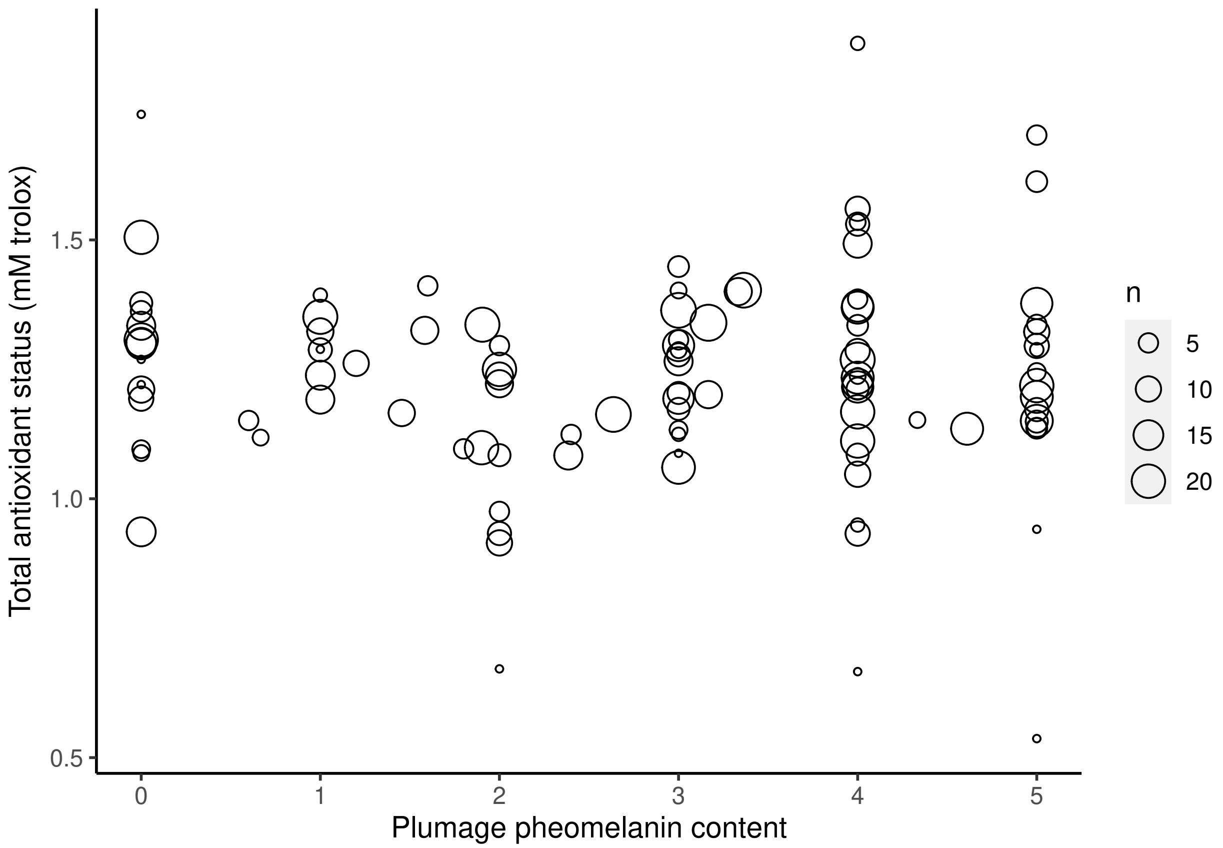


```
# UA2 means vs. eumelanin and pheomelanin, all data

ggp13 <- data.ua %>%
 group_by(species) %>%
 summarize(species = species, yvalue = mean(UA2), xvalue = mean(eumelanin), n = n()) %>%  distinct() %>%
 ggplot(aes(xvalue, yvalue, size = n)) +
 geom_point(shape =  21) +
 theme(panel.background = element_blank(),
       axis.line = element_line(color = "black")) +
 ylab(expression("Uric acid (mg/dL)")) +
 xlab("Plumage eumelanin content")
```

```
## 'summarise()' has grouped output by 'species'. You can override using the
## '.groups' argument.
```

```
print(ggp13)
```

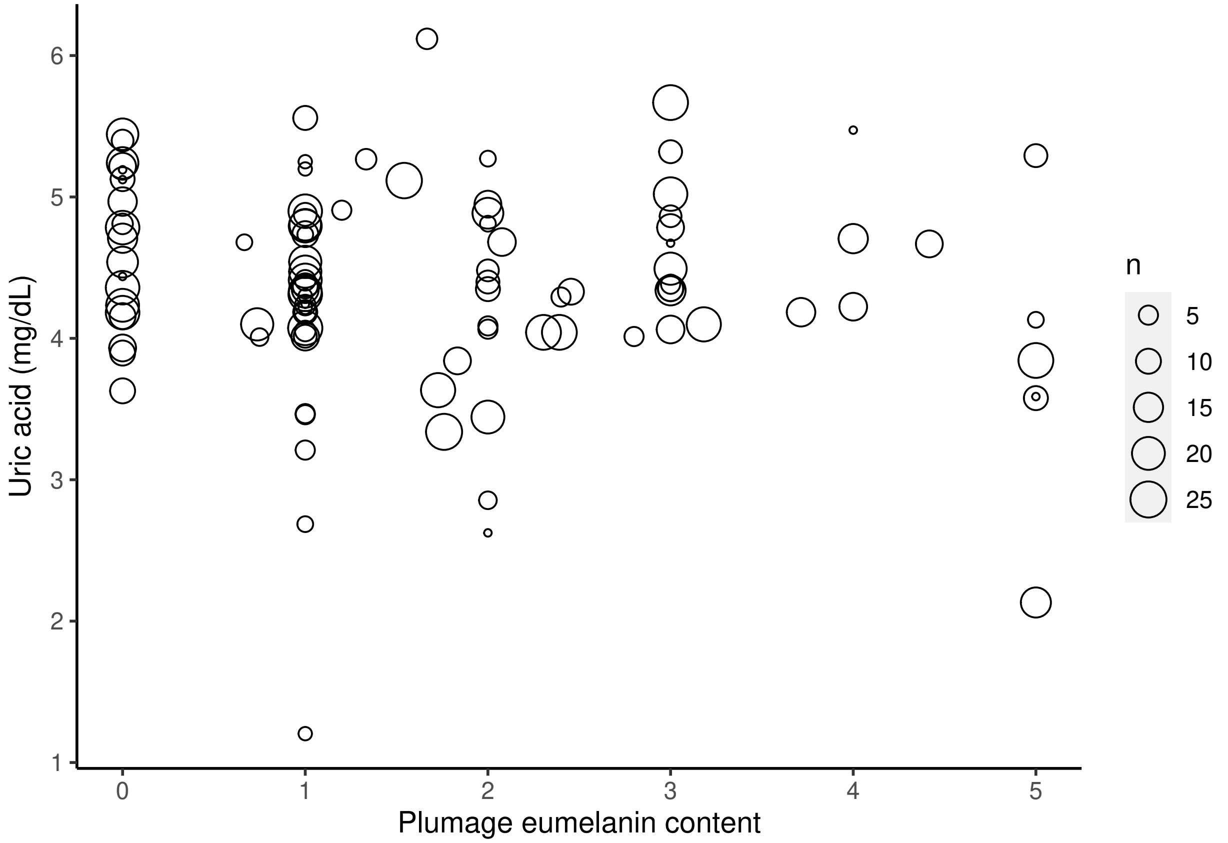


```
ggp14 <- data.ua %>%
 group_by(species) %>%
 summarize(species = species, yvalue = mean(UA2), xvalue = mean(pheomelanin), n = n()) %>%
 distinct() %>%
 ggplot(aes(xvalue, yvalue, size = n)) +
 geom_point(shape =  21) +
 theme(panel.background = element_blank(),
       axis.line = element_line(color = "black")) +
 ylab(expression("Uric acid (mg/dL)")) +
 xlab("Plumage pheomelanin content")
```

```
## ‘summarise()‘ has grouped output by ’species’. You can override using the
## ‘.groups‘ argument.
```

```
print(ggp14)
```

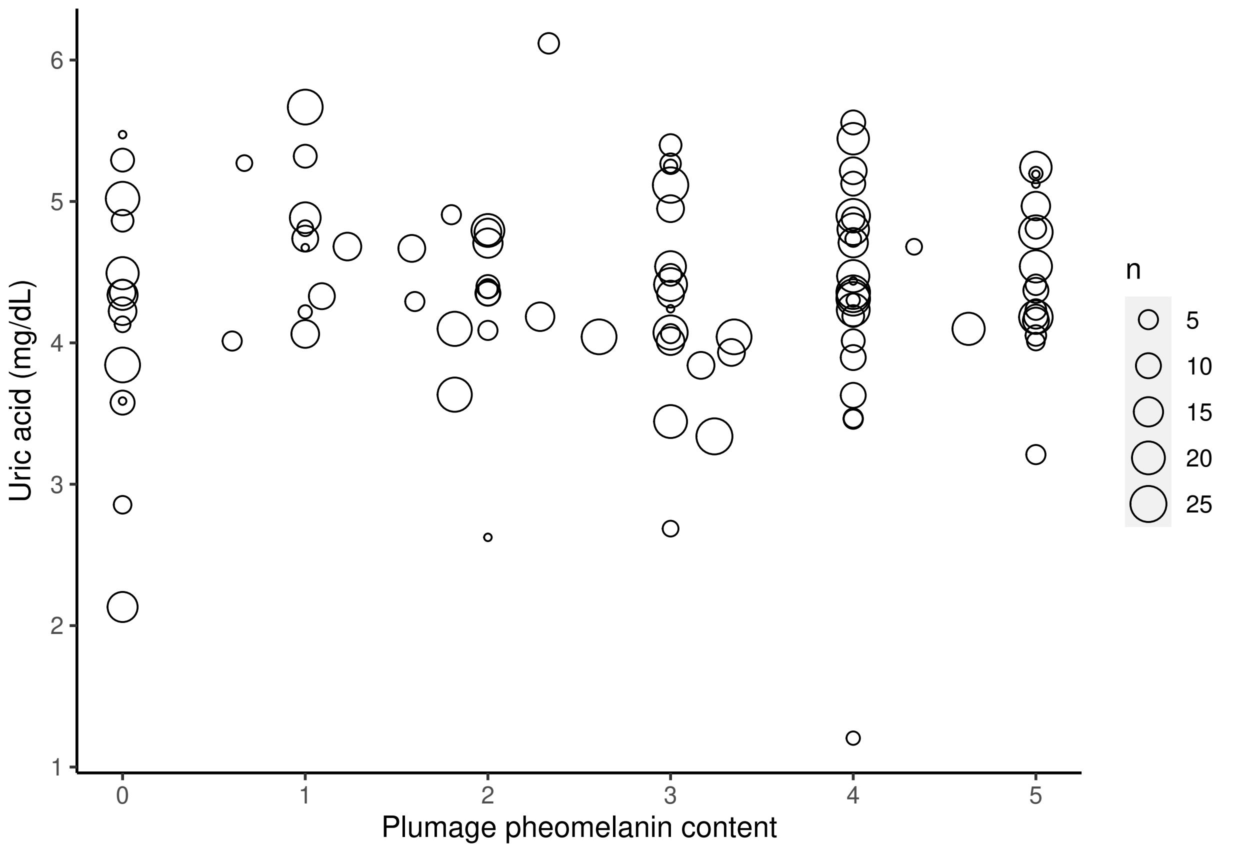


```
# MDA means vs. eumelanin and pheomelanin, all data

ggp15 <- data.mda %>%
 group_by(species) %>%
 summarize(species = species, yvalue = mean(MDA2), xvalue = mean(eumelanin), n = n()) %>%
 distinct() %>%
 ggplot(aes(xvalue, yvalue, size = n)) +
 geom_point(shape =  21) +
 theme(panel.background = element_blank(),
       axis.line = element_line(color = "black")) +
 ylab(expression(Malondialdehyde ~ (mu * g ~ mL^-1))) +
 xlab("Plumage eumelanin content")
```

```
## `summarise()` has grouped output by 'species'. You can override using the
## `.groups` argument.
```

```
print(ggp15)
```

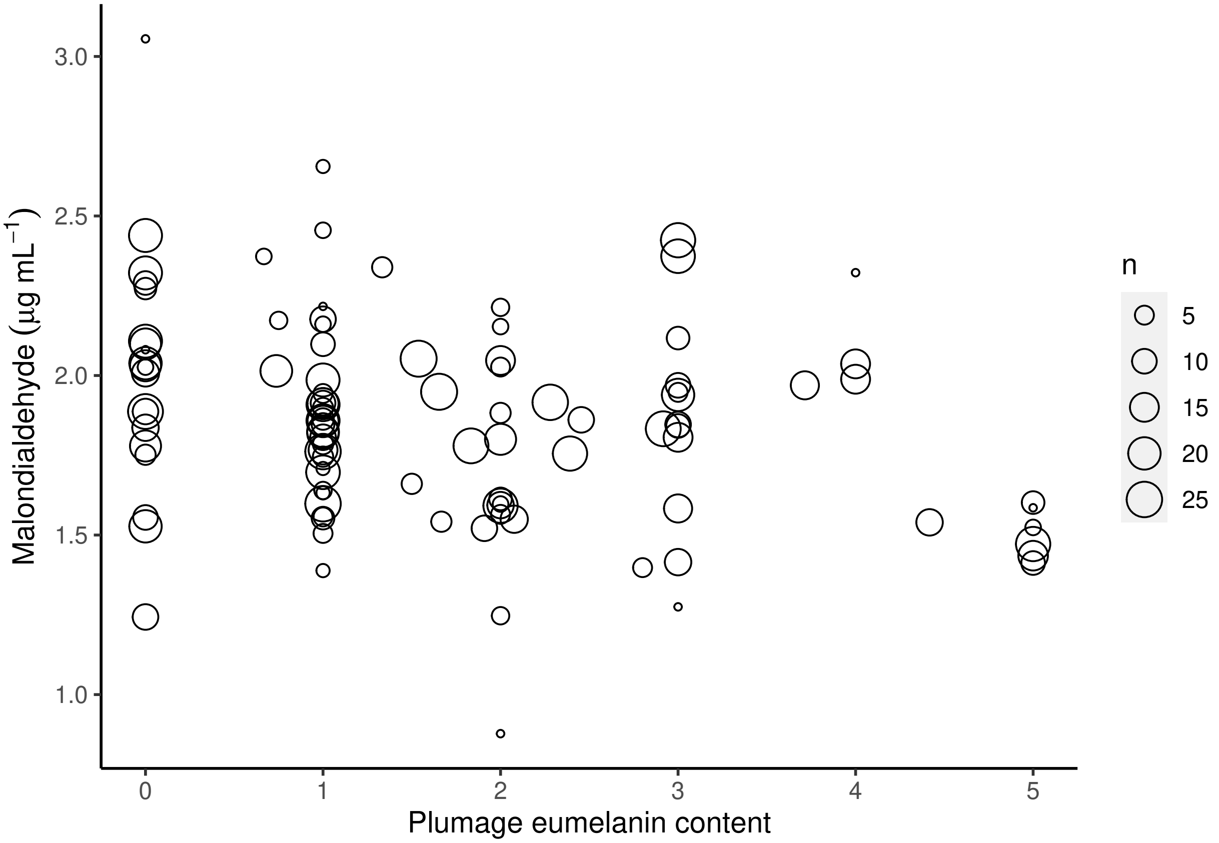


```
ggp16 <- data.mda %>%
 group_by(species) %>%
 summarize(species = species, yvalue = mean(MDA2), xvalue = mean(pheomelanin), n = n()) %>%
 distinct() %>%
 ggplot(aes(xvalue, yvalue, size = n)) +
 geom_point(shape =  21) +
 theme(panel.background = element_blank(),
       axis.line = element_line(color = "black")) +
 ylab(expression(Malondialdehyde ~ (mu * g ~ mL^-1))) +
 xlab("Plumage pheomelanin content")
```

```
## `summarise()` has grouped output by 'species'. You can override using the
## `.groups` argument.
```

```
print(ggp16)
```

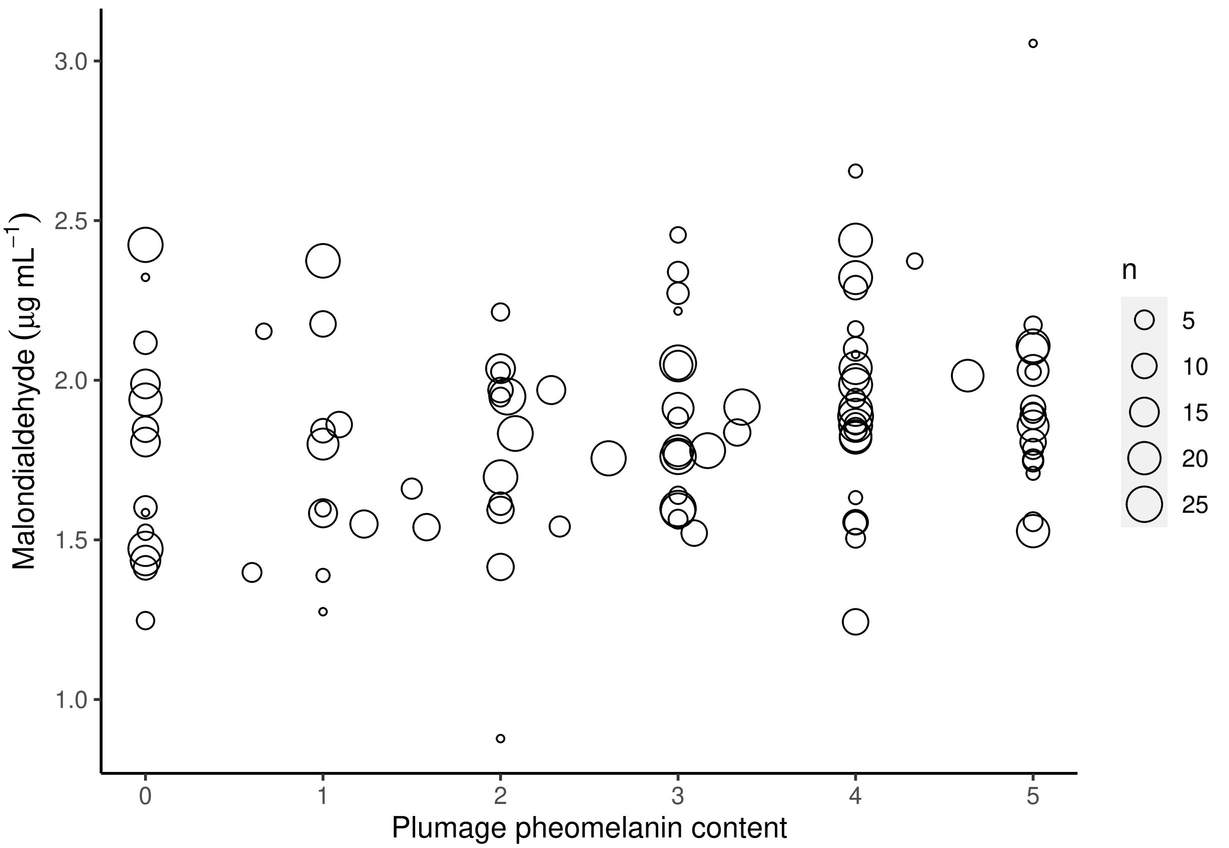


```
# log10tGSH means vs. eumelanin and pheomelanin, all data

ggp17 <- data.tgsh %>%
 group_by(species) %>%
 summarize(species = species, yvalue = mean(log10tGSH), xvalue = mean(eumelanin), n = n()) %>%
 distinct() %>%
 ggplot(aes(xvalue, yvalue, size = n)) +
 geom_point(shape =  21) +
 theme(panel.background = element_blank(),
       axis.line = element_line(color = "black")) +
 ylab(expression("Total gluthatione content (nM/mg)")) +
 xlab("Plumage eumelanin content")
```

```
## `summarise()` has grouped output by 'species'. You can override using the
## `.groups` argument.
```

```
print(ggp17)
```

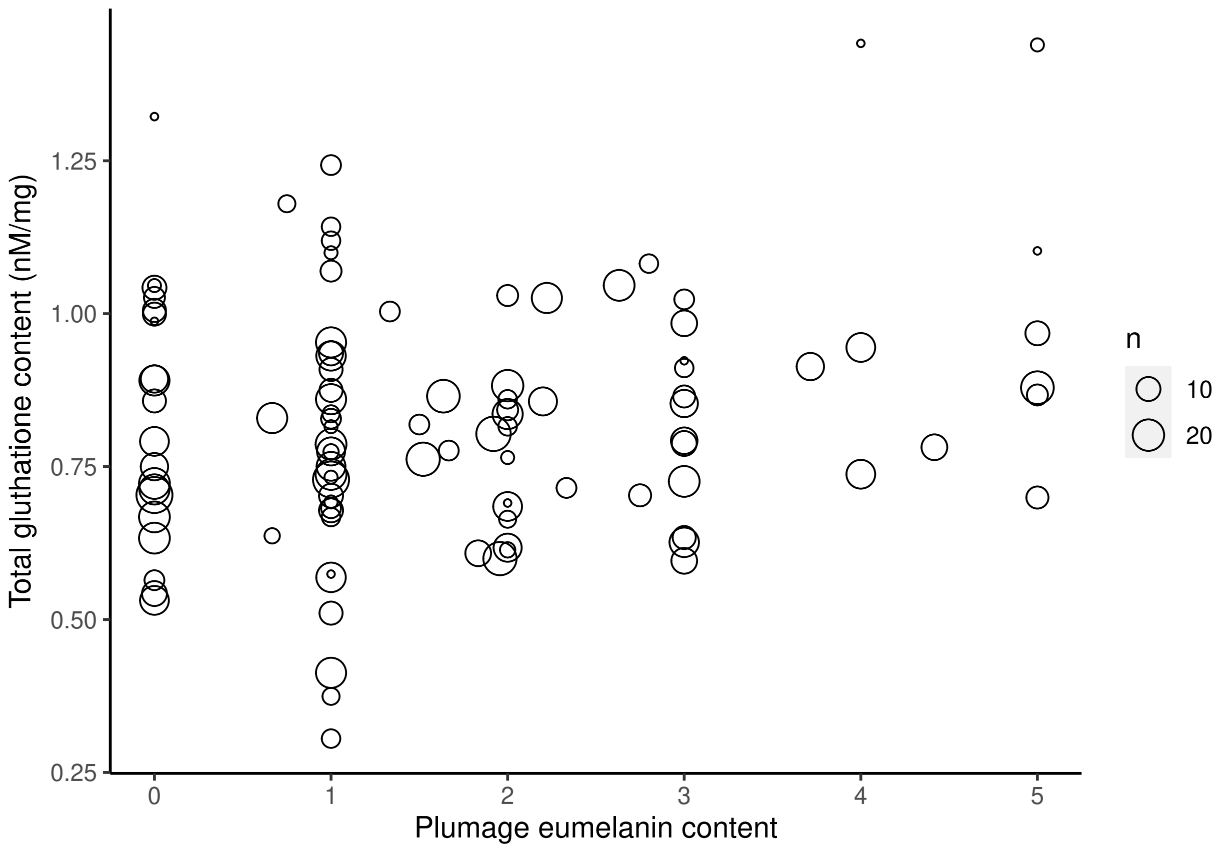


```
ggp18 <- data.tgsh %>%
  group_by(species) %>%
  summarize(species = species, yvalue = mean(log10tGSH), xvalue = mean(pheomelanin), n = n()) %>%
  distinct() %>%
  ggplot(aes(xvalue, yvalue, size = n)) +
  geom_point(shape =  21) +
  theme(panel.background = element_blank(),
        axis.line = element_line(color = "black")) +
  ylab(expression("Total gluthatione content (nM/mg)")) +
  xlab("Plumage pheomelanin content")
```

```
## `summarise()` has grouped output by 'species'. You can override using the
## `.groups` argument.
```

```
print(ggp18)
```

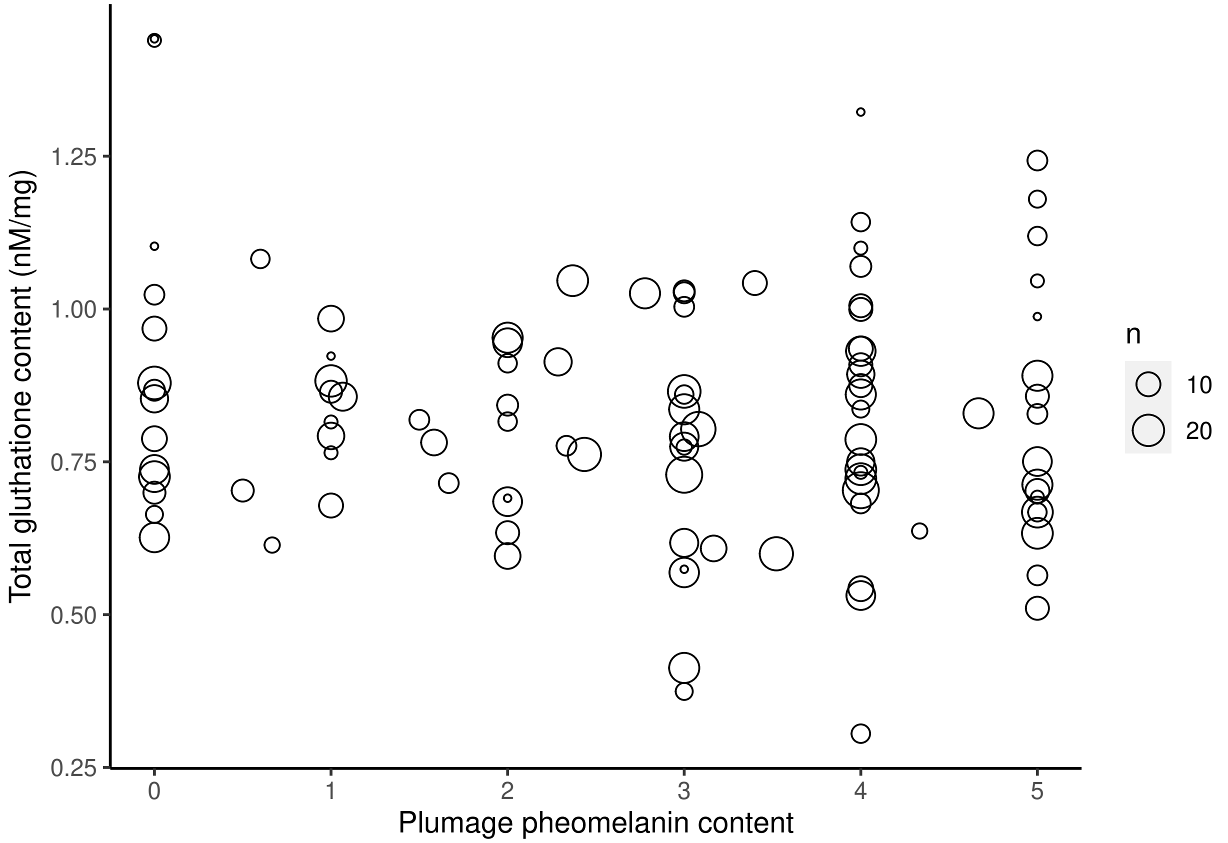


```
# ROM2 means vs. eumelanin and pheomelanin, all data

ggp19 <- data.rom %>%
 group_by(species) %>%
 summarize(species = species, yvalue = mean(ROM2), xvalue = mean(eumelanin), n = n()) %>%
 distinct() %>%
 ggplot(aes(xvalue, yvalue, size = n)) +
 geom_point(shape =  21) +
 theme(panel.background = element_blank(),
       axis.line = element_line(color = "black")) +
 ylab(expression("Reactive oxygen metabolites (mM H "[2]*"O"[2]*")")) +
 xlab("Plumage eumelanin content")
```

```
## `summarise()` has grouped output by 'species'. You can override using the
## `.groups` argument.
```

```
print(ggp19)
```

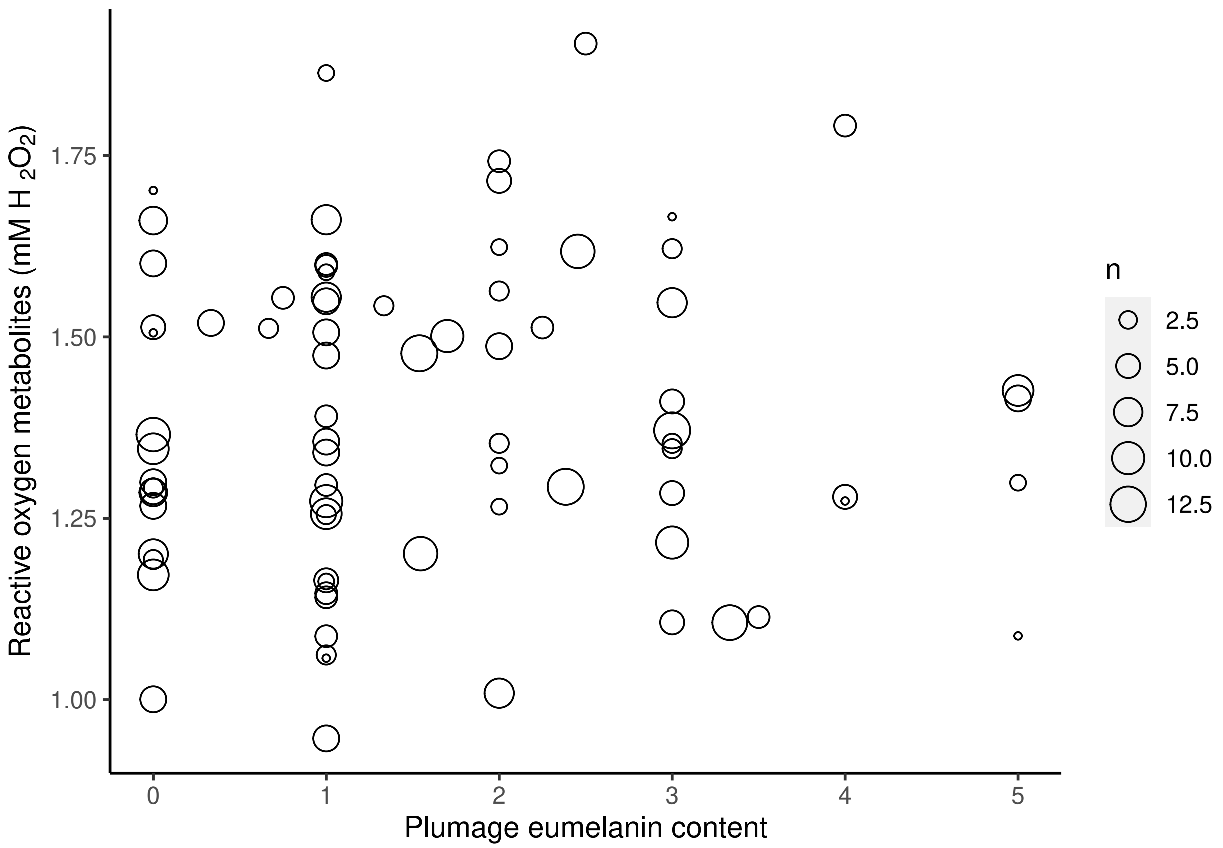


```
ggp20 <- data.rom %>%
 group_by(species) %>%
 summarize(species = species, yvalue = mean(ROM2), xvalue = mean(pheomelanin), n = n()) %>%
 distinct() %>%
 ggplot(aes(xvalue, yvalue, size = n)) +
 geom_point(shape =  21) +
 theme(panel.background = element_blank(),
       axis.line = element_line(color = "black")) +
 ylab(expression("Reactive oxygen metabolites (mM H "[2]*"O"[2]*")")) +
 xlab("Plumage pheomelanin content")
```

```
## ‘summarise()‘ has grouped output by ’species’. You can override using the
## ‘.groups‘ argument.
```

```
print(ggp20)
```
